# Evolution of Outcome Assessment in Chronic Low Back Pain Clinical Trials Throughout the 20th and 21st Centuries: A Scoping Review

**DOI:** 10.1002/msc.70270

**Published:** 2026-10-12

**Authors:** Daniel Santos Rocha, Alice Marques Moreira Lima, Ana Clara Chung‐Cardoso, Ana Paula Brito‐de‐Sousa, Anna Lívia Serejo‐da‐Silva, Marcelo Souza de Andrade, André Pontes‐Silva

**Affiliations:** ^1^ Postgraduate Program in Adult Health Federal University of Maranhão São Luís Maranhão Brazil; ^2^ Postgraduate Program in Physical Education NERD Research Group Federal University of Maranhão São Luís Maranhão Brazil

**Keywords:** musculoskeletal system, pain, patient‐reported outcomes, spine

## Abstract

**Objective:**

To identify, map, and synthesise the outcomes reported in patients with Chronic Low Back Pain (CLBP) and describe how these outcomes have changed over time throughout the 20th and 21st centuries.

**Methods:**

A scoping review was conducted according to the Joanna Briggs Institute methodology and reported following the PRISMA‐ScR guidelines. Four electronic databases (PubMed, Embase, PEDro, and Google Scholar) were searched for clinical trials published between 1975 and 2025. Five independent reviewers screened studies, extracted data, and categorised reported outcomes into conceptually related domains. Outcomes and measurement instruments were synthesised descriptively and narratively according to four chronological periods.

**Results:**

A total of 708 clinical trials met the eligibility criteria. Pain intensity remained the only outcome consistently reported throughout the entire study period. Over time, outcome assessment progressively expanded to include disability, physical function, physical performance, psychological health, quality of life, work‐related outcomes, sleep, fatigue, physical activity, healthcare utilization, and patient‐reported outcomes. Measurement instruments also evolved from predominantly symptom‐oriented scales to standardized multidimensional questionnaires and, more recently, objective digital technologies. Overall, outcome assessment shifted from a predominantly biomedical approach toward comprehensive biopsychosocial evaluation.

**Conclusions:**

Outcome assessment in CLBP clinical trials has undergone a marked conceptual evolution over the past 5 decades. These findings support the adoption of standardized, multidimensional, and patient‐centred outcome frameworks to improve the methodological quality, comparability, and clinical relevance of future clinical trials.

## 1 Introduction

CLBP is one of the most prevalent and disabling musculoskeletal conditions worldwide, representing a major contributor to years of living with disability and imposing substantial clinical, social, and economic burdens on healthcare systems. Recent estimates from the Global Burden of Disease Study indicate that the number of individuals living with low back pain will continue to increase over the coming decades due to population growth and ageing, reinforcing its position as one of the leading causes of disability globally (Ferreira et al. 2023; Gill et al. 2023).

Although traditionally interpreted as a predominantly biomechanical disorder, contemporary evidence recognises CLBP as a complex and multifactorial condition influenced by interactions among biological, psychological, behavioural, and social factors. Consequently, successful management extends beyond pain reduction and requires consideration of disability, physical functioning, psychological well‐being, participation, and overall quality of life. This paradigm shift has fundamentally changed both clinical practice and the design of rehabilitation research, encouraging investigators to evaluate broader dimensions of health and functioning rather than isolated symptoms (Balagué et al. 2012; Chou 2021; Patrick et al. 2014).

The growing complexity of CLBP has been accompanied by an exponential increase in clinical trials investigating pharmacological, surgical, exercise‐based, educational, behavioural, and multidisciplinary interventions. As therapeutic approaches have diversified, the range of outcomes used to evaluate treatment effectiveness has expanded considerably. Early studies primarily focused on pain intensity, whereas contemporary trials increasingly assess disability, physical performance, health‐related quality of life, psychological factors, work participation, patient satisfaction, sleep, fatigue, physical activity, and other patient‐reported outcomes. This expansion reflects the progressive adoption of multidimensional assessment frameworks that are more consistent with the biopsychosocial understanding of CLBP (Bastos et al. 2022; Owen et al. 2020; Thiveos et al. 2024).

Despite these advances, considerable heterogeneity remains in outcome selection across clinical trials. Different studies frequently evaluate distinct constructs using diverse measurement instruments, limiting direct comparisons between interventions, increasing methodological heterogeneity, and complicating evidence synthesis through systematic reviews and meta‐analyses. To address this challenge, international initiatives have proposed standardized Core Outcome Sets (COSs) for non‐specific low back pain, emphasising that all clinical trials should report a minimum set of essential outcome domains to improve comparability, reduce research waste, and facilitate evidence synthesis (Chiarotto et al. 2015, 2014).

Although recommendations regarding core outcome domains have become increasingly available, little attention has been given to understanding how outcome assessment has evolved throughout the history of CLBP research. Most previous studies have focused on recommending standardized outcomes or evaluating the uptake of core outcome sets rather than examining the historical evolution of outcome reporting itself. Consequently, it remains unclear when major outcome domains emerged, how assessment priorities changed over time, and how the transition from symptom‐oriented evaluation toward multidimensional biopsychosocial assessment has occurred across decades of clinical research. Understanding this historical evolution may provide valuable insights into changing research priorities, methodological developments, and the maturation of outcome assessment in CLBP clinical trials (Farragher et al. 2024; Sonmezer et al. 2021).

Comprehensive mapping of reported outcomes is also important for identifying underrepresented domains, informing future consensus initiatives, supporting the refinement of core outcome sets, and guiding researchers towards more standardized and clinically meaningful outcome selection. Furthermore, documenting the historical evolution of measurement instruments may help contextualise methodological advances and explain variations in outcome reporting observed across different periods of rehabilitation research.

Therefore, what outcomes have been reported in patients with CLBP, and how have these outcomes evolved throughout the 20th and 21st centuries? As such, we aimed to identify, map, and synthesise the outcomes reported in patients with CLBP and describe how these outcomes have changed over time throughout the 20th and 21st centuries.

## 2 Methods

### 2.1 Study Design

This scoping review was conducted in accordance with the methodological framework proposed by the Joanna Briggs Institute for scoping reviews, which provides systematic guidance for defining the research question, establishing eligibility criteria, identifying relevant evidence, selecting studies, extracting data, and synthesising findings. A scoping review methodology was considered appropriate because the objective was to comprehensively map the outcomes reported in clinical trials involving patients with CLBP and to examine how these outcomes have evolved over time, rather than to evaluate the effectiveness of specific interventions (Chapter 11: Scoping Reviews, 2020; Tricco et al. 2018).

The review was designed and reported according to the Preferred Reporting Items for Systematic Reviews and Meta‐Analyses Extension for Scoping Reviews (PRISMA‐ScR), ensuring methodological transparency, reproducibility, and completeness throughout all stages of the review (Chapter 11: Scoping Reviews, 2020; Tricco et al. 2018).

### 2.2 Research Question

The research question was developed using the Population–Concept–Context framework recommended by the Joanna Briggs Institute for scoping reviews.

- Population: patients with CLBP.
- Concept: outcomes reported in clinical trials and the instruments used to assess these outcomes.
- Context: the evolution of outcome assessment throughout the 20th and 21st centuries.

Accordingly, this review sought to answer the following research question: What outcomes have been reported in clinical trials involving patients with CLBP, and how have these outcomes evolved throughout the 20th and 21st centuries?

### 2.3 Eligibility Criteria

Studies were considered eligible if they met all of the following criteria:

1. Included participants diagnosed with CLBP, defined as low back pain persisting for at least 12 weeks (3 months). When symptom duration was not explicitly reported, studies were considered eligible when the study authors explicitly described the population as having chronic low back pain (Pontes‐Silva et al. 2026).
2. Reported at least one clinical, functional, psychosocial, behavioural, physiological, or patient‐reported outcome;
3. Described the measurement instrument, assessment method, or test used to evaluate the reported outcome(s);
4. Employed a clinical trial design;
5. Studies published between 1975 and 2025 were eligible. The publication period was restricted to complete calendar years through 2025 to ensure a consistent temporal analysis across the predefined chronological periods; and
6. Were published in English.

Studies were excluded if they were literature reviews, pilot studies, study protocols, cohort studies, prevalence studies, cross‐sectional or other observational studies, editorials, letters to the editor, dissertations, theses, duplicate publications, incomplete reports, studies without accessible full texts, or studies that did not address the research question.

### 2.4 Information Sources and Search Strategy

A comprehensive literature search was conducted in four electronic databases: PubMed, Embase, PEDro, and Google Scholar. To maximise retrieval of potentially relevant studies, the reference lists of all included articles were manually screened for additional eligible publications.

The search strategy was developed using controlled vocabulary (e.g., MeSH) and free‐text terms combined with Boolean operators (AND and OR). Search terms related to chronic low back pain and clinical trials were adapted to the indexing system and search syntax of each database. The complete, database‐specific search strategies, including search fields, Boolean operators, truncation, and database‐specific syntax, are provided in Table 1. All electronic searches were performed on May 20, 2026.

**TABLE 1:** Search strategy.

Database	Terms	∑	Period
PubMed (MeSH)	Low back pain; low back pain, mechanical; low back pain, posterior compartment; low back pain, postural; low back pain, recurrent; clinical trial	(((((“Low back pain”[MeSH terms]) OR (“low back pain, mechanical”[MeSH terms])) OR (“low back pain, posterior compartment”[MeSH terms])) OR (“low back pain, postural”[MeSH terms])) OR (“low back pain, recurrent”[MeSH terms])) AND (“clinical trial”[Publication type])	May 20, 2026
Google scholar (free‐text terms)	Low back pain; clinical trial	Intitle: “Low back pain” AND “clinical trial”	
PEDro (PEDro search fields)	Low back pain; lumbar spine; clinical trial	Abstract & title: Low back pain Body part: Lumbar spine, sacro‐iliac joint or pelvis Method: Clinical trial	
Embase (Emtree)	Low back pain; clinical trial	‘Low back pain’/exp AND ‘clinical trial’	

*Note:* MeSH, Medical Subject Headings, is the controlled vocabulary used for indexing in PubMed. Emtree is the controlled vocabulary used in Embase. Free‐text terms were searched using the database‐specific syntax and fields described.

### 2.5 Study Selection

All retrieved records were exported to Rayyan, a web‐based platform designed for systematic and scoping reviews, where duplicate records were automatically identified and manually verified before removal.

Study selection was conducted independently by all five reviewers in two sequential phases: title/abstract screening followed by full‐text assessment. Each reviewer independently assessed all records at both stages. Disagreements regarding study eligibility were discussed among the reviewers until consensus was reached.

### 2.6 Data Extraction

Data extraction was independently performed by five reviewers between May 20 and May 24, 2026, using a standardized data extraction form specifically developed for this review. All extracted information was recorded and managed using Microsoft Excel. The following information was extracted from each included study:

- first author;
- year of publication;
- study design;
- characteristics of the study population;
- publication period;
- primary and secondary outcomes;
- instruments, questionnaires, clinical tests, or performance measures used to assess each outcome.

Whenever a study reported multiple outcomes, all outcomes and their respective assessment instruments were extracted to ensure a comprehensive mapping of outcome reporting across the literature. Data extraction was independently performed by the five reviewers. Any discrepancies identified during data extraction were discussed among the reviewers and resolved by consensus.

### 2.7 Data Synthesis and Presentation

Extracted data were synthesised using a descriptive and narrative approach, consistent with the objectives of a scoping review. Because the purpose of this review was to map the breadth and evolution of reported outcomes rather than evaluate intervention effectiveness, no quantitative synthesis or meta‐analysis was performed.

Following data extraction, individual outcomes were classified into conceptually related domains according to the primary construct assessed by the outcome measure. The classification was performed through an iterative review of the extracted outcomes and their corresponding measurement instruments, with outcomes assessing similar constructs grouped within the same domain. For example, measures assessing pain severity or intensity were classified under pain intensity, whereas measures assessing limitations in daily activities were classified under disability. These domains included, but were not limited to, pain intensity, disability, physical function, physical performance, muscle performance, mobility, quality of life, psychological outcomes, work‐related outcomes, sleep, fatigue, physical activity, patient satisfaction, healthcare utilization, and other patient‐reported outcomes. This categorisation facilitated the comparison of outcome reporting across studies while reducing redundancy among similar constructs. For the descriptive synthesis, the frequency of reporting within each chronological period was categorised as rare (< 10%), occasional (10%–24%), common (25%–49%), or very common (≥ 50%) of the clinical trials in that period. The terms “emerging” and “increasing” were used to describe temporal patterns of outcome reporting rather than absolute frequency.

To investigate temporal trends, studies were organised into four chronological periods based on publication year: 1975–1989, 1990–2009, 2010–2019, and 2020–2025. These periods were defined to represent distinct phases in the historical evolution of outcome assessment, ranging from predominantly symptom‐oriented assessment in the earliest period to increasingly functional, biopsychosocial, and patient‐centred assessment in subsequent periods. The unequal duration of the periods reflects these distinct phases of historical evolution rather than equal‐length time intervals. Because the chronological periods differ in duration and number of included studies, comparisons across periods were interpreted descriptively rather than as direct measures of publication rates over time.

Results were synthesised in chronological order to identify changes in the diversity, complexity, and conceptual scope of outcome assessment over time. Particular emphasis was placed on identifying emerging outcome domains, shifts in assessment priorities, and the progressive transition from predominantly symptom‐oriented evaluation toward multidimensional biopsychosocial assessment. Tables, conceptual figures, heatmaps, and network diagrams were used to summarise the temporal evolution of reported outcomes and the measurement instruments employed throughout the 20th and 21st centuries.

## 3 Results

### 3.1 Overall Evolution of Outcome Assessment in CLBP Clinical Trials

A total of 708 clinical trials published between 1975 and 2025 met the eligibility criteria and were included in this scoping review (Figure 1; Supporting Information S1). Across the four chronological periods, the frequency of reporting of major outcome domains varied substantially. Pain intensity was reported in 23/33 (69.7%), 88/146 (60.3%), 103/160 (64.4%), and 257/369 (69.6%) studies in 1975–1989, 1990–2009, 2010–2019, and 2020–2025, respectively. Disability was reported in 3/33 (9.1%), 61/146 (41.8%), 61/160 (38.1%), and 130/369 (35.2%) studies, respectively. Physical function, quality of life, and psychological outcomes were less frequently reported in the earlier periods and showed greater representation in later periods, being reported in 0/33 (0.0%) to 19/369 (5.1%), 0/33 (0.0%) to 20/369 (5.4%), and 1/33 (3.0%) to 33/369 (8.9%) studies, respectively.

**FIGURE 1:**
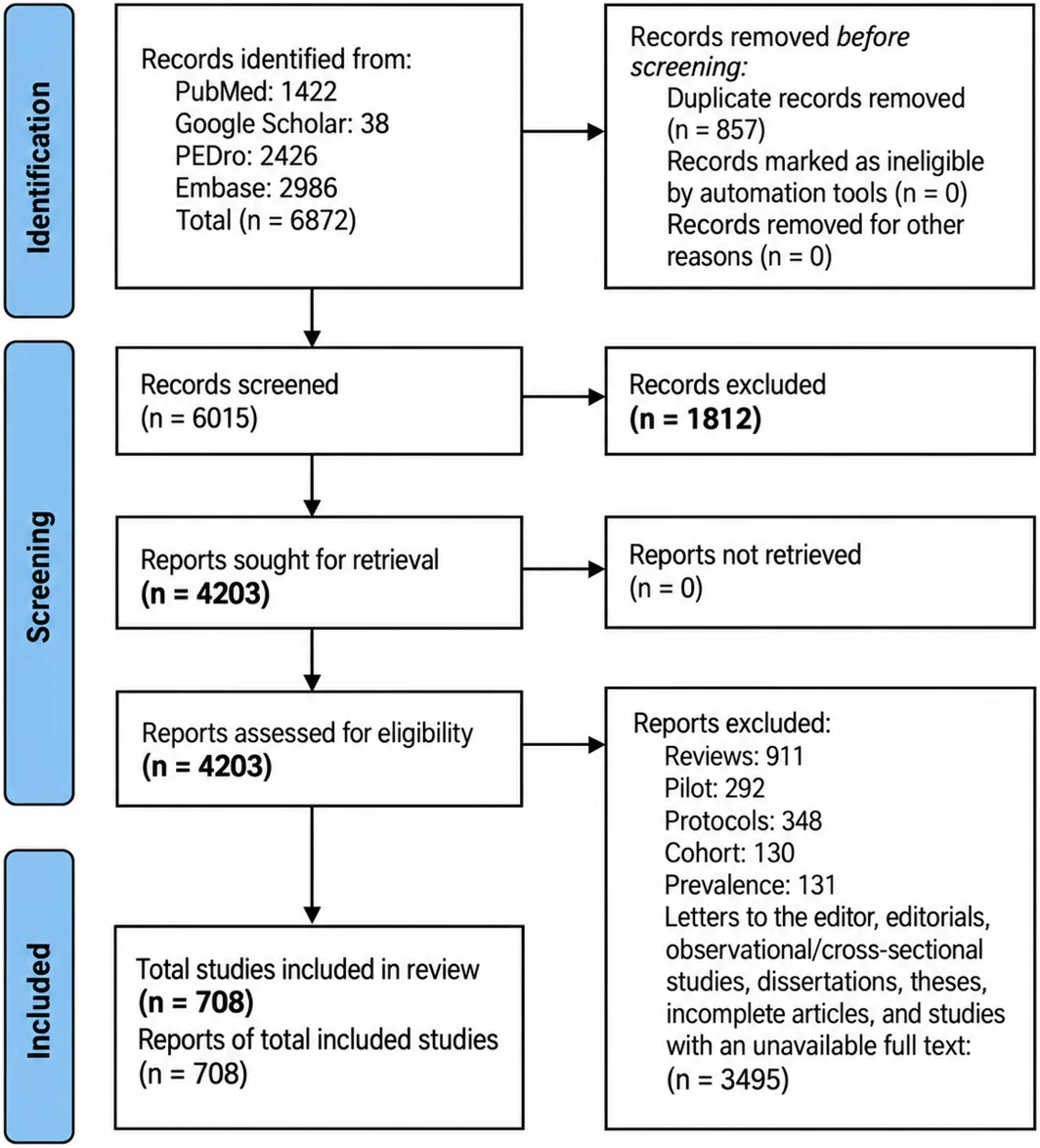
Flow diagram of the study selection process. A total of 6872 records were identified from PubMed, Google Scholar, PEDro, and Embase, and 857 duplicate records were removed before screening, leaving 6015 records for title and abstract screening. During this first screening stage, 1812 records were excluded, and 4203 reports were subsequently sought for retrieval and assessed for eligibility through full‐text assessment. Of these, 3495 reports were excluded based on the predefined eligibility criteria, resulting in 708 clinical trials included in the scoping review.

Collectively, these studies demonstrated a substantial expansion in both the number and diversity of outcomes assessed over the past 5 decades. While pain intensity remained the predominant endpoint throughout the entire study period, the overall structure of outcome assessment evolved considerably, reflecting important changes in the conceptualisation of CLBP. Early clinical trials primarily focused on symptom reduction, whereas more recent studies increasingly incorporated measures of physical function, disability, psychosocial health, quality of life, participation, and patient‐centred outcomes. This temporal evolution indicates that outcome assessment has progressively shifted from a predominantly biomedical perspective towards a multidimensional framework that recognises CLBP as a complex condition involving biological, psychological, functional, and social components.

An additional pattern observed throughout the evidence synthesis was the progressive increase in the number of outcomes evaluated within individual studies. Earlier trials commonly reported only one or two primary endpoints, usually limited to pain intensity and, occasionally, spinal mobility or disability. In contrast, contemporary clinical trials frequently combined multiple outcome domains within the same investigation, simultaneously assessing symptom severity, functional status, physical performance, psychological characteristics, quality of life, treatment adherence, and participation‐related measures. This accumulation of outcomes reflects increasing recognition that treatment effectiveness cannot be adequately captured through symptom reduction alone.

### 3.2 Initial Phase: Predominance of Symptom‐Oriented Outcomes (1975–1989)

The earliest clinical trials were characterised using a predominantly symptom‐oriented approach to treatment evaluation. During this period, pain intensity represented the principal measure of intervention effectiveness and was consistently reported as the primary endpoint across nearly all studies. The emphasis placed on pain reflects the prevailing biomedical model of musculoskeletal disorders, in which clinical improvement was largely defined by reductions in symptom severity. Consequently, relatively little attention was given to broader aspects of patient functioning or health status.

Beyond pain intensity, only a limited number of additional outcomes were identified during this period. Disability was occasionally evaluated, although it had not yet become a routine component of clinical assessment. Similarly, measures of spinal mobility, muscle activity, muscle strength, and range of motion appeared sporadically across individual studies. Psychological constructs such as emotional distress or depression were rarely incorporated, and patient‐reported outcomes addressing quality of life, social participation, or work‐related functioning were largely absent. The temporal expansion of the principal outcome domains across subsequent decades is illustrated in Figure 2.

**FIGURE 2:**
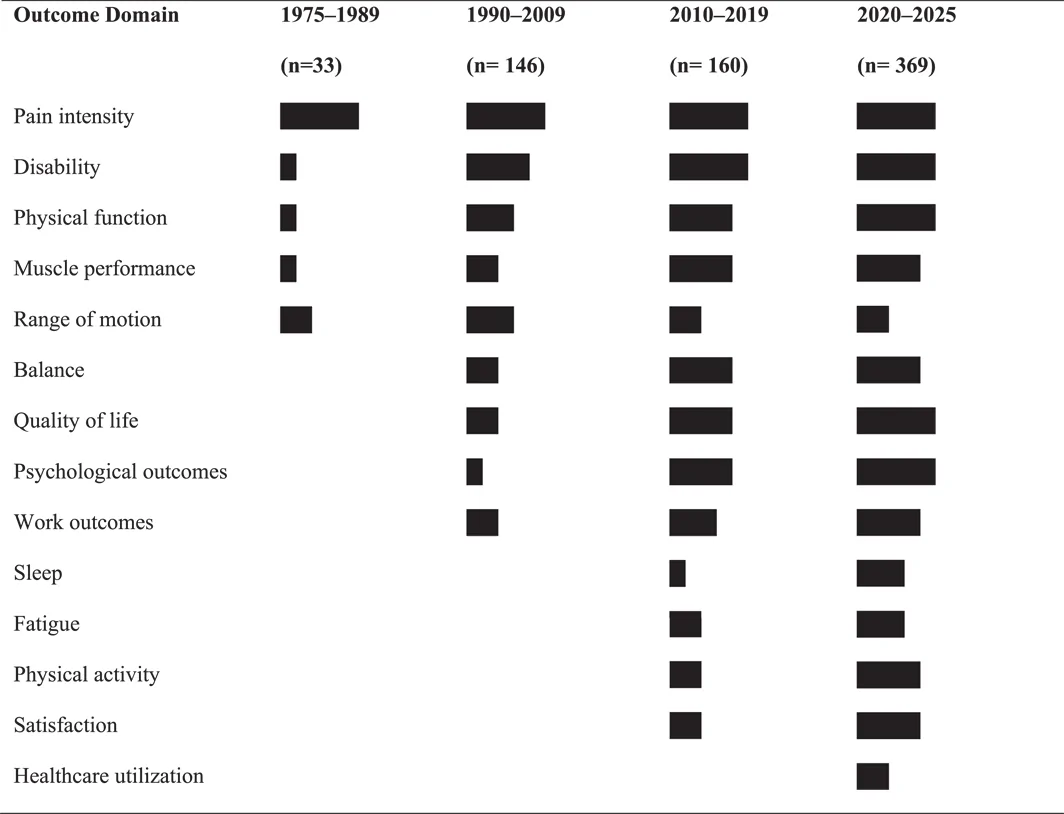
Evolution of outcome domains reported in chronic low back pain clinical trials between 1975 and 2025. Heatmap illustrating the temporal distribution of the major outcome domains across four study periods. Colour intensity represents the relative frequency with which each domain was reported. Pain intensity remained consistently reported throughout the entire study period, whereas disability, physical function, quality of life, psychological outcomes, sleep, and patient‐centred measures progressively emerged and became increasingly incorporated into contemporary clinical trials.

### 3.3 Expansion Towards Disability and Functional Recovery (1990–2009)

Beginning in the early 1990s, a marked expansion in outcome assessment became evident. Although pain intensity continued to represent the most frequently reported endpoint, disability emerged as a central outcome domain and progressively became one of the most consistently evaluated measures across clinical trials. Rather than assessing symptom reduction alone, investigators increasingly sought to determine whether improvements in pain translated into enhanced functional capacity and reduced activity limitations. This shift represented an important conceptual advance, recognising that clinical recovery extends beyond changes in pain intensity.

During this period, several additional domains became increasingly represented in the literature. Physical function, spinal mobility, muscle performance, flexibility, endurance, and balance were more frequently incorporated as indicators of treatment effectiveness. Likewise, health‐related quality of life began to appear with greater regularity, reflecting growing interest in the broader impact of CLBP on patients' daily lives. Work‐related outcomes, including return to work, work absenteeism, occupational disability, and productivity, also emerged during this period, highlighting increasing awareness of the socioeconomic burden associated with persistent low back pain. Collectively, these findings demonstrate that outcome assessment expanded beyond symptom evaluation to include measures reflecting physical functioning and participation in everyday activities.

### 3.4 Consolidation of Multidimensional Outcome Assessment (2010–2019)

Clinical trials published during the 2010s demonstrated a substantial increase in both the diversity and complexity of reported outcomes. Pain intensity and disability remained the two dominant endpoints; however, these measures were increasingly complemented by a broad range of additional domains intended to capture the multidimensional nature of CLBP. Rather than relying on isolated indicators of treatment success, investigators frequently implemented comprehensive assessment batteries integrating clinical, functional, behavioural, and psychosocial measures within individual studies.

Several patient‐reported outcomes became routinely incorporated during this period, including health‐related quality of life, perceived recovery, treatment satisfaction, self‐efficacy, and global impression of change. Simultaneously, physical performance measures—including walking capacity, balance, muscle endurance, movement quality, trunk muscle function, and functional performance tests—became increasingly common. The literature also demonstrated substantial growth in the assessment of psychological constructs associated with chronic pain, including fear of movement, pain catastrophizing, anxiety, depression, pain beliefs, and psychological distress. This expansion reflects increasing acceptance of the biopsychosocial model of CLBP and recognition that psychological and behavioural factors substantially influence treatment outcomes, symptom persistence, and functional recovery.

### 3.5 Comprehensive Biopsychosocial Assessment and Emergence of Patient‐Centred Outcomes (2020–2025)

The most recent clinical trials demonstrated the greatest diversity of reported outcomes throughout the study period. Contemporary investigations routinely evaluated multiple domains simultaneously, combining measures of symptom severity, disability, physical function, psychological health, participation, and patient‐reported outcomes within a single study. Although pain intensity remained the most frequently assessed endpoint, it was rarely evaluated in isolation, emphasising the increasing complexity of outcome assessment in CLBP research.

Physical outcomes expanded beyond traditional measures of pain and disability to include muscle strength, physical performance, movement quality, gait characteristics, motor control, balance, flexibility, physical activity levels, and objective biomechanical assessments. At the same time, psychological outcomes became firmly established components of clinical evaluation, encompassing depression, anxiety, fear‐avoidance beliefs, pain catastrophizing, resilience, self‐efficacy, pain acceptance, emotional well‐being, and other cognitive‐behavioural constructs associated with chronic pain. Sleep quality, fatigue, and broader indicators of health status also emerged more consistently during this period, reflecting increasing recognition of their contribution to symptom burden and long‐term prognosis.

Patient‐centred outcomes likewise became increasingly prominent. Recent studies frequently evaluated treatment satisfaction, adherence, perceived recovery, healthcare utilization, return to work, work productivity, social participation, and health‐related quality of life. Rather than focussing exclusively on symptom improvement, contemporary clinical trials have increasingly emphasised outcomes that reflect patients' lived experiences, functional independence, and participation in meaningful daily activities. This broadening of outcome assessment illustrates the continued evolution of CLBP research towards a more holistic understanding of treatment effectiveness.

### 3.6 Temporal Evolution of Major Outcome Domains

Throughout the entire study period, several consistent temporal patterns emerged regarding the evolution of outcome assessment. Pain intensity remained the only outcome that was consistently reported across all decades, maintaining its position as the principal indicator of treatment effectiveness despite substantial changes in the broader assessment framework. Disability progressively evolved from an infrequently reported secondary endpoint into one of the core outcome domains routinely evaluated alongside pain intensity, becoming an essential component of clinical trials from the 1990s onward. The temporal evolution of the major outcome domains is summarised in Table 2.

**TABLE 2:** Temporal evolution of major outcome domains in chronic low back pain clinical trials.

Outcome domain	1975–1989	1990–2009	2010–2019	2020–2025	Temporal trend
Pain intensity	Primary outcome	Primary outcome	Primary outcome	Primary outcome	Stable
Disability	Occasional	Common (25%–49%)	Very common (≥ 50%)	Very common (≥ 50%)	↑
Physical function	Rare	Increasing	Common	Very common (≥ 50%)	↑
Physical performance	Rare	Emerging	Common	Very common (≥ 50%)	↑
Muscle performance	Rare	Increasing	Common	Common	↑
Mobility/ROM	Occasional	Common	Common	Common	→
Quality of life	Rare	Emerging	Common (25%–49%)	Very common (≥ 50%)	↑↑
Psychological outcomes	Rare	Occasional	Common (25%–49%)	Very common (≥ 50%)	↑↑↑
Work‐related outcomes	Absent	Emerging	Common	Common	↑↑
Patient satisfaction	Absent	Rare	Emerging	Common (25%–49%)	↑↑
Sleep	Absent	Absent	Emerging	Common (25%–49%)	↑↑↑
Fatigue	Absent	Rare	Emerging	Common (25%–49%)	↑↑
Physical activity	Absent	Rare	Emerging	Common (25%–49%)	↑↑
Healthcare utilization	Absent	Absent	Rare	Emerging	↑

*Note:* Rare = < 10%; occasional = 10%–24%; common = 25%–49%; very common = ≥ 50% of clinical trials within each chronological period. “Emerging” and “increasing” describe temporal patterns of outcome reporting rather than absolute frequency.

Overall, the findings demonstrate a clear temporal transition in the evaluation of CLBP. While early clinical trials primarily assessed biological impairment through symptom‐oriented outcomes, contemporary investigations routinely incorporate multidimensional outcome frameworks encompassing body functions, activity limitations, participation, psychosocial health, and patient‐reported experiences. This progressive expansion reflects the maturation of CLBP research and its alignment with contemporary biopsychosocial models of health and functioning. The evolution of the most frequently used measurement instruments is summarised in Table 3 and Figure 3.

**TABLE 3:** Evolution of the principal measurement instruments used across major outcome domains.

Outcome domain	1975–1989	1990–2009	2010–2019	2020–2025
Pain intensity	VAS	VAS, NRS	NRS predominates	NRS predominates
Disability	Roland‐morris, oswestry (emerging)	Oswestry, roland‐morris	Oswestry, roland‐morris	Oswestry, roland‐morris
Quality of life	SIP (sporadic)	SF‐36	SF‐36, EQ‐5D	EQ‐5D, PROMIS
Psychological outcomes	BDI (sporadic)	FABQ, BDI	TSK, PCS, FABQ, HADS	PROMIS, PCS, TSK, HADS
Physical performance	Simple functional tests	Walking tests	Functional performance batteries	Performance batteries + wearable technologies
Physical activity	Self‐report questionnaires	IPAQ	Accelerometers + questionnaires	Wearables + accelerometers
Sleep	Rare self‐report	PSQI (sporadic)	PSQI	PSQI + objective monitoring

Abbreviations: BDI, Beck Depression Inventory; EQ‐5D, EuroQol 5‐Dimension Questionnaire; FABQ, Fear‐Avoidance Beliefs Questionnaire; HADS, Hospital Anxiety and Depression Scale; IPAQ, International Physical Activity Questionnaire; NRS, Numeric Rating Scale; PCS, Pain Catastrophizing Scale; PROMIS, Patient‐Reported Outcomes Measurement Information System; PSQI, Pittsburgh Sleep Quality Index; ROM, Range of Motion; SF‐36, 36‐Item Short Form Health Survey; SIP, Sickness Impact Profile; TSK, Tampa Scale for Kinesiophobia; VAS, Visual Analog Scale.

**FIGURE 3:**
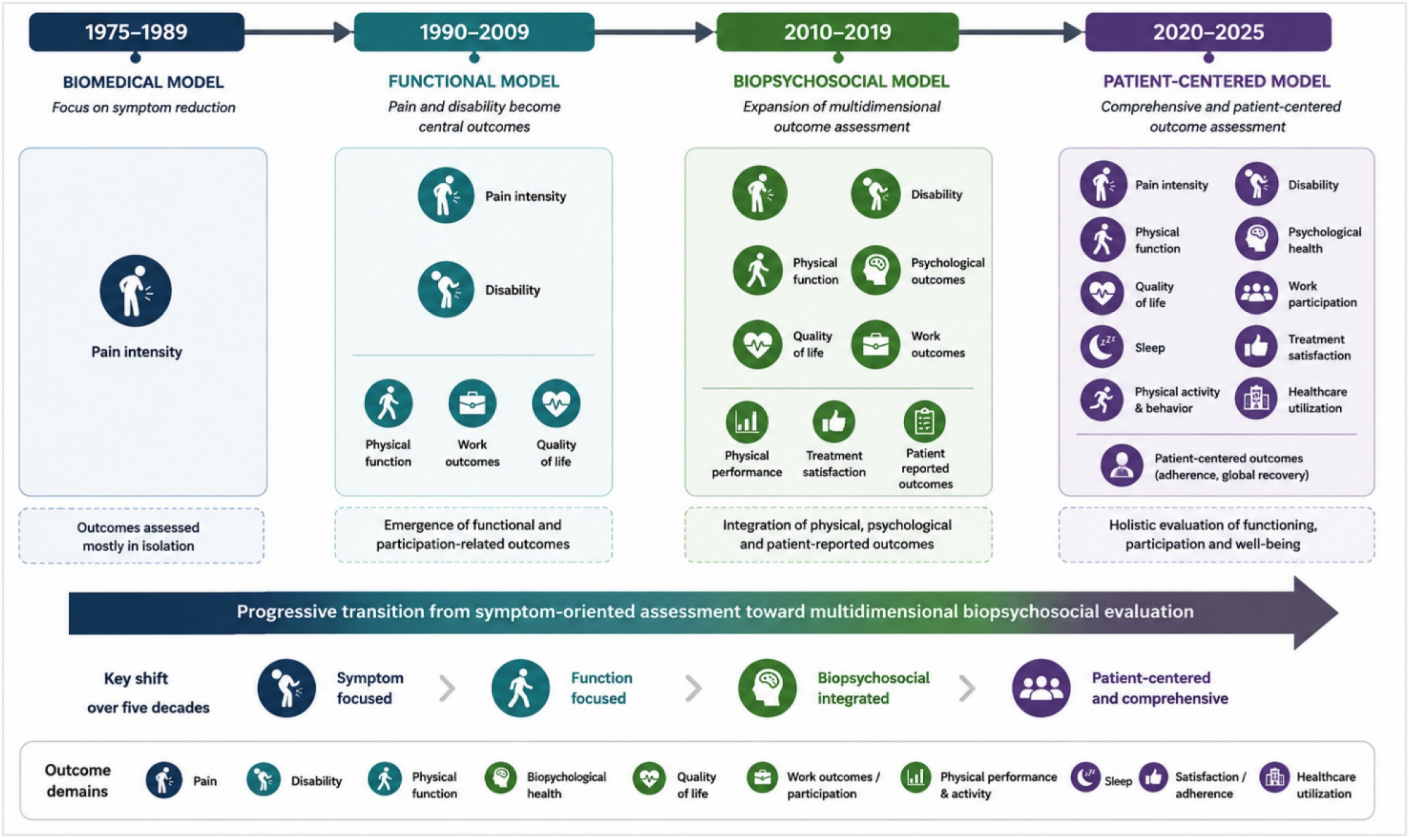
Conceptual evolution of outcome assessment in chronic low back pain clinical trials. The figure illustrates the progressive expansion of outcome assessment over the past 5 decades. Early studies primarily focused on symptom reduction, whereas contemporary trials increasingly incorporate functional, psychosocial, behavioural, participation‐related, and patient‐centred outcomes, reflecting the transition from a biomedical toward a biopsychosocial model of chronic low back pain.

## 4 Discussion

### 4.1 Principal Findings

The present scoping review provides, to our knowledge, the first comprehensive historical mapping of outcome assessment in clinical trials involving patients with CLBP over a 50‐year period. By synthesising evidence from 708 clinical trials published between 1975 and 2025, this review demonstrates that outcome assessment has undergone a remarkable conceptual transformation. Although pain intensity remained the only outcome consistently reported throughout all decades, the breadth of evaluated domains expanded substantially over time, progressively incorporating disability, physical function, physical performance, psychological health, quality of life, work participation, sleep, fatigue, and other patient‐centred outcomes. Importantly, this evolution did not represent the replacement of traditional outcomes but rather their cumulative integration into increasingly comprehensive assessment frameworks.

Consequently, contemporary clinical trials have shifted from evaluating isolated symptom reduction to examining multiple dimensions of health and functioning simultaneously, reflecting the growing recognition that CLBP is a complex multidimensional condition requiring multidimensional evaluation. These findings closely parallel the evolution of contemporary pain science, which increasingly recognises that persistent musculoskeletal pain emerges from dynamic interactions among biological, psychological, behavioural, and social factors rather than from isolated structural abnormalities (Cuomo et al. 2021).

One of the most important findings of this review is that the historical evolution of outcome assessment mirrors the maturation of the scientific understanding of CLBP itself. During the earliest decades, clinical trials were largely guided by a biomedical paradigm in which treatment success was primarily defined by reductions in pain intensity. As knowledge regarding pain mechanisms expanded, investigators increasingly recognized that symptom improvement alone provides only a partial representation of clinical recovery. The subsequent incorporation of disability, physical functioning, quality of life, psychological health, participation, and patient‐reported outcomes reflects a broader conceptual shift towards evaluating how patients function, adapt, and participate in daily life despite persistent symptoms. Rather than representing methodological inflation, the increasing diversity of assessed outcomes likely reflects greater alignment between clinical trial methodology and the multifaceted nature of CLBP.

This interpretation is supported by contemporary epidemiological evidence demonstrating that low back pain remains the leading cause of years of disability worldwide and that its societal burden extends far beyond pain itself, affecting work capacity, mental health, physical activity, healthcare utilization, and quality of life (Ferreira et al. 2023; Gill et al. 2023).

### 4.2 From a Biomedical Paradigm to Multidimensional Assessment

The temporal evolution identified in this review closely reflects one of the most important paradigm shifts in musculoskeletal rehabilitation over the past 5 decades: the transition from a predominantly biomedical model towards a biopsychosocial understanding of CLBP. During the 1970s and 1980s, clinical trials were largely designed under the assumption that pain was a direct consequence of structural tissue injury and that successful treatment should therefore be measured primarily by reductions in pain intensity.

Consequently, outcome assessment focused almost exclusively on symptom severity, with little consideration given to disability, psychosocial health, or participation. As demonstrated by our findings, this perspective gradually changed throughout the following decades, with clinical trials progressively incorporating functional, psychological, and patient‐centred outcomes. Rather than representing merely an expansion of measurement strategies, this evolution reflects a fundamental change in how CLBP itself is conceptualised. Contemporary pain science recognises that persistent pain results from complex interactions among peripheral nociception, central nervous system adaptations, cognitive and emotional processes, behavioural responses, and environmental influences, making multidimensional assessment an essential prerequisite for understanding treatment effectiveness (Buchbinder et al. 2020).

This conceptual transformation has also influenced international recommendations for the assessment of musculoskeletal disorders. The International Classification of Functioning, Disability and Health shifted the focus of health evaluation from disease‐centred impairments towards the interaction between body functions, activities, participation, personal factors, and environmental influences. The progressive incorporation of disability, physical function, work participation, quality of life, and psychological health observed in our review strongly parallels this framework, suggesting that outcome assessment in CLBP clinical trials has gradually become more aligned with contemporary models of human functioning. Importantly, this transition does not diminish the clinical relevance of pain intensity. Instead, it recognises that pain represents only one dimension of recovery and that meaningful clinical improvement should also encompass the patient's ability to perform daily activities, participate in society, and achieve personally valued goals. Consequently, the multidimensional assessment frameworks identified in more recent decades appear to better capture the complexity of CLBP than the symptom‐oriented evaluations that predominated during the earliest clinical trials (Delitto et al. 2012; Silva et al. 2024).

### 4.3 The Emergence of Disability as a Core Outcome Domain

Among all outcome domains identified in this review, disability exhibited one of the most pronounced historical transformations. Although infrequently reported during the earliest decades, disability progressively evolved into one of the principal outcomes assessed in clinical trials and, from the 1990s onward, became routinely evaluated alongside pain intensity. This finding is particularly noteworthy because it illustrates a shift in the definition of treatment success itself. Earlier studies primarily asked whether an intervention reduced pain, whereas more recent investigations increasingly seek to determine whether patients regain function, improve daily performance, and reduce activity limitations. Such a transition reflects growing recognition that reductions in pain do not necessarily translate into meaningful improvements in functional independence or participation. Indeed, individuals with similar levels of pain intensity often present markedly different levels of disability, highlighting the importance of evaluating both constructs simultaneously rather than considering either in isolation (Chiarotto et al. 2015).

Recent evidence further supports this biopsychosocial interpretation of disability in chronic low back pain. Canli and Özüdoğru (2026) examined the contribution of biopsychosocial factors to disability in individuals with chronic nonspecific low back pain, reinforcing the relevance of considering disability as a multidimensional construct influenced by factors beyond pain intensity alone. These findings provide contemporary support for the broader conceptual shift identified in our review, in which disability increasingly became integrated with physical, psychological, and contextual dimensions of patient functioning (Canli and Özüdoğru 2026).

The central role acquired by disability in contemporary clinical trials has important implications for both research and clinical practice. Disability measures integrate multiple dimensions of health, including physical capacity, behavioural adaptation, psychosocial influences, and environmental demands, thereby providing a broader representation of the patient's overall health status than pain intensity alone. Furthermore, disability outcomes frequently demonstrate greater responsiveness to rehabilitation interventions that target movement behaviour, exercise adherence, self‐management, and cognitive‐behavioural strategies.

Consequently, the increasing emphasis placed on disability observed throughout this review likely reflects the evolution of rehabilitation itself—from interventions primarily aimed at symptom control towards strategies focused on restoring function, promoting self‐efficacy, and improving long‐term participation. This interpretation is consistent with current international clinical practice guidelines, which recommend that rehabilitation programs prioritise improvements in function and quality of life rather than exclusively pursuing complete pain relief (Kostadinović et al. 2020; Wang et al. 2012).

### 4.4 The Emergence of Patient‐Reported Outcomes and Psychological Assessment

One of the most remarkable findings of this review was the progressive incorporation of psychological and patient‐reported outcomes into clinical trials, particularly after 2010. While early investigations were almost exclusively centred on observable clinical signs and symptom severity, contemporary studies increasingly evaluate constructs such as fear of movement, pain catastrophizing, anxiety, depression, self‐efficacy, resilience, pain acceptance, perceived recovery, and treatment satisfaction.

This evolution reflects a broader recognition that patients' perceptions, beliefs, expectations, and emotional responses substantially influence pain persistence, treatment adherence, functional recovery, and long‐term prognosis. Rather than representing secondary outcomes, these variables have become integral components of contemporary rehabilitation research because they capture dimensions of health that cannot be adequately explained through physical impairment alone. The increasing incorporation of patient‐reported outcome measures (PROMs) identified in this review therefore mirrors the growing emphasis on patient‐centred care, in which treatment effectiveness is evaluated not only by clinical improvement but also by outcomes that are meaningful from the patient's perspective (Churruca et al. 2021; Fennelly et al. 2018).

The expansion of psychological assessment observed in our findings is also consistent with accumulating evidence demonstrating that cognitive and emotional factors are among the strongest predictors of pain‐related disability and long‐term outcomes in CLBP. Fear‐avoidance beliefs, pain catastrophizing, depression, and low self‐efficacy have repeatedly been associated with reduced physical activity, greater disability, delayed return to work, and poorer responses to rehabilitation interventions. Consequently, their increasing inclusion within clinical trials likely reflects not only methodological refinement but also a growing appreciation that effective rehabilitation requires simultaneous assessment of biological, psychological, and behavioural determinants of recovery (Huang et al. 2025; Jagadish and Hadya Ananda 2025; Ochsenkuehn et al. 2022).

Importantly, our review suggests that psychological outcomes were progressively incorporated alongside traditional measures rather than replacing them, reinforcing the notion that multidimensional assessment provides a more comprehensive characterisation of treatment effects than any single outcome alone. This observation supports recent recommendations advocating routine integration of PROMs into musculoskeletal research and clinical practice to facilitate shared decision‐making and individualised rehabilitation planning (Huang et al. 2025; Jagadish and Hadya Ananda 2025; Ochsenkuehn et al. 2022).

### 4.5 Evolution of Measurement Instruments: From Simple Scales to Multidimensional Assessment

Beyond the diversification of outcome domains, our review also demonstrates a parallel evolution in the instruments used to measure those outcomes. Pain intensity remained predominantly assessed using the Visual Analog Scale (VAS) during the earliest decades, followed by the progressive adoption of the Numeric Rating Scale (NRS), which has become the preferred instrument in contemporary clinical trials because of its simplicity, responsiveness, and ease of interpretation. Similarly, disability assessment evolved towards widespread use of standardized questionnaires such as the Roland–Morris Disability Questionnaire and the Oswestry Disability Index, while health‐related quality of life became increasingly evaluated using validated multidimensional instruments including the SF‐36, EQ‐5D, and, more recently, PROMIS (Huang et al. 2025; Jagadish and Hadya Ananda 2025; Ochsenkuehn et al. 2022).

Psychological assessment likewise evolved from isolated measures of depression towards comprehensive batteries evaluating fear‐avoidance beliefs, pain catastrophizing, kinesiophobia, anxiety, and emotional distress. Collectively, these findings indicate that the evolution of outcome assessment involved not only the inclusion of new outcome domains but also continuous refinement of the instruments used to measure them, reflecting increasing methodological rigour in CLBP research (Huang et al. 2025; Uzawa and Davis 2018).

Recent evidence further supports the methodological development of objective functional performance measures in people with chronic low back pain. Özüdoğru et al. evaluated the Five Times Sit‐to‐Stand Test in individuals with non‐specific chronic low back pain and reported excellent test–retest reliability (ICC = 0.99), supporting its use as a reproducible measure of functional performance in this population. The study also found significant associations with balance, quadriceps muscle strength, disability, and pain, highlighting the potential of functional performance tests to complement patient‐reported measures (Özüdoğru et al. 2023).

Another noteworthy finding was the gradual incorporation of objective technologies into rehabilitation research. Earlier clinical trials relied predominantly on clinician‐based examinations and self‐reported questionnaires, whereas contemporary studies increasingly integrate wearable sensors, accelerometers, digital activity monitoring, instrumented functional tests, and performance‐based assessments. These technologies complement traditional PROMs by providing objective information regarding movement behaviour, physical activity, gait characteristics, and functional performance in real‐world settings.

Although these approaches remain relatively underrepresented compared with conventional questionnaires, their emergence suggests that future clinical trials may increasingly combine subjective and objective assessments to obtain a more comprehensive understanding of patient recovery. Such integration aligns with current trends towards digital health, remote monitoring, and precision rehabilitation, in which continuous measurement of functional performance may improve both clinical decision‐making and the evaluation of intervention effectiveness (Diniz et al. 2025; Lee et al. 2025; Todorović et al. 2026).

### 4.6 Clinical Implications for Future Clinical Trials

One of the principal clinical implications of the present review is that outcome selection in CLBP clinical trials should continue to evolve beyond the assessment of isolated symptoms towards comprehensive evaluation frameworks that capture the multidimensional nature of persistent pain. Although pain intensity remains an indispensable outcome because it directly reflects one of the primary reasons patients seek healthcare, our findings clearly demonstrate that pain alone is insufficient to characterise treatment effectiveness. Contemporary rehabilitation interventions aim not only to reduce pain but also to restore physical function, improve participation, increase self‐efficacy, optimise quality of life, and facilitate long‐term self‐management.

Consequently, selecting outcome measures that simultaneously capture these dimensions is essential for accurately evaluating intervention effectiveness and for translating research findings into clinical practice. This perspective is fully consistent with current international clinical practice guidelines, which emphasise that successful management of CLBP should prioritise improvements in function and participation rather than complete elimination of pain (Ochsenkuehn et al. 2022; Thiveos et al. 2024).

Our findings also reinforce the importance of adopting standardized multidimensional outcome sets across future clinical trials. The substantial heterogeneity identified in outcome selection throughout the literature likely contributes to the persistent difficulties encountered when comparing interventions across systematic reviews and meta‐analyses. While diversity in outcome assessment reflects the complexity of CLBP, excessive variability in the domains and instruments selected may reduce comparability between studies and increase research waste. Therefore, future clinical trials should balance comprehensive multidimensional assessment with greater methodological standardisation, particularly through the routine inclusion of internationally recommended core outcome domains. Such an approach would facilitate evidence synthesis while preserving the ability to investigate emerging outcomes that may further improve the understanding of patient recovery (Chiarotto et al. 2014).

### 4.7 Implications for Research and Future Directions

The historical evolution identified in this review suggests that outcome assessment in CLBP research remains an ongoing process rather than a completed transition. Although contemporary clinical trials routinely evaluate pain, disability, physical function, and psychological health, several potentially important domains remain comparatively underrepresented. Sleep quality, fatigue, resilience, healthcare utilization, social participation, physical activity behaviour, and patient satisfaction emerged primarily during the most recent decade, indicating that these constructs have not yet achieved the same level of methodological maturity as traditional outcomes. Considering the increasing recognition that CLBP affects multiple aspects of health and daily functioning, future investigations should determine whether these emerging domains provide additional prognostic or therapeutic information beyond established measures. Such evidence will be fundamental for refining future Core Outcome Sets and ensuring that outcome selection continues to evolve alongside advances in pain science (Chiarotto et al. 2015, 2014).

Another important implication concerns the future integration of digital health technologies into outcome assessment. Our review demonstrates the early incorporation of wearable devices, accelerometers, and objective performance measures within recent clinical trials. These technologies have the potential to overcome several limitations associated with traditional self‐reported questionnaires by providing continuous, ecologically valid measurements of movement behaviour, physical activity, gait characteristics, and functional performance in real‐world environments. Rather than replacing patient‐reported outcomes, objective digital measures should be viewed as complementary sources of information capable of improving the precision, responsiveness, and ecological validity of rehabilitation research. As wearable technologies, remote monitoring systems, and artificial intelligence continue to evolve, future clinical trials may increasingly combine subjective and objective assessments to generate more individualised and clinically meaningful evaluations of treatment response (Pontes‐Silva et al. 2025).

### 4.8 Strengths and Limitations

This review has several important strengths. To our knowledge, it represents the first scoping review to comprehensively investigate the historical evolution of outcome assessment in CLBP clinical trials over a 50‐year period. By synthesising evidence from 708 clinical trials, this review provides a broad overview of how research priorities, assessment strategies, and measurement instruments have evolved alongside advances in pain science and rehabilitation. Furthermore, the use of the Joanna Briggs Institute methodology and adherence to the PRISMA‐ScR reporting guidelines enhance the methodological transparency and reproducibility of the review. An additional strength is the conceptual synthesis adopted throughout the results, which allowed the identification of temporal patterns that extend beyond simple frequency counts, providing a broader understanding of how outcome assessment has matured over successive decades.

Nevertheless, several limitations should be acknowledged. First, only clinical trials published in English were included, potentially excluding relevant evidence published in other languages. Second, because the objective of a scoping review is to map the available evidence rather than critically appraise methodological quality or estimate treatment effects, no formal risk‐of‐bias assessment was performed. Third, although outcome domains were categorised through an iterative synthesis process, some overlap between related constructs (e.g., physical function, physical performance, and disability) is inherently unavoidable because terminology has evolved considerably over the past 5 decades. Finally, the rapid expansion of digital technologies and patient‐centred outcome measures observed during the most recent years suggests that outcome assessment will likely continue to evolve beyond the period covered by the present review, requiring future updates as the field progresses.

## 5 Conclusion

This scoping review demonstrates that outcome assessment in clinical trials involving patients with CLBP has undergone a profound transformation over the past 5 decades. While pain intensity remained the only outcome consistently reported throughout the entire study period, the assessment framework progressively expanded to incorporate disability, physical function, physical performance, psychological health, quality of life, work participation, sleep, fatigue, patient satisfaction, and other patient‐centred outcomes. These findings reflect the evolution of CLBP research from a predominantly biomedical perspective towards a comprehensive biopsychosocial framework that more accurately captures the multidimensional nature of the condition. The parallel evolution of measurement instruments further illustrates increasing methodological rigour and standardisation in outcome assessment. Collectively, these findings provide a historical overview of how research priorities have changed over time and support the continued adoption of multidimensional, standardized, and patient‐centred outcome frameworks to improve the design, comparability, and clinical relevance of future clinical trials.

## Author Contributions

**Daniel Santos Rocha:** data curation, formal analysis, investigation, methodology, validation, visualisation, writing – original draft, writing – review and editing. **Alice Marques Moreira Lima:** data curation, formal analysis, investigation, methodology, validation, visualisation, writing – original draft, writing – review and editing. **Ana Clara Chung‐Cardoso:** data curation, formal analysis, investigation, methodology, validation, visualisation, writing – original draft, writing – review and editing. **Ana Paula Brito‐de‐Sousa:** data curation, formal analysis, investigation, methodology, validation, visualisation, writing – original draft, writing – review and editing. **Anna Lívia Serejo‐da‐Silva:** data curation, formal analysis, investigation, methodology, validation, visualisation, writing – original draft, writing – review and editing. **Marcelo Souza de Andrade:** data curation, formal analysis, investigation, methodology, validation, visualisation, writing – original draft, writing – review and editing. **André Pontes‐Silva:** conceptualisation, data curation, formal analysis, investigation, methodology, validation, visualisation, writing – original draft, writing – review and editing.

## Funding

The Article Processing Charge (APC) for the publication of this research was funded by the Coordenação de Aperfeiçoamento de Pessoal de Nível Superior – Brasil (CAPES) (ROR identifier: 00x0ma614). This study was funded by the CAPES (code 001). The funding source had no role in the study design, collection, analysis, interpretation of data, writing of the report, or in the decision to submit the article for publication.

## Ethics Statement

The authors have nothing to report.

## Consent

The authors have nothing to report.

## Conflicts of Interest

The authors declare no conflicts of interest.

## Supporting information


Supporting Information S1

## Data Availability

The data that support the findings of this study are available on request from the corresponding author. The data are not publicly available due to privacy or ethical restrictions.
