# Supplementary material for: Evolution of Outcome Assessment in Chronic Low Back Pain Clinical Trials Throughout the 20th and 21st Centuries: A Scoping Review
Source: Musculoskeletal Care. 2026 Oct 12;24(4):e70270. doi: 10.1002/msc.70270 (PMC13655952; doi:10.1002/msc.70270)
Supplement: Supplementary file 1 [file MSC-24-e70270-s001.pdf]

Evidence Synthesis.

REFERENCE	YEAR	OUTCOME	TEST/TOOL
(GREENFIELD *et al.*, 1975)	1975	Intensidade da dor	Contato telefônico
(FOX; MELZACK, 1976)	1976	Intensidade da dor	Questionário de Dor da McGill
(MELZACK, R *et al.*, 1980)	1980	Intensidade da dor	Questionário de Dor (MPQ) de McGill
(TURNER, 1982)	1982	Depressão	SIP e BDI
(MCCAULEY *et al.*, 1983)	1983	Intensidade da dor	Questionário de Dor da McGill
(LEHMANN, T R; RUSSELL; SPRATT, 1983)	1983	Intensidade da dor	EVA
(MELZACK, RONALD; VETERE; FINCH, 1983)	1983	Intensidade da dor	Questionário de Dor da McGill
(NOUWEN, 1983)	1983	Ativação muscular	EMG
(MACDONALD *et al.*, 1983)	1983	Intensidade da dor	VAS
(GODFREY; MORGAN; SCHATZKER, 1984)	1984	Intensidade da dor	N.A
(NWUGA; NWUGA, 1985)	1985	Mobilidade	N.A
(ZUCCO; ORLANDI, 1985)	1985	Intensidade da dor	EVA e escala semântica
(GILBERT *et al.*, 1985)	1985	Flexibilidade, Intensidade da dor	EPE, Teste de flexão lombar, AVDs
(MARTIN *et al.*, 1986)	1986	Mobilidade da coluna	N.A
(STUCKEY; JACOBS; GOLDFARB, 1986)	1986	Atividade muscular	EMG
(MOFFETT, J A KLABER *et al.*, 1986)	1986	Intensidade da dor	N.A
(MEADE *et al.*, 1986)	1986	Adesão	Autoavaliação
(LEHMANN, THOMAS R *et al.*, 1986)	1986	Intensidade da dor	EVA
(TURNER; CLANCY, 1986)	1986	Intensidade da dor	Coping Strategy Questionnaire (CSQ)
(GINSBERG; FAMAEY, 1987)	1987	Intensidade da dor	N.A
(ONGLEY *et al.*, 1987)	1987	Incapacidade, Intensidade da dor	EVA, diagrama de dor
(OVERMAN *et al.*, 1988)	1988	Evolução da dor	N.A
(MATHEWS; MORKEL; MATHEWS, 1988)	1988	Intensidade da dor	EPE
(TURNER; CLANCY, 1988)	1988	Incapacidade	N.A
(REUST; CHANTRAINE; VISCHER, 1988)	1988	Tração para dor	N.A
(JONES; JONES; KATZ, 1988)	1988	Adesão ao tratamento	Registro de agendamento
(LINTON *et al.*, 1989)	1989	Intensidade da dor	N.A

(HÄRKÄPÄÄ *et al.*, 1989)	1989	Intensidade da dor	N.A
(HURRI, 1989)	1989	Intensidade e Incapacidade	N.A
(REILLY *et al.*, 1989)	1989	Aptidão aeróbica, força muscular, Intensidade da dor	EVA, teste de força
(GARVEY; MARKS; WIESEL, 1989)	1989	Intensidade da dor	N.A
(MELLIN *et al.*, 1989)	1989	Intensidade da dor DE DOR	N.A
(KEIJSERS *et al.*, 1989)	1989	Efetividade de escola de costas de Maastricht	Escola de costas de Maastricht
(HARKAPAA *et al.*, 1990)	1990	Intensidade da dor e Incapacidade	N.A
(MEADE *et al.*, 1990)	1990	Intensidade da dor e Incapacidade	ODI, Straight leg raise
(KLEIN; EEK, 1990)	1990	Intensidade da dor	EVA
(ELNAGGAR *et al.*, 1991)	1991	Intensidade da dor	Exercícios de flexão e extensão espinal
(HÄRKÄPÄÄ *et al.*, 1991)	1991	Incapacidade	GHQ-12
(NICHOLAS; WILSON; GOYEN, 1991)	1991	Incapacidade e sofrimento	N.A
(SAARIJÄRVI, SIMO, 1991)	1991	Qualidade de vida	N.A
(VALLE-JONES *et al.*, 1992)	1992	Intensidade da dor e Incapacidade	EVA
(KHALIL *et al.*, 1992)	1992	Capacidade funcional	N.A
(SAARIJÄRVI, S. *et al.*, 1992)	1992	Sofrimento psicológico e capacidade	N.A
(TALO; RYTOKOSKI; PUUKKA, 1992)	1992	Ativação funcional, tratamento dor	N.A
(ALTMAIER *et al.*, 1992)	1992	Retorno ao trabalho	N.A
(SHEKELLE *et al.*, 1992)	1992	Efetividade de manipulação espinal	N.A
(MANNICHE *et al.*, 1993)	1993	Incapacidade	N.A
(MARCHAND *et al.*, 1993)	1993	Intensidade da dor	EVA
(ROLSTED HANSEN *et al.*, 1993)	1993	Intensidade da dor	N.A
(SPRATT *et al.*, 1993)	1993	Intensidade da dor	EVA
(TURNER; JENSEN, 1993)	1993	Intensidade da dor e Incapacidade física e psicossocial	N.A
(GUNDEWALL; LILJEQVIST; HANSSON, 1993)	1993	Absenteísmo por dor	Registro de dias de ausência no trabalho
(REINECKE; HAZARD; COLEMAN, 1994)	1994	Conforto durante a posição sentada	N.A
(TIMM, 1994)	1994	Mobilidade e Incapacidade	SCHOBER E ODI
(MITCHELL; CARMEN, 1994)	1994	Retorno ao trabalho	Status ocupacional
(THOMAS; LUNDBERG, 1994)	1994	Atividade relacionado a dor	N.A

(BLOMBERG *et al.*, 1994)	1994	ADM	N.A
(DONALDSON *et al.*, 1994)	1994	Intensidade da dor e atividade muscular	N.A para dor e EMG
(TRIANO *et al.*, 1995)	1995	Intensidade da dor	N.A
(FROST, H. *et al.*, 1995)	1995	Capacidade funcional	ODI
(MEADE *et al.*, 1995)	1995	Incapacidade	ODI
(NEWTON-JOHN; SPENCE; SCHOTTE, 1995)	1995	Intensidade e Incapacidade	N.A
(BENDIX, A. F. *et al.*, 1995)	1995	Retorno ao trabalho	Status ocupacional
(FRIEDRICH, M.; CERMAK; MADERBACHER, 1996)	1996	Intensidade da dor	EVA
(LJUNGGREN *et al.*, 1997)	1997	Afastamento do trabalho	Registro laboral
(O'SULLIVAN; TWOMEY; ALLISON, 1997)	1997	Intensidade e Incapacidade	N.A.
(KELLER, S. *et al.*, 1997)	1997	Frequência de dor	N.A.
(ROSE *et al.*, 1997)	1997	Sofrimento psicológico ,intensidade da dor , função	N.A.
(SJOGREN *et al.*, 1997)	1997	Níveis de dor	N.A
(BASLER; JÄKLE; KRÖNER-HERWIG, 1997)	1997	Intensidade da dor e Incapacidade	N.A.
(VAN POPPEL *et al.*, 1998)	1998	Intensidade da dor e afastamento do trabalho	Registro e autorrelato
(SNOOK *et al.*, 1998)	1998	Intensidade da dor e Incapacidade	N.A.
(HALDORSEN *et al.*, 1998)	1998	Retorno ao trabalho e Intensidade da dor	Registro de status laboral e autorrelato para dor
(HAMPTON ATKINSON *et al.*, 1998)	1998	Intensidade da dor e Incapacidade	Escala diferencial descrita de dor e SIP
(BENDIX, ANE F. *et al.*, 1998)	1998	Situação profissional, Intensidade da dor e Incapacidade	N.A.
(FRIEDRICH, MARTIN *et al.*, 1998)	1998	Incapacidade e intensidade da dor	N.A
(STRONG, JENNY, 1998)	1998	Atitudes disfuncional relacionado a dor	IPAM
(TORSTENSEN *et al.*, 1998)	1998	Intensidade da dor	N.A
(KYLLÖNEN *et al.*, 1998)	1998	Mobilidade, Intensidade da dor e Incapacidade	MILLION, ODI
(CHERKIN *et al.*, 1998)	1998	Incapacidade	RMDQ
(BURTON *et al.*, 1999)	1999	Medo atividade física e Incapacidade	FABQ E RMDQ
(KANKAANPÄÄ *et al.*, 1999)	1999	Intensidade da dor e Incapacidade	EVA
(MOFFETT, J. K. *et al.*, 1999)	1999	Incapacidade	RMDQ e ABERDEEN
(MANNION *et al.*, 1999)	1999	Frequência da dor e medo	FABQ
(LØNN *et al.*, 1999)	1999	Recorrência de dor	Registro de recorrência

(SOUKUP *et al.*, 1999)	1999	Recorrência	Registro da recorrência
(GHONAME, EL-SAYED A. *et al.*, 1999)	1999	Intensidade da dor	EVA
(HAMZA *et al.*, 1999)	1999	Intensidade da dor	EVA
(WERNERS; PYNSENT; BULSTRODE, 1999)	1999	Intensidade da dor e Incapacidade	EVA E ODI
(CHEING; HUI-CHAN, 1999)	1999	Intensidade da dor	EVA
(GHONAME, EL SAYED A. *et al.*, 1999)	1999	Intensidade da dor	EVA
(BENDIX, TOM *et al.*, 2000)	2000	Capacidade de trabalho	Licença de afastamento
(DALICHAU; SCHEELE, 2000)	2000	Intensidade da dor	N.A
(BROCKOW *et al.*, 2001)	2001	Intensidade da dor	EVA
(MANNION *et al.*, 2001)	2001	Atividade muscular	EMG
(CHERKIN *et al.*, 2001)	2001	Intensidade da dor e Incapacidade	RMDQ
(SHERRY; KITCHENER; SMART, 2001)	2001	Intensidade da dor	EVA
(SCULCO *et al.*, 2001)	2001	Estado de humor e dor	POMS E BPI
(KENDRICK *et al.*, 2001)	2001	Incapacidade e Intensidade da dor	EVA e RMDQ
(STIER *et al.*, 2001)	2001	Estado de saúde	IRES
(GLASER *et al.*, 2001)	2001	Intensidade da dor e Incapacidade	LBPOI
(MILLER, PAUL *et al.*, 2002)	2002	Incapacidade	RMDQ
(MOSELEY, 2002)	2002	Incapacidade	RMDQ
(LEIBING *et al.*, 2002)	2002	Intensidade da dor e Incapacidade	N.A
(HERTZMAN-MILLER *et al.*, 2002)	2002	Incapacidade	RMDQ
(GMÜNDER; KISSLING, 2002)	2002	Incapacidade e Intensidade da dor	EVA EODI
(KOVACS *et al.*, 2002)	2002	Incapacidade e Intensidade da dor	EVA e RMDQ
(SKOUEN *et al.*, 2002)	2002	Retorno ao emprego	Registro da situação de trabalho
(TSUKAYAMA *et al.*, 2002)	2002	Intensidade da dor	EVA
(HUBLEY-KOZEY; VEZINA, 2002)	2002	Atividade muscular	RMS
(MOLSBERGER *et al.*, 2002)	2002	Intensidade da dor	EVA
(HSIEH, RU LAN; LEE, 2002)	2002	Sensibilidade, intensidade da dor	PPT, EVA, Body Surface Score
(BRUEHL *et al.*, 2002)	2002	Intensidade da dor	Pain rating
(DESCARREAUX *et al.*, 2002)	2002	Intensidade da dor	EVA
(HURWITZ *et al.*, 2002)	2002	Incapacidade	RMDQ

(AKSOY; KARAN; DIRAÇOĞLU, 2002)	2002	Intensidade da dor e Incapacidade	EVA E RMDQ
(KULLICH *et al.*, 2003)	2003	Intensidade da dor, dor a pressão e Incapacidade	EVA, druckschmerz e RMDQ
(GRAY *et al.*, 2003)	2003	Custo do exame para dor lombar	Activity-Based Costing
(CHIRADEJNANT *et al.*, 2003)	2003	Intensidade da dor	N.A
(AURE; HOEL NILSEN; VASSELJEN, 2003)	2003	Incapacidade e Intensidade da dor	ODI
(KLADNY; FISCHER; HAASE, 2003)	2003	Incapacidade e capacidade funcional	ODI e HANNOVER FFbH-R
(RASMUSSEN-BARR; NILSSON-WIKMAR; ARVIDSSON, 2003)	2003	Incapacidade	ODI
(LICCIARDONE *et al.*, 2003)	2003	Qualidade de vida, Incapacidade	SF-36, RMDQ
(WEINER *et al.*, 2003)	2003	Intensidade da dor	N.A
(KERR; WALSH; BAXTER, 2003)	2003	Intensidade da dor e qualidade de vida	MPQ, EVA E SF-36
(KOVACS *et al.*, 2003)	2003	Intensidade da dor DE DOR	N.A
(SCHENK; JOZEFCZYK; KOPF, 2003)	2003	Intensidade da dor e Incapacidade	VRS,ODI
(MOLDE HAGEN; GRASDAL; ERIKSEN, 2003)	2003	Afastamento do trabalho	Registro de afastamento/licença médica
(TSUI; CHEING, 2004)	2004	Intensidade da dor da dor, elevação de perna e Incapacidade	NPRS,SLR E RMDQ
(ITOH, KAZUNORI; KATSUMI; KITAKOJI, 2004)	2004	Intensidade da dor	EVA e RMDQ
(SATOR-KATZENSCHLAGER *et al.*, 2004)	2004	Intensidade da dor	EVA E
(HELMHOUT, PIETER H. *et al.*, 2004)	2004	Incapacidade e Intensidade da dor	EVA e RMDQ
(KELLER, ANNE *et al.*, 2004)	2004	Incapacidade	Teste de Biering-Sørensen
(OH, WAN SOO; SHIM, 2004)	2004	Intensidade da dor	EVA
(PALLAY *et al.*, 2004)	2004	Incapacidade	RMDQ
(YELLAND *et al.*, 2004)	2004	Intensidade da dor	EVA
(FROST, HELEN *et al.*, 2004)	2004	Incapacidade e Intensidade da dor	ODI e RMDQ
(YIP; TSE, 2004)	2004	Intensidade da dor	EVA
(LONG; DONELSON; FUNG, 2004)	2004	Intensidade da dor	N.A
(SPINHOVEN *et al.*, 2004)	2004	Níveis de depressão	N.A
(HSIEH, LISA LI CHEN *et al.*, 2004)	2004	Intensidade da dor	F-MPQ e SF-PQ
(MAYER, TOM G. *et al.*, 2004)	2004	ADM	ADM
(COATS *et al.*, 2004)	2004	Intensidade da dor e Incapacidade	EVA E RMDQ

(HELMHOUT, P. H. *et al.*, 2004)	2004	Incapacidade e qualidade de vida	RMDQ e SF-36
(JOUSSET *et al.*, 2004)	2004	Afastamento do trabalho	N.A
(WARKE *et al.*, 2004)	2004	Intensidade da dor	Questionário de Dor da McGill
(YOZBATIRAN; YILDIRIM; PARLAK, 2004)	2004	Incapacidade e Intensidade da dor	Tteste de Biering-Sørensen e ODI
(HICKS *et al.*, 2005)	2005	Intensidade da dor	EVA
(DESCARREAUX; BLOUIN; TEASDALE, 2005)	2005	Intensidade da dor e Incapacidade	N.A
(KOMINSKI *et al.*, 2005)	2005	Qualidade de vida	SF-36
(SHABAT *et al.*, 2005)	2005	Intensidade da dor	Questionário Million
(GUÉTIN *et al.*, 2005)	2005	Intensidade da dor da dor, Incapacidade e depressão	EVA,ODI E HAD
(KOUMANTAKIS; WATSON; OLDHAM, 2005)	2005	Fadiga	MF
(FRITZ; WHITMAN; CHILDS, 2005)	2005	Incapacidade	ODI
(MILLER, ERIC R. *et al.*, 2005)	2005	Estado funcional	Questionário de Estado Funcional e SF-MPQ
(LEWIS *et al.*, 2005)	2005	Incapacidade	QUEBEC
(HLOBIL *et al.*, 2005)	2005	Tempo retorno ao trabalho	Registro ADMinistrativo
(HOEIJENBOS *et al.*, 2005)	2005	Qualidade de vida	EQ-5D
(BEKKERING *et al.*, 2005)	2005	Incapacidade	QBPDS
(ZERBINI *et al.*, 2005)	2005	Intensidade da dor e Incapacidade	LBP-IS E RMDQ
(DEFRIN *et al.*, 2005)	2005	Intensidade da dor e Incapacidade	EVA e RMDQ
(HAAS *et al.*, 2005)	2005	Incapacidade e Intensidade da dor	N.A
(HARE-MORTENSEN; LAURIDSEN; GRUNNET-NILSSON, 2006)	2006	Incapacidade	RMDQ
(ITOH, KAZUNORI *et al.*, 2006)	2006	Intensidade da dor e qualidade de vida	EVA e RMDQ
(VEIHELMANN *et al.*, 2006)	2006	Melhora dor	ODI
(COUDEYRE *et al.*, 2006)	2006	Intensidade da dor de dor e Incapacidade funcional	Escala de Incapacidade de Quebec, EVA, FABQ
(SCHWEIKERT *et al.*, 2006)	2006	Melhora dor	EVA, Roland-Morris e SF-36
(SMEETS; VLAEYEN; KESTER; *et al.*, 2006)	2006	Fatores cognitivos	Pain Catastrophizing Scale
(CHILDS; FLYNN; FRITZ, 2006)	2006	Incapacidade	ODI
(JENSEN *et al.*, 2006)	2006	Redução da lombalgia	N.A
(MAYER, JOHN M. *et al.*, 2006)	2006	Intensidade da dor	N.A

(RIVERO-ARIAS *et al.*, 2006)	2006	Incapacidade	ODI
(SJÖGREN *et al.*, 2006)	2006	Intensidade da dor	Borg CR10
(HSIEH, LISA LI CHEN *et al.*, 2006)	2006	Incapacidade e intensidade	RMDQ E EVA
(SMEETS; VLAEYEN; HIDDING; *et al.*, 2006)	2006	Incapacidade, intensidade da dor	RDQ E EVA
(STRONG, LARKIN L. *et al.*, 2006)	2006	Números de dias com dor	AUTORRELATO
(WEBSTER *et al.*, 2006)	2006	Intensidade e dependência	BOWS E ODI
(KAINZ *et al.*, 2006)	2006	Intensidade e mobilidade no dia a dia	NÃO ESPECIFICA
(WITT *et al.*, 2006)	2006	Função da coluna	HFAQ
(WÄLTI; KOOL; LUOMAJOKI, 2015)	2015	Predição de resposta ao tratamento	Terapia McKenzie e manipulação espinal
(BLÖDT *et al.*, 2015)	2015	Intensidade da dor; Incapacidade; Qualidade de vida	Escala Visual Analógica (VAS) 0-100 mm; Questionário de Deficiência Roland Morris (RDQ); Short Form-36 (SF-36)
(HÜGLI *et al.*, 2015)	2015	Adesão aos exercícios domiciliares	Autorrelato
(JEONG *et al.*, 2015)	2015	Incapacidade; Intensidade da dor	Oswestry Disability Index (ODI); Escala Numérica de Dor (NRS 0-10)
(SCHNEIDER *et al.*, 2015)	2015	Comparação de métodos de manipulação espinal vs cuidado médico usual	Manipulação espinal e cuidado médico usual
(YANG, JINMO; SEO, 2015)	2015	Equilíbrio estático (fall index); Curvatura da coluna; Intensidade da dor; Incapacidade	Balança estabilométrica (fall index); Escala Visual Analógica (EVA)
(SHAMSI, MOHAMMAD BAGHER; SARRAFZADEH; JAMSHIDI, 2015)	2015	Intensidade da dor; Incapacidade; Estabilidade lombopélvica	Escala Visual Analógica (VAS); Oswestry Disability Index (ODI); Análise de vídeo com pontuação por três fisioterapeutas
(SHAH; SALLER, 2016)	2016	Efetividade de ablação de nervo basivertebral intraóssea	Ablação de nervo basivertebral intraóssea
(SANER *et al.*, 2015)	2016	Dor e incapacidade	EVA e Roland -Morris
(USHINOHAMA *et al.*, 2016)	2016	Intensidade, dor,velocidade de oscilação	Roland-Morris, plataforma de força Kistler
(CRUZ-DÍAZ *et al.*, 2016)	2016	Dor, incapacidade	EVA e Oswestry
(JACOBS *et al.*, 2016)	2016	Intensidade da dor; Incapacidade;	Escala Numérica de Dor (NPRS); Índice de Incapacidade Oswestry (ODI); Eletromiografia de superfície (EMG) dos músculos reto abdominal (AR), oblíquo interno (IO) e oblíquo externo (EO)
(SOARES *et al.*, 2016)	2016	Intensidade da dor; Incapacidade;	CR10 DE BORG, LABIFIE(ECF)
(XIA *et al.*, 2016)	2016	Intensidade da dor; Incapacidade;	ROLAND MORRIS, QUEBEC
(GOERTZ *et al.*, 2016)	2016	Intensidade da dor; Incapacidade;	NUMERIC PAIN E QUEBEC

(ELLIOTT *et al.*, 2016)	2016	Intensidade da dor; Incapacidade;	Escala Numérica de Avaliação da Dor (NPRS); Índice de Incapacidade Oswestry (ODI); Testes de resistência muscular (flexão isométrica do tronco, prancha lateral, teste de Sorensen)
(HEDLUND *et al.*, 2016)	2016	Avaliação Global (GA - desfecho primário); Incapacidade (ODI); Intensidade da dor (VAS costas/pernas); Depressão; Status de trabalho; Medicação para dor; Frequência da dor	Avaliação Global (GA) – medida principal; Índice de Incapacidade Oswestry (ODI); Escala Visual Analógica (VAS); Escala de Depressão Zung; Questionários de status de trabalho e medicação
(KANG, JEONG-IL; JEONG; CHOI, 2016)	2016	Incapacidade; Atividade muscular	Índice de Incapacidade Oswestry (ODI); Eletromiografia (EMG)
(KAPLAN *et al.*, 2016)	2016	Intensidade da dor; Incapacidade	Escala Visual Analógica (EVA) de 10 cm; Questionário de Deficiência Roland-Morris (RMDQ)
(YU, SEONG HUN *et al.*, 2016)	2016	Intensidade da dor; Flexibilidade; Equilíbrio	Escala Visual Analógica (EVA); Teste de Schober modificado; Plataforma Tetrax (postura ereta: olhos abertos/fechados, plataforma dura/macia)
(SHAMSI, MOHAMMADBAGHER *et al.*, 2016)	2016	Intensidade da dor; Incapacidade; Espessura muscular (hipertrofia)	N.A
(STEELE *et al.*, 2016)	2016	Cinemática lombar durante a marcha	N.A
(TAMBEKAR *et al.*, 2016)	2016	Intensidade da dor; Amplitude de movimento da SLR (SROM)	Escala Visual Analógica (EVA); Goniômetro universal
(POLLY *et al.*, 2016)	2016	Intensidade da dor (SIJ); Incapacidade (ODI); Qualidade de vida	Escala Visual Analógica (EVA) de 100 pontos; Índice de Incapacidade Oswestry (ODI); EQ-5D; Short Form-36 (SF-36
(THACKERAY *et al.*, 2016)	2016	Intensidade da dor; Incapacidade	N.A
(PARK, KYUE-NAM *et al.*, 2016)	2016	Dor lombar	Escala visual analógica
(SCHALLER *et al.*, 2016)	2016	Reabilitação	N.A
(GHADYANI *et al.*, 2016)	2016	Incapacidade	Questionário de incapacidade de Roland-Morris
(MAHMOUDZADEH *et al.*, 2016)	2016	Incapacidade	Índice de Incapacidade de Oswestry
(GUASTALA *et al.*, 2016)	2016	Intensidade da dor; Incapacidade;	Escala Visual Analógica (EVA); Questionário Roland-Morris; Teste de sentar e alcançar (flexibilidade);
(GARCIA, ALESSANDRA NARCISO *et al.*, 2016)	2016	Intensidade da dor; Incapacidade	N.A

(ALI; SETHI; NOOHU, 2019)	2016	Intensidade da dor (NPRS); Incapacidade (ODI); Amplitude de movimento lombar (flexão/extensão); Atividade muscular (eretor da espinha)	Escala Numérica de Avaliação da Dor (NPRS); Questionário de Dor Lombar de Oswestry (ODI); Goniometria/inclinometria (ROM); Eletromiografia (EMG) - atividade do eretor da espinha em L3
(FIELD *et al.*, 2007)	2016	Intensidade da dor; Depressão; Ansiedade; Distúrbios do sono; Amplitude de movimento do tronco (flexão); Absenteísmo no trabalho; Produtividade no trabalho	N.A
(LICCIARDONE; GATCHEL; ARYAL, 2016a)	2016	Intensidade da dor	EVA
(LICCIARDONE; GATCHEL; ARYAL, 2016b)	2016	Intensidade da dor	EVA
(THOMAS *et al.*, 2016)	2016	Amplitude de movimento	N.A
(WAQQAR; SHAKIL-UR-REHAMAN; AHMAD, 2016)	2016	Intensidade da dor	EVA
(LEE, JUNG-SEOK; KANG, 2016)	2016	Intensidade da dor	EVA
(SOUNDARARAJAN; THANKAPPAN, 2016)	2016	Intensidade da dor	EVA
(BRANCHINI *et al.*, 2016)	2016	Intensidade da dor (VAS, BPI); Incapacidade (RMDQ); Estado de bem-estar (SF-36); Diferença clínica importante média (MCID)	Escala Visual Analógica (VAS); Inventário Breve de Dor (BPI); Questionário de Deficiência Roland-Morris (RMDQ); Pesquisa de Saúde 36 (SF-36)
(PATTI *et al.*, 2016)	2016	Intensidade da dor / Incapacidade	Índice de Incapacidade de Oswestry (ODI); Plataforma de força/posturografia
(AL-SHAREEF; OMAR; IBRAHIM, 2016)	2016	Redução de dor e incapacidade funcional	Índice de Incapacidade de Oswestry (ODI); Plataforma de força/posturografia
(CHOI *et al.*, 2016)	2016	Intensidade da dor; Força muscular das costas	Escala Visual Analógica (EVA); Dinamômetro de Músculo Lombar TKK-5402
(CHALÃ©AT-VALAYER *et al.*, 2016)	2016	Recorrência de LBP com licença médica (desfecho primário); Crenças de evitação do medo para atividade física (FABQ-P)	Recorrência de LBP com licença médica (desfecho primário); Crenças de evitação do medo para atividade física (FABQ-P)
(CLOSE *et al.*, 2016)	2016	Intensidade da dor; Frequência da dor	Escala Visual Analógica (EVA/VAS)
(MASSÉ-ALARIE *et al.*, 2016)	2016	Intensidade da dor; Incapacidade; Cinesiofobia	Questionários padronizados (N.As); Estimulação magnética transcraniana
(MICHALSEN *et al.*, 2016)	2016	Intensidade da dor	N.A
(HALL *et al.*, 2016)	2016	Intensidade da dor; Incômodo da dor; Incapacidade; Catastrofização da dor	Escala Numérica de Avaliação (NRS) 0-10; Questionário de Deficiência Roland Morris (RMDQ); Questionário de Estratégias de Enfrentamento da Dor (subescala de catastrofização)

(TEUT *et al.*, 2016)	2016	Intensidade da dor;	Índice de Avaliação Funcional (item de intensidade da dor)
(JEONG *et al.*, 2016)	2016	Qualidade de vida; intensidade da dor	N.A
(MORRIS; BONNEFIN; DARVILLE, 2015)	2016	Função específica do paciente (PSFS - desfecho primário); Intensidade da dor; Incapacidade	Escala de Função Específica do Paciente (PSFS); Escala de Dor Crônica Graduada (GCPS); Questionário de Deficiência Roland Morris (RMDQ)
(TAVARES *et al.*, 2017)	2017	Dor e Incapacidade	Pain Numeric Scale, Oswestry Disability Index, Catastrophic Thoughts Scale
(PAOLUCCI *et al.*, 2017)	2017	Intensidade da dor	EVA, McGill, Waddel,MAIA e Short Form-36 Health Survey
(FERREIRA *et al.*, 2017)	2017	Intensidade da dor	EVA,QRM
(HASANPOUR-DEHKORDI; DEHGHANI; SOLATI, 2017)	2017	Intensidade da dor; Saúde geral	Questionário de Dor da McGill; Questionário de Saúde Geral 28 (GHQ-28)
(KAEDING *et al.*, 2017)	2017	Intensidade da dor; Incapacidade; Qualidade de vida; Atividade; Capacidade muscular (flexão do tronco); Licença médica	Questionário de Roland-Morris (RMQ); Índice de Incapacidade Oswestry (ODI); Short Form-36 (SF-36); Questionário de Atividades de Freiburger; Teste isocinético da musculatura do tronco
(LOUW *et al.*, 2017)	2017	Intensidade da dor; Incapacidade	N.A
(LIU, LIZHOU *et al.*, 2017)	2017	Intensidade da dor; Incapacidade	N.A
(HUSSIEN *et al.*, 2017)	2017	Intensidade da dor; Incapacidade;	Escala Visual Analógica (EVA); Índice de Incapacidade Oswestry; Dinamômetro isocinético
(BHADAURIA; GURUDUT, 2017)	2017	Intensidade da dor; Incapacidade;	Escala Visual Analógica (EVA); Índice de Incapacidade Oswestry (ODI); Espirometria (Volume Expiratório Forçado no primeiro segundo - FEV1)
(BACKHAUSEN *et al.*, 2017)	2017	Intensidade da dor; Incapacidade;	Escala de avaliação da dor lombar (não especificada); Questionário de Deficiência Roland Morris; EQ-5D; EQ-VAS
(MARINI *et al.*, 2017)	2017	Prevalência de dor lombar; Intensidade da dor; Duração da dor	Questionário de dor auto-ADMinistrado (estruturado especificamente para o estudo)
(LETAFATKAR *et al.*, 2017)	2017	Intensidade da dor; Incapacidade; Sistema proprioceptivo; Controle do movimento lombar; Qualidade de vida; Coordenação neuromuscular	Escala Visual Analógica (EVA); Questionário de Deficiência Roland-Morris (RDQ); Testes da bateria de controle de movimento; Opção de teste da máquina HUBER; Goniômetro
(METİN ÖKMEN *et al.*, 2017)	2017	Intensidade da dor; Incapacidade;	Escala Visual Analógica (VAS); Índice de Incapacidade Oswestry modificado (mODI);

			Teste de distância ponta dos dedos-chão (FFD); Perfil de Saúde de Nottingham (NHP)
(HAHNE *et al.*, 2017)	2017	Análise de preditores de resposta a fisioterapia individualizada	Fisioterapia individualizada
(MANSELL *et al.*, 2017)	2017	Intensidade da dor; Incapacidade	Escala Numérica de Avaliação (NPRS) 0-10; Índice de Incapacidade de Oswestry
(ŞAHIN, 2017)	2017	Intensidade da dor; Incapacidade; Amplitude de movimento lombar; Força do core	Escala Visual Analógica (EVA); Questionário de Deficiência Oswestry modificado; Teste de Schober modificado; Biofeedback de pressão
(ARGUISUELAS *et al.*, 2017)	2017	Dor	COPE
(GHADYANI *et al.*, 2017)	2017	Incapacidade	Índice de Incapacidade de Oswestry
(ARAMPATZIS *et al.*, 2017)	2017	Intensidade da dor; Incapacidade; Crenças de evitação do medo	Short Form McGill Pain Questionnaire (SF-MPQ); Escala Visual Analógica (EVA); Questionário Roland Morris; Questionário de Crenças de Medo-Evitação (FABQ)
(HEAPY *et al.*, 2017)	2017	Prevenção	Questionário de Comportamentos Preventivos
(AZADINIA *et al.*, 2019)	2017	Dor lombar	Escala visual analógica
(KHOLOOSY *et al.*, 2020)	2017	Efetividade de auto-manejo baseado em resposta de voz interativa	Sistema de resposta de voz interativa para auto-manejo
(PAUNGMALI *et al.*, 2017)	2017	Controle postural (estrutura temporal das oscilações posturais)	Plataforma de força (avaliação do centro de pressão - COP) em 3 condições: olhos abertos/superfície rígida, olhos fechados/superfície rígida, olhos fechados/superfície de espuma
(AKHTAR, MUHAMMAD WASEEM; KARIMI; GILLANI, 2017)	2017	Efetividade de laser de baixa potência	N.A
(AREEUDOMWONG *et al.*, 2017)	2017	Limiar de dor à pressão	Algometria / Pressure Pain Threshold (PPT)
(KUMAR *et al.*, 2017)	2017	Intensidade da dor	EVA
(KIM, BYUNGHO J. *et al.*, 2017)	2017	Intensidade da dor	N.A
(KOKIC *et al.*, 2017)	2017	Intensidade de dor	N.A
(ANDERSEN *et al.*, 2017)	2017	Intensidade da dor; Incapacidade; Incômodo relacionado à dor; Qualidade de vida; Desfechos psicológicos	Escala Visual Analógica (EVA) de 100 mm; Questionário de Deficiência Roland; Questionário de Habilidade Funcional de Hanover; Short Form-36 (SF-36)
(MASSÉ-ALARIE *et al.*, 2017)	2017	Ntensidade da dor; Incapacidade;	N.A
(VALENZA *et al.*, 2017)	2017	Intensidade da dor; Incapacidade	Escala Numérica de Avaliação (NRS); Questionário de Deficiência Roland-Morris

			(RMDQ); Questionário da Cintura Pélvica (PGQ)
(SAPER *et al.*, 2014)	2017	Intensidade da dor; Incapacidade; Sintomas de TEPT; Medo de movimento; Catastrofização da dor	N.A
(NGUYEN *et al.*, 2017)	2017	Intensidade da dor; Função específica das costas (incapacidade)	Escala Numérica de Avaliação (NRS) 0-10; Questionário de Deficiência Roland Morris modificado
(DESAI; SRINIVASAN, 2024a)	2017	Intensidade da dor; Incapacidade; Catastrofização relacionada à dor	Escala Numérica de Dor (Numeric Pain Scale); Índice de Incapacidade Oswestry (Oswestry Disability Index); Escala de Pensamentos Catastróficos (Catastrophic Thoughts Scale)
(PETIT *et al.*, 2014)	2017	Incapacidade; Intensidade da dor; Qualidade de vida	N.A
(YILMAZ YELVAR *et al.*, 2017)	2017	Retorno ao trabalho; Intensidade da dor; Incapacidade; Qualidade de vida relacionada à saúde; Dias de licença médica	N.A
(CHAN *et al.*, 2017)	2017	Intensidade da dor; Incapacidade; Amplitude de movimento toracolombar; Equilíbrio postural dinâmico (Índices de Estabilidade Anteroposterior, Mediolateral e Geral)	McGill stabilization exerciseEscala Visual Analógica (EVA); Escala de Deficiência da Dor Lombar de Quebec; Inclinômetro; Sistema de Equilíbrio Biodex
(TÜZÜN *et al.*, 2017)	2017	Capacidade de trabalhar (desfecho primário); Qualidade de vida; Capacidade social	N.A
(SANDERS *et al.*, 2018)	2017	Intensidade	MCGILL
(KUVAČIĆ *et al.*, 2018)	2017	Incapacidade E INTENSIDADE da dor	ODI E NPRS
(ZAFEREO *et al.*, 2018)	2018	Dor e estado funcioal	EVA E Osquestry
(MINETTO *et al.*, 2018)	2018	Incapacidade	RMD
(POURAHMADI *et al.*, 2018)	2018	Incapacidade, dor, ansiedade e depressão	ODI, zung,numeric pain
(RAHBAR *et al.*, 2018)	2018	Melhora de resistência muscular abdominal com taping terapêutico	N.A
(VELASCO-ROLDÁN *et al.*, 2018)	2018	Efetividade de programa de alongamento modificado em dor menstrual	Programa de alongamento modificado
(ZARZYCKA; ZARZYCKI; KOLASA, 2018)	2018	Comparação de treinamento com fita de resistência vs exercício geral	Treinamento com fita de resistência e exercício geral
(SUNI *et al.*, 2018)	2018	Efetividade de simulador de hipoterapia	Simulador de hipoterapia
(FELDWIESER *et al.*, 2018)	2018	Efeitos imediatos e curto prazo de Kinesio Taping	N.A
(CHHABRA; SHARMA; VERMA, 2018)	2018	Comparação de Pilates seletivo vs exercícios de extensão	Pilates seletivo e exercícios de extensão
(MARTÍ-SALVADOR *et al.*, 2018)	2018	Comparação de sistemas de reabilitação	Sistemas de reabilitação

(KANG, TAE-WOO *et al.*, 2018)	2018	Intensidade da Dor	N.A
(EKLUND *et al.*, 2018)	2018	Intensidade da Dor	ODI
(KARP *et al.*, 2018)	2018	Intensidade da Dor	ODI
(RHON; MILLER; FRITZ, 2018)	2018	Intensidade da Dor	Questionário de Dor de McGill
(WILLIAMS *et al.*, 2018)	2018	Duração	Variáveis de qualidade de vida
(BARONE GIBBS *et al.*, 2018)	2018	Depressão	Questionário de Saúde do Paciente-9
(KU *et al.*, 2018)	2018	Capacidade funcional	Índice de Incapacidade de Oswestry
(CORTELL-TORMO *et al.*, 2018)	2018	Intensidade	END
(RIZZO *et al.*, 2018)	2018	Sedentarismo	Índice de Incapacidade de Oswestry
(GARAUD *et al.*, 2018)	2018	Melhora de aptidão física e qualidade de vida	Treinamento de resistência funcional
(NABAVI *et al.*, 2018)	2018	Melhora de educação sobre dor com hipnose	Hipnose com educação sobre dor
(BAE *et al.*, 2018)	2018	Melhora com fisioterapia individualizada como complemento	Fisioterapia individualizada e orientação baseada em diretrizes
(KO, KWANG-JUN *et al.*, 2018)	2018	Melhora com programa de educação terapêutica	Programa de educação terapêutica com TENS
(FINTA, REGINA; NAGY; BENDER, 2018)	2018	Intensidade da dor	N.A
(MAGALHÃES *et al.*, 2018)	2018	Intensidade da dor; Incapacidade	N.A
(KIM, HEEJAE *et al.*, 2018)	2018	Intensidade da dor	N.A
(EBADI *et al.*, 2018)	2018	Ativação muscular	Ultrassonografia
(PATIL *et al.*, 2018)	2018	Intensidade da dor	EVA
(GARCIA, ALESSANDRA NARCISO *et al.*, 2018)	2018	Comparação de atividade gradual vs fisioterapia	N.A
(WANG, SHANG-QUAN *et al.*, 2019)	2019	Melhora da dor	Questionário de acompanhamento
(WAREŃCZAK; LISIŃSKI, 2019)	2019	Dor e Incapacidade	EVA, McGill e Roland-Morris
(ZAKARI *et al.*, 2019)	2019	Comparação de terapia de liberação posicional vs exercícios de estabilização	Terapia de liberação posicional e exercícios de estabilização lombar
(TAKINACI *et al.*, 2019)	2019	Efetividade de terapia de spa (balneoterapia)	Balneoterapia
(SAJADI *et al.*, 2019)	2019	Dor	WBV (baixa e alta frequência)
(MAS *et al.*, 2019)	2019	Redução da incapacidade, da gravidade da dor e na melhoria da qualidade de vida	Questionários de Dor (MGPQ) e Qualidade de Vida (SF-12) da McGill.
(PETROZZI *et al.*, 2019)	2019	Eficácia de um programa psicológico oferecido pela internet (moodgym),	RMDQ e Questionário de Autoeficácia do Paciente
(SHARIAT *et al.*, 2019)	2019	Qualidade de vida	Variáveis de qualidade de vida

(NEAMAT ALLAH *et al.*, 2019)	2019	Dor	Escala Numérica de Dor
(AMPOMAH *et al.*, 2019)	2019	Tamanho muscular	Ressonância
(SHAMSI, MOHAMMADBAGHER *et al.*, 2020)	2019	Incapacidade	Índice de Incapacidade de Oswestry
(MCCARTHY; POTTER; OLDHAM, 2019)	2019	Incapacidade	Índice de Incapacidade de Oswestry
(TOELLE *et al.*, 2019)	2019	Intensidade da dor lombar	Escala numérica de 11 pontos
(SHARMA *et al.*, 2019)	2019	Qualidade de vida	Variáveis de qualidade de vida
(TAULANIEMI *et al.*, 2019)	2019	Intensidade	Escala numérica de dor
(SUH *et al.*, 2019)	2019	Intensidade	Escala visual analógica
(ELKASSABANY, 2019)	2019	Comparação de abordagem biopsicossocial com e sem terapia manual	Fisioterapia biopsicossocial e terapia manual ortopédica
(AKHTAR, M. SHAHID *et al.*, 2022)	2019	Comparação de Kinesio Taping vs fisioterapia tradicional	Kinesio Taping e fisioterapia tradicional
(KAMALI *et al.*, 2019)	2019	Dor e Incapacidade	EVA e ODI
(MANE; VARADHARAJULU; SHINDE, 2019)	2019	Intensidade da dor	ENA
(NEYAZ *et al.*, 2019)	2019	Intensidade da dor	Escala de Avaliação da Dor para Militares e Veteranos (DVPRS)
(OZSOY *et al.*, 2019)	2019	Intensidade da dor	N.A
(SAMANI *et al.*, 2019)	2019	Intensidade da dor	N.A
(SHEBIB *et al.*, 2019)	2019	Incapacidade	ODI
(SAWANT; SHINDE, 2019)	2019	Intensidade da dor	EVA
(TANTAWY *et al.*, 2019)	2019	Intensidade da dor	N.A
(CELENAY; KAYA; UCURUM, 2019)	2019	Intensidade da dor	EVA
(SEO, HYE-RAN; KIM, 2019)	2019	Melhora de atividade muscular e estabilidade lombar	Exercício Gyrotonic expansion system e estabilidade de tronco
(SUMAN *et al.*, 2019)	2019	Efetividade e custo-utilidade de estratégia ehealth multifacetada	Estratégia eHealth multifacetada
(ALRWAILY *et al.*, 2019)	2019	Combined stabilization exercises and NMES effective for pain reduction	Stabilization exercises, neuromuscular electrical stimulation (NMES)
(DARLOW *et al.*, 2019)	2019	FREE approach (fear reduction early exercise) effective for low back pain management in general practice	Fear reduction exercised early (FREE) approach
(GHOLAMI BORUJENI; YALFANI, 2019)	2019	Inspiratory muscle training reduces postural sway in athletes with chronic low back pain	Inspiratory muscle training
(LISI *et al.*, 2019)	2019	Pulsed electromagnetic field therapy reduces non-specific low back pain	Pulsed electromagnetic field (PEMF) therapy
(LARSEN *et al.*, 2019)	2019	Melhora de efetividade com tutores leigos	Escola de costas com tutores leigos

(TEYCHENNE *et al.*, 2019)	2019	Melhora de sintomas depressivos com exercício	Condicionamento geral de força vs controle motor com terapia manual
(WASEEM *et al.*, 2019)	2019	Redução de incapacidade com exercícios de estabilização central	Exercícios de estabilização central
(DIMER DA LUZ *et al.*, 2019)	2019	Melhora com estimulação neuromuscular elétrica e estabilidade central	Estimulação elétrica neuromuscular com exercícios de estabilidade central
(PHATTHARASUPHARERK *et al.*, 2019)	2019	Redução da dor em trabalhadores de escritório	Prática de Qigong
(VERBRUGGHE; AGTEN; STEVENS; HANSEN; DEMOULIN; EIJNDE; *et al.*, 2019)	2019	Intensidade do exercício	Exercício de alta intensidade
(ENDAMLI; BAYRAMLAR; TURHAN, 2019)	2019	Efetividade de tratamento fascial	Tratamento fascial
(BATISTELLA *et al.*, 2020)	2020	Intensidade da dor	EVA, PA, Oswestry Low Back Disability Index e trunk resistance [TR] test
(ALMEIDA *et al.*, 2020)	2020	Intensidade da dor	NRS e McGill
(DE OLIVEIRA *et al.*, 2020)	2020	Intensidade da dor	Numeric Pain Rating Scale
(FAGUNDES LOSS *et al.*, 2020)	2020	Intensidade da dor	Numeric Pain Rating Scale<br>Pressure Algometee,<br>Force Platform
(CARVALHO *et al.*, 2020)	2020	Intensidade da dor	EVA e roland morris
(O'KEEFFE *et al.*, 2020)	2020	Comparação de terapia cognitivo-funcional vs exercício em grupo	Terapia cognitivo-funcional e exercício com educação em grupo
(JUNG, KYOUNG-SIM; IN, 2020)	2020	Melhora de dor, amplitude de movimento e qualidade de vida com corrente interferencial	Terapia de corrente interferencial
(PETROFSKY; LAYMON; LEE, 2020)	2020	Comparação de TENS vs calor contínuo de baixo nível	TENS e calor contínuo de baixo nível
(SARACOGLU *et al.*, 2020)	2020	Qualidade de vida	N.A
(MEHYAR *et al.*, 2020)	2020	Efeito de mobilização lombar grau III em músculos de costas	Mobilização lombar grau III
(NAMBI; ABDELBASSET; ALSUBAIE; *et al.*, 2020)	2020	Efeitos radiográficos e inflamatórios de treinamento isocinético	Treinamento isocinético
(ABBASI; HADIAN RASANANI; GHOTBI; OLYAEI; BOZORGMEHR; *et al.*, 2020)	2020	Efeito de curto prazo de Kinesiology Taping	Kinesiology Taping
(TAGLIAFERRI *et al.*, 2020)	2020	Comparação de força geral vs controle motor e terapia manual	Treinamento de força geral e controle motor com terapia manual
(SHIN; LEE; YOO, 2020)	2020	Melhora de dor e incapacidade com tratamento específico por classificação	Tratamento específico por classificação
(ALTINBILEK, 2020)	2020	Intensidade da dor	escala analógica visual (VAS), a gravidade da depressão usando o Beck Depression Inventory (BDI) e a deficiência funcional usando o Oswestry Disability Index (ODI).

(WALEWICZ *et al.*, 2020)	2020	Intensidade da dor	a Escala de Dor de Laitinen (LPS), o Questionário Roland–Morris (RMQ), o Teste de Schober original (OST)
(SANEI *et al.*, 2020)	2020	Eficácia de um método de terapia manual persa	de incapacidade de Roland-Morris, as pontuações da Visual Analogue Scale escores de lum
(ABBASI; HADIAN RASANANI; GHOTBI; OLYAEI; RASOULI, 2020)	2020	Intensidade da dor	o Questionário Roland–Morris (RMQ)
(NAROUEI *et al.*, 2020)	2020	Intensidade da dor	escala analógica visual (VAS) e o Índice de Deficiência de Oswestry.
(OH, YOUN-JUNG; PARK; LEE, 2020)	2020	Intensidade da dor	escala visual quádrupla analógica (QVAS), o índice de deficiência de Oswestry - versão coreana (ODI-K), espessura e taxa de contração do diafragma, e teste de capacidade pulmonar.
(OH; PARK; MYUNG-MO, 2020)	2020	Intensidade da dor	(Visual Analog Scale), disability level (Rolland Morris Disability Questionnaire), depressive symptoms (Beck Depression Scale) were examined before and after the treatment.
(JUNG, KYOUNG-SIM *et al.*, 2020)	2020	Propriocepção na lombalgia	Escala Numérica de Avaliação da Dor (ENAD) e Inclinômetro digital duplo
(SIPAVICIENE; KLIZIENE, 2020)	2020	Incapacidade	Índice de Incapacidade de Oswestry
(KIM, BEOMRYONG; YIM, 2020)	2020	Intensidade	Escala visual analógica
(FISHER *et al.*, 2020)	2020	Medo de perturbação	Questionário de Crenças de Medo-Evitação
(FOSBERG *et al.*, 2020)	2020	Intensidade da dor	Variáveis de qualidade de vida
(WATTANANON *et al.*, 2020)	2020	Amplitude de movimento lombar	Técnica de enrijecimento
(STURIONA *et al.*, 2020)	2020	Redução de limiar de dor à pressão e alteração de amplitude EMG	Agulhamento seco profundo
(YAO *et al.*, 2020)	2020	Efetividade de Wuqinxi (exercício tradicional chinês)	N.A
(BORUJENI; YALFANI, 2020)	2020	Intensidade da dor	Acupuntura
(NAMBI; ABDELBASSET; ALQAHTANI; *et al.*, 2020)	2020	Comparação de treinamento de equilíbrio vs estabilização	Treinamento de equilíbrio e estabilização
(KHODADAD *et al.*, 2020)	2020	Intensidade da dor	EVA
(KONG, JIANG-TI *et al.*, 2020)	2020	Intensidade da dor	PROMIS
(MAYER, JOHN M. *et al.*, 2020)	2020	Tempo de afastamento do trabalho	Autorrelato de episódios de dor lombar
(NIM *et al.*, 2020)	2020	Intensidade da dor	N.A
(TAWREJ; KAUR; GHODEY, 2020)	2020	Intensidade da dor	ENA

(WEINER *et al.*, 2020)	2020	Intensidade da dor	EVA
(AZAB *et al.*, 2020)	2020	Intensidade da dor	N.A
(GROESSL *et al.*, 2020)	2020	Incapacidade	RDMQ
(PARK, SUN JA; KIM; YANG, 2020)	2020	Capacidade vital respiratória e função pulmonar	Espirometria
(FINTA, R *et al.*, 2020)	2020	Capacidade respiratória e controle motor	N.A
(SCHMIDT *et al.*, 2020)	2020	Incapacidade	ODI
(NAMBI; ABDELBASSET; ELSAYED; *et al.*, 2020)	2020	Comparação de treinamento isocinético vs realidade virtual	Treinamento isocinético e treinamento em realidade virtual
(SUN, XU *et al.*, 2020)	2020	Efetividade de agulhamento com pino de polegar em tripulação aérea	Agulhamento com pino de polegar
(KIM, SOOCHUL; JEE, 2020)	2020	Intensidade da dor	Exercício em plataforma móvel 3D
(PARK, SIHWA *et al.*, 2020)	2020	Intensidade da dor	Simulador equestre
(YEH *et al.*, 2020)	2020	Efetividade de sapatos de acupuntura eletrônica	Sapatos de acupuntura eletrônica
(MOSLEMI; SAFAVI FAROKHI, 2020)	2020	Intensidade da dor	Eletroacupuntura em pontos gatilho do glúteo máximo
(ABDELBASSET *et al.*, 2020)	2020	Intensidade da dor	Laser de alta intensidade e baixa potência
(AHMADI *et al.*, 2020)	2020	Feldenkrais method similar to core stability exercise for chronic low back pain	Feldenkrais method, core stability exercise
(FINK *et al.*, 2020)	2020	Comparação de efetividade de três técnicas manuais	Terapia manual de pontos gatilho
(DILEKÇI; ÖZKUK; KAKI, 2020)	2020	Intensidade da dor	Balneoterapia
(KIM, TAEYEONG *et al.*, 2020)	2020	Intensidade da dor	Simulação de equitação
(SEO, U-HYEOK; KIM; LEE, 2020)	2020	Intensidade da dor	Mobilização de Mulligan e terapia a laser de baixa potência
(KABAŁA *et al.*, 2020)	2020	Efetividade de tecnologias modernas em reabilitação	Tecnologias modernas de reabilitação
(LAOSEE *et al.*, 2020)	2020	Comparação de massagem tailandesa vs com compressas herbais	Massagem tailandesa tradicional e com compressas herbais
(KIM, SEUNG-KOOK *et al.*, 2020)	2020	Comparação de custo-efetividade de cadeira de massagem vs fisioterapia	Terapia de cadeira de massagem e fisioterapia básica
(DAYANIR *et al.*, 2020)	2020	Redução de pontos gatilho com técnica neuromuscular integrada	Técnica integrada de inibição neuromuscular
(MCKEE *et al.*, 2020)	2020	Comparação de efetividade	Acupuntura individual e em grupo
(SRIVASTAVA *et al.*, 2020)	2020	Efetividade de técnica de energia muscular e diagnóstico mecânico	Técnica de energia muscular e terapia de diagnóstico mecânico
(VERBRUGGHE *et al.*, 2020)	2020	Efetividade de diferentes modalidades de exercício	Treinamento de alta intensidade

(OPEYEMI AYODIIPO IDOWU; ADE FATAI ADENIYI, 2020)	2020	Intensidade da dor	Atividade gradual e caminhada monitorada diariamente
(MATARÁN-PEÑARROCHA *et al.*, 2020)	2020	Comparação de efetividade supervisionada vs não supervisionada	Programa de exercício fisioterapêutico supervisionado e não supervisionado
(KİBAR *et al.*, 2020)	2020	Comparação de TENS combinado com corrente interferencial	TENS combinado com terapia de corrente interferencial
(DEPAOLI LEMOS *et al.*, 2021)	2021	Intensidade da dor	Eva,Wells Bench, Static Trunk Endurance Test, Sorensen Endurance Tes
(MIYAMOTO *et al.*, 2021)	2021	Intensidade da dor	EVA E Patient-Specific Functional Scale
(SALEMI *et al.*, 2021)	2021	Intensidade da dor	ENAD,QDM,LDP E AP
(JASSI *et al.*, 2021)	2021	Intensidade da dor	Oswestry
(OLKOSKI *et al.*, 2021)	2021	Resistencia muscular lombar, desempenho físico	(Teste de Sentar e Levantar Repetidamente Teste de Sorensen e Teste de Aptidão Física Máxima
(LEEMANS *et al.*, 2021)	2021	Intensidade da dor	pressure pain threshold e escala de dor
(MORSØ *et al.*, 2021)	2021	Intensidade da dor	RDMQ E QALYS
(HODGES *et al.*, 2021)	2021	Alfabetização em Saúde	Questionário de Alfabetização em Saúde (HLQ)
(SEMRAU *et al.*, 2021)	2021	Capacidade funcional	Questionário de Capacidade Funcional de Hannover
(KIM, YOUNG KYUN; CHO; LEE, 2021)	2021	Intensidade da dor e incapacidade	VAS, Escala de Dor Facial (EDF) e ODI
(GHOLAMI-BORUJENI; YALFANI; AHMADNEZHAD, 2021)	2021	Ativação musucular	EMG
(SENBURSA; PEKYAVAS; BALTACI, 2021)	2021	Intensidade da dor, força e incapacidade	VAS, Dinamometro e ODI
(ALIKHAJEH; BARABADI; MOHAMMAD RAHIMI, 2021)	2021	Intensidade da dor e incapacidade	N.A
(KIM, SOO KYOUNG; PARK, 2021)	2021	Intensidade da dor e incapacidade	N.A
(MOREIRA *et al.*, 2021)	2021	Força muscular, flexibilidade e sintomas de lombalgia	N.A
(SANIKA; PREM; KARVANNAN, 2021)	2021	Incapacidade	ODI
(HUSSEIN; MORSI; ABDELRAOOF, 2021)	2021	Intensidade da dor	Exercícios de Pilates clínico
(SARAÇOĞLU *et al.*, 2021)	2021	Efetividade de educação em neurofisiologia da dor pré-operatória	Educação em neurofisiologia da dor
(NAVA-BRINGAS *et al.*, 2021)	2021	Comparação de exercícios de estabilização vs flexão	Exercícios de estabilização e flexão
(RABIEI; SHEIKHI; LETAFATKAR, 2021)	2021	Comparação de fisioterapia com educação em neurociência vs fisioterapia isolada	Fisioterapia com educação em neurociência
(LANGAGERGAARD *et al.*, 2021)	2021	Intensidade da dor	Intervenção breve e multidisciplinar

(SITGES *et al.*, 2021)	2021	Efeitos agudos de intervenção de exercício breve	Intervenção de exercício breve
(SIPKO; GLIBOWSKI; KUCZYŃSKI, 2021)	2021	Intensidade da dor	N.A
(TAHERI; KHOSRAWI; RAMEZANI, 2021)	2021	Intensidade da dor	A escala visual analógica e o Índice de Incapacidade de Oswestry (ODI)
(BHAT P *et al.*, 2021)	2021	Intensidade da dor	Escala Visual Analógica (VAS), a Escala de Função Específica do Paciente (PSFS)
(AUGER *et al.*, 2021)	2021	Intensidade da dor	Visual Analog Scale (VAS),
(EBADI *et al.*, 2012)	2021	Efeito da ecografia contínua	amplitude de movimento (ROM)
(ZAWORSKI; LATOSIEWICZ, 2021)	2021	Intensidade da dor	ndice de Deficiência de Oswestry (ODI) e a Escala Funcional de Dor nas Costas (BPFS).
(RYYNÄNEN *et al.*, 2021)	2021	Eficácia do treinamento	deficiência (Índice de Deficiência Oswestry, ODI)
(NISHIMURA *et al.*, 2021)	2021	Intensidade da dor	Índice de Deficiência de Oswestry e da Escala Visual Analógica
(IN *et al.*, 2021)	2021	Intensidade da dor	Escala Visual Analógica (EVA) / Índice de Incapacidade de Oswestry
(JUNG, KYOUNG-SIM *et al.*, 2021)	2021	Equilíbrio postural na lombalgia	Escala visual analógica / Plataforma de força
(HADIQA ADNAN *et al.*, 2021)	2021	Intensidade da dor	Escala de avaliação da estabilidade do core
(KARASEL; ONCEL; SONMEZ, 2021)	2021	Humor	Inventário de Depressão de Beck
(IQRA NAYYAB *et al.*, 2021)	2021	Intensidade da dor	Avaliação da estabilidade do core
(KREKOUKIAS *et al.*, 2021)	2021	Marcha	Sistema optoeletrônico
(ANNASWAMY *et al.*, 2021)	2021	Qualidade de vida	EuroQol 5-Dimension
(SIMULA *et al.*, 2021)	2021	Função física	PROMIS PF-20
(RIZZO *et al.*, 2021)	2021	Iintensidade da dor	Escala numérica de dor
(DAGENAIS; HAYFLINGER; MAYER, 2021)	2021	Anos de vida ajustados pela qualidade	Questionário
(RHON *et al.*, 2021)	2021	Decisão inicial de utilização de serviços médicos	Aplicativo
(MACEDO *et al.*, 2021)	2021	Intensidade da dor	Escala numérica de classificação da dor
(ANAN *et al.*, 2021)	2021	Gravidade subjetiva da dor	Escala de pontuação de 1 a 5
(NIM *et al.*, 2021)	2021	Sensibilidade à dor mecânica e termica	Pressure Pain Threshold (PPT)
(BURNS *et al.*, 2021)	2021	Incapacidade	ODI
(FAZEL; ZOLAKTAF; NEZHADIAN, 2021)	2021	Intensidade da dor	EVA
(ABDELBASSET *et al.*, 2021)	2021	Intensidade da dor	N.A
(KIM; KIM; CHUNG, 2021)	2021	Intensidade da dor	ENA

(GARCIA, LAURA M *et al.*, 2021)	2021	Intensidade da dor	ENA
(MUMTAZ *et al.*, 2021)	2021	Intensidade de dor	N.A
(SATO *et al.*, 2021)	2021	Intensidade de dor	N.A
(AHMED; MAHARAJ; VAN OOSTERWIJCK, 2021)	2021	Melhora de desfechos biopsicossociais com exercícios e técnica de energia muscular	Exercícios de estabilização dinâmica e técnica de energia muscular
(ZHENG, YI-LI *et al.*, 2021)	2021	Efeitos de exercício de vibração de corpo inteiro em músculos centrais	Exercício de vibração de corpo inteiro
(DADARKHAH *et al.*, 2021)	2021	Comparação de instrução de exercício remota vs presencial	Instrução de exercício remota e presencial
(ALVANI; SHIRVANI; SHAMSODDINI, 2021)	2021	Neuromuscular exercises improve proprioception and balance in military personnel	Neuromuscular exercises, proprioception tests, balance assessment
(ANSARI; ELMIEH; ALIPOUR, 2021)	2021	Aquatic exercise improves flexibility and trunk muscle function in postmenopausal women	Aquatic exercise
(OŻÓG *et al.*, 2021)	2021	Myofascial release techniques reduce muscle activity and pain	Myofascial release techniques, EMG analysis
(ESPEJO-ANTÚNEZ *et al.*, 2021)	2021	Alterações em variabilidade da frequência cardíaca	Terapia de corrente interferencial
(LI, ZHICHENG *et al.*, 2021)	2021	Melhora de ajustes posturais antecipatórios	Treinamento em realidade virtual
(ALZAHRANI *et al.*, 2021)	2021	Efeito de briefing nas expectativas e resultados de acupuntura	Acupuntura com briefing
(KAYA; CUG; BEHM, 2021)	2021	Melhora com programa de caminhada baseado em wearables	Programa de caminhada com wearables e fisioterapia usual
(DARNALL *et al.*, 2021)	2021	Comparação de Pilates vs exercícios gerais vs educação	Pilates, exercícios gerais e workshops educacionais
(LANG *et al.*, 2021)	2021	Intensidade da dor	Habilidades de manejo da dor e terapia cognitivo-comportamental
(MANIKANDAN *et al.*, 2021)	2021	Intensidade da dor	Programa de caminhada com pedômetro
(MARÍN-MÉNDEZ *et al.*, 2021)	2021	Efetividade do tratamento robótico	Robô para tratamento
(NGUYEN *et al.*, 2021)	2021	Intensidade da dor	Tratamento manipulativo osteopático
(PAULO *et al.*, 2021)	2021	Intensidade da dor	Técnica de liberação miofascial toracolombar
(BRUEHL *et al.*, 2021)	2021	Aerobic exercise training may alter opioid analgesic responses in chronic low back pain patients	Aerobic exercise training
(GONZÁLEZ *et al.*, 2021)	2021	Validação de técnica de mobilização neural	Mobilização neural e técnica sham
(IDOWU *et al.*, 2021)	2021	Efetividade de atividade gradual e caminhada	Atividade gradual e caminhada monitorada
(JOSHI; YADAV; PAWALIA, 2021)	2021	Redução da dor lombar crônica	Técnica de inibição muscular cervical
(KAZEMI *et al.*, 2021)	2021	Efetividade de intervenções em enfermeiros	Intervenções presenciais e via mídia social

(SCHRÖDER *et al.*, 2021)	2021	Comparação de efetividade do modelo betterback	Modelo BetterBack e cuidado de rotina
(NAMBI *et al.*, 2021)	2021	Intensidade da dor	Exercícios de estabilização combinados com exercício do assoalho pélvico
(ABDEL-AZIEM *et al.*, 2021)	2021	Eficácia de correção postural integrada	Correção de postura cervical com exercícios de estabilização lombar
(ELABD *et al.*, 2021)	2021	Intensidade da dor	Realidade virtual e treinamento isocinético
(WAONGENNGARM *et al.*, 2021)	2021	Novo início de dor lombar	Avaliação autorrelatada
(GARCIA, LAURA M. *et al.*, 2022)	2022	Intensidade da dor	DVPRS E PROMIS
(NARDIN *et al.*, 2022)	2022	Intensidade da dor	oswetry e EVA
(CASTRO *et al.*, 2022)	2022	Intensidade da dor	NRS e ODI
(ITOH, NAOHIRO *et al.*, 2022)	2022	Mudanças na produtividade no trabalho, intensidade da dor, qualidade de vida, medo do movimento e depressão	N.A
(MINAKAWA *et al.*, 2022)	2022	Intensidade da dor	NRS
(SORMUNEN *et al.*, 2022)	2022	Capacidade laboral autoavaliada	Índice de capacidade para o trabalho
(SAIKLANG *et al.*, 2022)	2022	Intensidade da dor	N.A
(EZEMA *et al.*, 2022)	2022	Intensidade da dor	N.A
(LI, KEQIANG *et al.*, 2022)	2022	Diminuição de ansiedade relacionada a COVID-19 e dor lombar com Baduanjin	A Escala de Ansiedade por Coronavírus (CAS), a Escala de Bem-Estar Psicológico (PWBS) e o Questionário Nórdico de Sintomas Musculoesqueléticos (NMQ)
(ÖZDEN *et al.*, 2022)	2022	Intensidade da dor	N.A
(SARACOGLU *et al.*, 2022)	2022	Intensidade da dor	N.A
(KONG, PUI WAH *et al.*, 2022)	2022	Força isométrica, rigidez muscular , fatigabilidade e incapacidade	ODI
(SINGPHOW *et al.*, 2022)	2022	Intensidade da dor	N.A
(AFZAL *et al.*, 2022)	2022	Intensidade da dor	VAS e ODI
(DENNERLEIN *et al.*, 2022)	2022	Intensidade da dor	ODI
(DINIZ *et al.*, 2022)	2022	Crenças sobre Dor Lombar	Questionário de Crenças sobre Dor Lombar
(TAMARTASH; BAHRPEYMA; MOKHTARI DIZAJI, 2022)	2022	Intensidade da dor	N.A
(YOON *et al.*, 2022)	2022	Intensidade da dor	intensidade da dor por meio de EMGsuperficial (sEMG), escala analógica visual (VAS) e um questionário de dor de McGill em formato curto (SF-MPQ)

(ZAMANI *et al.*, 2022)	2022	Intensidade da dor	Escala numérica de avaliação de dor (NPRS)
(GARCIA, LAURA *et al.*, 2022)	2022	Intensidade da dor	PROMIS
(ZAHRA MASOOD *et al.*, 2022)	2022	Intensidade da dor	ODI e VAS
(GORJI *et al.*, 2022)	2022	Intensidade da dor	VAS e RMDQ
(SUN, MING-MEI *et al.*, 2022)	2022	Intensidade da dor	VAS, ODI e termografia infravermelha
(KIM, JI-HWAN; PARK, 2022)	2022	Intensidade da dor	Inbody, Myoton-Pro, VAS e ODI
(NAMBI *et al.*, 2022)	2022	Intensidade da dor	VAS
(LENA *et al.*, 2022)	2022	Intensidade da dor, incapacidade.	VAS, RMDQ. teste de sentar e alcançare SF12
(RAJFUR, JOANNA *et al.*, 2022)	2022	Capacidade funcional	Índice de Incapacidade de Oswestry
(RAJFUR, KATARZYNA *et al.*, 2022)	2022	Intensidade da dor	Escala de Dor de Laitinen
(KIM, JAE-HONG *et al.*, 2022)	2022	Intensidade da dor	Escala visual analógica
(BLANCHARD *et al.*, 2022)	2022	Intensidade da dor	Registro, VAS, RMDQ e Dallas Pain (DPQ)
(NAJAFI GHAGHOLESTANI *et al.*, 2022)	2022	Postur, a intensidade da dor e a incapacidade	VAS, biofeedback de pressão e ODI
(AHMADIZADEH *et al.*, 2020)	2022	Atividade abdominal transversa, dor, incapacidade e depressão	US, VAS, RMDQ e Inventário de Depressão de Beck-II
(KİANİ *et al.*, 2022)	2022	Intensidade da dor	EVA
(TORLAK *et al.*, 2022)	2022	Intensidade da dor, incapacidade peso corporal	EVA, Indice de Barthel
(VIJAYALAKSHMI *et al.*, 2022)	2022	Intensidade da dor	ENA, inclinômetr, US e ODI
(VITMAN *et al.*, 2022)	2022	Amplitude de movimento	N.A
(LEE, JUNG HWAN *et al.*, 2022)	2022	Incapacidade	ODI
(ZUREK *et al.*, 2022)	2022	Atividade bioelétrica, amplitude de movimento e intensidade da dor	Eletromiógrafo, teste de Schober e VAS
(MARUGÁN-RUBIO *et al.*, 2022)	2022	Melhora de espessura muscular, pressões respiratórias, intensidade da dor, Incapacidade, Limiar de dor à pressão e qualidade de vida	US, Manuvacuômetro, Escala de dor, algometria, RMDQ, SF-12
(ROLVING *et al.*, 2022)	2022	Incapacidade	RMDQ
(HOOKER *et al.*, 2022)	2022	Padrão de movimento durante uma atividade funcional	Teste funcional associado à análise cinemática tridimensional
(K. VISHALI; R.V. VIJAYA KUMAR; VASANTHAN, 2022)	2022	Qualidade de vida	QOL-Quality of Life
(PENG *et al.*, 2022)	2022	Incapacidade	RMDQ

(CHEN, ZHEN-HUA *et al.*, 2022)	2022	Intensidade da dor, incapacidade, flexibilidade e crenças de medo-evitação	SF-MPQ, ODI, distância dedo-chão (FFD), da distância do teste de Schober, do questionário de crenças de medo-evitação (FABQ)
(IBRAHIMI GHAVAMABADI *et al.*, 2022)	2022	Qualidade de vida, redução de dor e incapacidade	SF-36, VAS e RMDQ
(AISWARYA K RAVINDRAN *et al.*, 2022)	2022	Intensidade da dor	NPRS
(KOUTARAPU; GHUMARE, 2022)	2022	Intensidade da Dor, Incapacidade e flexibilidade	EVA, ODI e RMDQ
(ÇAKMAK; ATICI; GÜLŞEN, 2022)	2022	Intensidade, incapacidade e depressão	EVA, RMDQ E BDI
(KASIMIS *et al.*, 2023)	2023	Intensidade da dor, capacidade funcional, Limiar de Dor à Pressão e mobilidade	NRS, RMDQ, Algometria e teste de ponta dos dedos ao chão (FTF)
(CAÑA-PINO; APOLO-ARENAS; CARMONA DEL BARCO; *et al.*, 2023)	2023	Distância entre o centro de pressão e a superfície articular e o deslocamento do centro de pressão	N.A
(CAÑA-PINO; APOLO-ARENAS; FALLA; *et al.*, 2023)	2023	Intensidade da dor	NPRS
(QIMING *et al.*, 2023)	2023	Tônus e rigidez dos músculos	N.A
(LYTRAS *et al.*, 2023)	2023	Intensidade da dor, função e equilíbrio	VAS, RMDQ, TUG e TUG e Escala de Equilíbrio de Berg
(BAGHANI, 2023)	2023	Intensidade da dor, incapacidade e resistência dos músculos	Quebec, o teste de abdominais e o teste de Biering-Sorenson
(KANAAN *et al.*, 2023)	2023	Intensidade da dor, conhecimento, atitude, incapacidade, sintomas de saúde mental, Ansiedade e Estresse, e medo-evitação	VAS, Questionário de Conhecimento sobre Dor Lombar, Questionário de Atitudes em Relação à Dor Lombar, ODI, DASS-21 e Questionário de Crenças de Medo-Evitação
(ALARAB; SHAMEH; AHMAD, 2023)	2023	Intensidade da dor, incapacidade e resistência muscular	EVA,MODI e Teste de Resistência
(BATES *et al.*, 2023)	2023	Resistência	N.A
(NAMBI *et al.*, 2023)	2023	Intensidade dar dor, cinesiofobia e valores endocrinos	N.A
(BERBEROĞLU; ÜLGER, 2023)	2023	Adesão, intensidade da dor e incapacidade	EARS, VAS e ODI
(RAJAN; GURUDUT, 2023)	2023	Limiar de dor à pressão, ADM e Incapacidade	Algometro, goniometro e ODI
(PROSKURA; RUTKOWSKA-KUCHARSKA; SOBERA, 2023)	2023	Intensidade da DOR	VAS
(SONI; TOLE, 2023)	2023	Intensidade da dor	VAS e Resistência Core
(ALKHATHAMI *et al.*, 2023)	2023	Função, intensidade da dor e incapacidade	Functional Movement Screen ™ (FMS ™), NRS e MODI
(AMARAL; PÁSSARO; CASAROTTO, 2023)	2023	Intensidade da dor, depressão e ansiedade	McGill, BDI, VAS e Ansiedade

(ŞIMŞEK; YAĞCI; KORKMAZ, 2023)	2023	Intensidade da dorm ADM, Incapacidade, crenças	VAS, escala de amplitude de movimento da coluna lombar II, RMDQ, Questionário de Crença de Evitação por Medo
(AGRE; AGRAWAL, 2023)	2023	Intensidade da dor e incapacidade	VAS e ODI
(ER; YÜKSEL, 2023)	2023	Intensidade da dor	VAS
(VILLATORO-LUQUE *et al.*, 2023)	2023	Intensidade da dor, incapacidade, catastrofização e força	VAS, ODI, PCS e dinamometro
(VILLATORO-LUQUE *et al.*, 2025)	2023	Intensidade da dor	VAS
(PARK, KYOUNG SUN *et al.*, 2023)	2023	Intensidade da dor, incapacidade e qualidade de vida	VAS, NRS, ODI e EQ-5D-5L
(KAŁUŻNA *et al.*, 2023)	2023	Intensidade da dor, incapacidade e qualidade de vida	NRS, ODI e SF-36
(KOLU *et al.*, 2023)	2023	Intensidade da dor	N.A
(BOAS FERNANDES *et al.*, 2023)	2023	Intensidade da dor	NRS
(YANG, HSUEH-HUA *et al.*, 2023)	2023	Intensidade da dor	Inventário Breve de Dor
(O'HAGAN *et al.*, 2023)	2023	Intervenção de reforço	Escala de benefício percebido
(HERNANDEZ-LUCAS *et al.*, 2023)	2023	Intensidade da dor, incapacidade, qualidade de vida e cinesiofobia	VAS, RMDQ, SF-36 e TSK
(ASADA *et al.*, 2023)	2023	Intensidade da dor	NRS
(FORTIN *et al.*, 2023)	2023	Morfologia do músculo multífido	RNM
(SIĞLAN; ÇOLAK, 2023)	2023	Intensidade da dor, mobilidade, ADM e flexibilidade	N.A
(SONG *et al.*, 2023)	2023	Intensidade da dor	N.A
(UZLIFATIN *et al.*, 2023)	2023	Intensidade da dor e Incapacidade	NPRS e RMDQ
(ROSEEN *et al.*, 2023)	2023	Incapacidade e intensidade da dor	RMDQ e Escala de Avaliação da Dor da Defesa e dos Veteranos.
(PARK, CHANHEE; YI; *et al.*, 2023)	2023	Incapacidade	ODI
(IBRAHIM, AMINU A.; AKINDELE; GANIYU, 2023)	2023	Intensidade da dor	NPRS
(RHON *et al.*, 2023)	2023	Incapacidade da dor	RMDQ
(LE MOAL *et al.*, 2023)	2023	Força isocinética, flexibilidade da mobilidade, resistência, intensidade da dor e incapacidade	N.A
(ZADRO, 2023)	2023	Intensidade da dor e Incapacidade	NRS e ODI
(KHUSHBOO KUIYA; SNEHA JOSHI; VIJAY KAUSHIK, 2023)	2023	Intensidade da dor	NPRS
(SADLER *et al.*, 2023)	2023	Intensidade da dor e Incapacidade	N.A
(AMSTEL *et al.*, 2023)	2023	ADM e qualidade de vida	Inclinômetro e EQ-5D-3L

(PARK, CHANHEE; KIM; *et al.*, 2023)	2023	Mobilidade e intensidade da dor	Sensores MyoMotion e VAS
(CUI *et al.*, 2023)	2023	Incapacidade	ODI
(IBRAHIM, ADEL M. *et al.*, 2023)	2023	Reposicionamento lombar, intensidade da dor e a incapacidade	Dinamômetro isocinético, VAS e ODI
(MASROOR *et al.*, 2023)	2023	Intensidade da dor, atividade muscular, incapacidade e qualidade do sono.	VAS, EMG, ODI, Questionário de Qualidade do Sono de Pittsburgh
(OŻÓG *et al.*, 2023)	2023	Estabilidade postural	Posturografia
(ABDULLAH ALI AL-BANAWI *et al.*, 2023)	2023	Intensidade dor, incapacidade e ADM	VAS,ODI e goniômetro
(MARCUZZI *et al.*, 2023)	2023	Questionário de Saúde Musculoesquelética (MSK-HQ)	Aplicativo de auto-manejo baseado em inteligência artificial
(WANG, HONGJIANG *et al.*, 2023)	2023	Intensidade da dor e incapacidade	VAS, ODI e RMDQ
(ALFONSO-MORA *et al.*, 2023)	2023	Intensidade da dor e incapacidade	N.A
(YAMADA, 2023)	2023	Intensidade da dor, sensibilização central, incapacidade, catastrofização, cinesiofobia e ansiedade, depressão e qualidade de vida	Inventário Breve de Dor, Inventário de Sensibilização Central (ISC), RMDQ, Escala de Catastrofização da Dor, TSK, Escala Hospitalar de Ansiedade e Depressão,SF-6D
(AHMAD *et al.*, 2023)	2023	Intensidade da dor	NRS
(AYTAR *et al.*, 2023)	2023	Intensidade da dor, incapacidade e cinesiofobia	VAS, ODI e TSK
(KARARTI *et al.*, 2023)	2023	Qualidade dos movimentos e a capacidade de exercício	N.A
(SCHRÖDER *et al.*, 2023)	2023	Encaminhamento para consulta com especialista	N.A
("The Effect of Core Stability Exercises Combined With Abdominal Hollowing on Postural Balance in Patients With Non-specific Chronic Low Back Pain: A Randomized Controlled Trial", 2023)	2023	Intensidade da dor, incapacidade e propriocepção	VAS, ODI, goniometro
(AMIRI, 2023)	2023	Oscilação postural	N.A
(MOHAMMADI; LETAFATKAR; JAFARNEZHADGERO, 2023)	2023	Estabilidade do core	N.A
(PINHO *et al.*, 2023)	2023	Intensidade da dor e limiar de dor à pressão	N.A
(SUTTON *et al.*, 2023)	2023	Melhora de dor e qualidade de vida em mulheres pós-menopáusicas	Exercício de estabilidade muscular central
(PASSOS *et al.*, 2024)	2024	Incapacidade e Intensidade da dor	RMDQ,EVA
(ELBASTI; AKCAN, 2024)	2024	Intensidade da dor, incapacidade e qualidade do sono	VAS, RMDQ e PSQI
(SIDIQ *et al.*, 2024)	2024	Intensidade da dor e incapacidade	VAS e RMDQ

(DARNALL *et al.*, 2024)	2024	Catastrofização da Dor	Escala de Catastrofização da Dor (PCS)
(IOSUB; TIRLA; LAZAR, 2024)	2024	Intensidade da dor, Parâmetros psicométricos e incapacidade	VAS, Hamilton Depression Rating Scale e RMDQ
(KARADUMAN; ATAŞ BALCI, 2024)	2024	Incapacidade, Intensidade da dor e cinesiofobia	ODI, NRS e TSK
(PECOS-MARTÍN *et al.*, 2024)	2024	Intensidade da dor	VAS
(GERAGHTY *et al.*, 2024)	2024	Incapacidade da dor	RMDQ
(DEMIRCIOĞLU; ÖZDEN; GENÇ, 2024)	2024	Mobilidade, força, Intensidade da dor e inncapacidade	Teste de chober modificado, teste de alcance dos dedos ao chão, Dinamômetro, VAS e ODI
(SOYLU *et al.*, 2024)	2024	Ativação musular	EMG
(FANUSCU *et al.*, 2024)	2024	Incapacidade, qualidade de vida, castatrofização e Intensidade da dor	VAS
(OKUDAN; ÇELIK; KOBAN, 2024)	2024	Intensidade da dor, Incapacidade e crenças sobre dor	NRS, ODI e Questionário de Crenças sobre a Dor (QCD)
(OZ; ULGER, 2024)	2024	Intensidade da dor e incaacidade	VAS e OD
(PEHLEVAN; ŞEVGIN, 2024)	2024	Intensidade da dor, fadiga, qualidade d sono e Incapacidade	Questionário de Dor de McGill (MPQ), VAS, a Escala de Gravidade da Fadiga (FSS), o PSQ) e ODI
(ARMBRUST *et al.*, 2024)	2024	Intensidade da dor, incapacidade e qualidade de vida	N.A.
(ELABD; ELABD, 2024a)	2024	Intensidade da dor	N.A
(ELABD; ELABD, 2024b)	2024	Incapacidade	ODI
(CANKAYA; PALA, 2024)	2024	Intensidade da dor, incapaciadade e ADM	EVA, ODI e Goniometro
(LÓPEZ-MARCOS *et al.*, 2024)	2024	Intensidade da dor	NRS
(NEWTON *et al.*, 2024)	2024	Incapaciadade	RMDQ
(KASIMIS *et al.*, 2024)	2024	Intensidade da dor, o limiar de dor à pressão, incapacidade, cinesiofobia, a catastrofização e a capacidade funcional	NPRS, RMDQ, Algometro, TKS, PCS e Back Performance Scale (BPS)
(MUDDA *et al.*, 2024)	2024	Intensidade da dor	VAS
(PLANDOWSKA *et al.*, 2024)	2024	Intensidade da dor, desconforto lombar e incapacidade	VAS, DL e ODI
(RABIEEZADEH *et al.*, 2024)	2024	Intensidade da dor, incapacidade e qualidade de vida	VAS, ODI e SF-36
(POPOVICH *et al.*, 2024)	2024	Intensidade da dor e a incapacidade	NRS e ODI
(ÇIFTÇI; USGU, 2024)	2024	Intensidade da dor	McGill Short Form, VAS
(ONSY *et al.*, 2024)	2024	Intensidade da dor, incapacidade e ganho de massa e força	NRS, ODI, ultrassonografia e Dinamômetro
(KAROUI *et al.*, 2024)	2024	Força muscular	Dinamômetro isocinético

(INYOUNG, 2024)	2024	Contração diafragmática e a atividade muscular do tronco	Ultrassonografia e eletromiografia
(VERA-SAURA *et al.*, 2024)	2024	Intensidade da dor, incapacidade e cinesiofobiaa	VAS, ODI e TSK
(MA *et al.*, 2024)	2024	Intensidade da dor	VAS
(ORTIZ *et al.*, 2024)	2024	Intensidade da dor, registro de medicamcemtos, atividade física e incapacidade	NRS, Avaliação Global de Mudança (GROC) e ODI
(RAJFUR, JOANNA *et al.*, 2024)	2024	Incapacidade e Intensidade da dor	RMDQ e VAS
(YANG, YANG *et al.*, 2024)	2024	Incapacidade	ODI
(BLANCO-GIMÉNEZ; VICENTE-MAMPEL; GARGALLO; MAROTO-IZQUIERDO; *et al.*, 2024)	2024	Intensidade da dor e atividade elétrica muscula	VAS e EMG
(LIN *et al.*, 2024)	2024	ADM e Limiar de dor	N.A
(YU, JI-EUN, 2024)	2024	Intensidade da dor, ansiedade e depressão e incapacidade	VAS e ODI
(FARLEY *et al.*, 2024)	2024	Incapacidade e intensidade da dor	ODI e VAS
(SON *et al.*, 2024)	2024	Viabilidade, Incapacidade, intensidade da dor e qualidade de vida	Registro de frequência, ODI, VAS SF-36
(TOMÁS-RODRÍGUEZ *et al.*, 2024)	2024	Intensidade da dor, Sensibilização central, Catastrofização da dor, cinesiofobia	NRS, Inventário de Sensibilização Central, PCS e TSK
(FREITAS *et al.*, 2024)	2024	O limiar de dor à pressão	Algômetro digital
(PAGNEZ *et al.*, 2024)	2024	Distância L5-S1 e PPT	US e Algômetro digital
(KHALEDI; GHEITASI, 2024)	2024	Intensidade da dor e incapacidade	VAS e ODI
(LARA-PALOMO *et al.*, 2024)	2024	Incapacidade e intensidade da dor	RMDQ e VAS
(HARRISON *et al.*, 2024)	2024	Crescimento da coluna vertebral e intensidade da dor	Estadiometro
(ZAFAR *et al.*, 2024)	2024	Ativação muscular, intensidade da dor, incapacidade e desempenho funcional	EMG, VAS, RMDQ, Avaliação Progressiva de Levantamento Isoinercial (PILE)
(PARK, CHUL-HYUN *et al.*, 2024)	2024	Intensidade da dor	ENA
(PAPOLI; HOSSEINI; MIRKARIMPOUR, 2024)	2024	Intensidade da dor	EVA
(SAFYELDEEN *et al.*, 2024)	2024	Limiar de dor à pressão e incapacidade	Pressure Pain Threshold (PPT) com algômetro e ODI
(ALIZAD ARABLOUYE BISHE; BABAKHANI; KAZEMI SUFI, 2024)	2024	Intensidade da dor, rigidez muscular e ADM	N.A
(KO, JONG HYEON *et al.*, 2024)	2024	Rigidez muscular, Intensidade da dor e função física	Myoton PRO, VAS e teste de equilíbrio
(POCOVI *et al.*, 2024)	2024	Número de dias até a primeira recorrência de dor	Registro
(DELANO *et al.*, 2024)	2024	Incapacidade	ODI

(NGO; TRINH; TANG, 2024)	2024	Intensidade da dor, necessidade de medicação, incapacidade e efeitos adversos	VAS, ODI e registro
(TANKHA *et al.*, 2024)	2024	Intensidade da dor e Incapacidade	NRS e RMDQ
(YANG, CHAO *et al.*, 2024)	2024	Intensidade do eco, espessura, intensidade da dor e incapacidade	US, RMDQ e VAS
(SHI *et al.*, 2024)	2024	Incapacidade	ODI
(HUANG, WEN-HUNG; TANG; SHIH, 2024)	2024	Inclinação pélvica, diferença de inclinação anterior do ílio, intensidade da dor, funcionalidade e incapacidade.	VAS, escala funcional específica do paciente (PSFS) e ODI
(ASGARI; RAEISI, 2024)	2024	Intensidade da dor e incapacidade e percepção subjetiva (PS) nas direções	McGill, RMDQ e plataforma Zebris
(YILDIRIM *et al.*, 2024)	2024	ADM, Intensidade da dor e incapacidade	N.A
(AKILA.B *et al.*, 2024)	2024	Intensidade da dor, incapacidade e ADM	VAS, RMDQ e goniometria
(KOSIĆ; MALNAR; LEKIĆ, 2024)	2024	Incapacidade	RMDQ
(ALQHTANI *et al.*, 2024)	2024	Intensidade da dor, incapacidade e qualidade de vida	N.A
(DAL; KOÇ; BAYAR, 2024)	2024	Intensidade da dor, mobilidade lombar, função, incapacidade e bem-estar	VAS, Teste de Schober Modificado, Escala Funcional de Dor Lombar, RMDQ e SF-36
(KIM, SEUNGMIN *et al.*, 2024)	2024	Intensidade da dor, ADM e incapacidade	NRS, Teste de Schober Modificado e ODI
(LOVO *et al.*, 2024)	2024	Intensidade da dor, incapacidade, crenças , satisfação, qualidade de vida e custos relacionados ao tratamento.	N.A
(XU *et al.*, 2024)	2024	Intensidade da dor e função muscular	VAS, ODI e EMG
(TEGNER *et al.*, 2024)	2024	Redução do comportamento sedentário	Acelerômetros (AX3, Axivity)
(MADDOX *et al.*, 2024)	2024	Intensidade e interferência da dor	Inventário Breve de Dor (BPI)
(CHROBOK *et al.*, 2024)	2024	Incapacidade	ODI
(ÖZDEN *et al.*, 2024)	2024	Intensidade da dor, Catastrofização, incapacidade	VAS, PCS e ODI
(BLANCO-GIMÉNEZ; VICENTE-MAMPEL; GARGALLO; BARAJA-VEGAS; *et al.*, 2024)	2024	Incapacidade	ODI
(LI, XIN *et al.*, 2024)	2024	Intensidade da dor, incapacidade	NPRS e ODI
(ZHANG *et al.*, 2024)	2024	Intensidade da dor e incapacidade	N.A
(ZHENG, FUMING *et al.*, 2024)	2024	Incapacidade e intensidade da dor	RMDQ e NRS
(FARRAGHER *et al.*, 2024)	2024	Incapacidade	RMDQ
(LUQMAN SULAIMAN *et al.*, 2024)	2024	Resistência muscular central, inclinação pélvica lateral e flexibilidade	N.A
(MERLO *et al.*, 2024)	2024	Intensidade da dor, equilíbrio postural, incapacidade, cinesiofobia, crenças e Catastrofização	NRS, ODI, RMDQ, TSK, FABQ e PCS

(AVILA *et al.*, 2024)	2024	Intensidade da dor e função	NRS e Escala Funcional Específica do Paciente
(CORREIA BULHÕES *et al.*, 2024)	2024	Intensidade da dor	NRS
(ADENIS *et al.*, 2024)	2024	Incapacidade	N.A
(BENEDICT *et al.*, 2024)	2024	Estresse pós-traumático, Incapacidade e intensidade da dor	N.A
(DESAI; SRINIVASAN, 2024b)	2024	Intensidade da dor, incapacidade	NPRS e ODI
(HONG *et al.*, 2024)	2024	Intensidade da dor, função, ansiedade e depressão	N.A
(KANLI, 2024)	2024	Ansiedade e Depressão, intensidade da dor, Incapacidade	BDI, VAS, RMDQ, Perfil de Saúde de Nottingham (NHP)
(KHAN *et al.*, 2024)	2024	Intensidade da dor, incapacidade e resistência muscular	NPRS, RMDQ e Unidade de Biofeedback de Pressão (PBU)
(YALFANI *et al.*, 2024)	2024	Oscilação postural e função física	Teste de levantar da cadeira em 30 segundos (30CST) e o teste de levantar e andar cronometrado (TUG).
(YALFANI; ASGARPOOR, 2024)	2024	Intensidade da dor, cinesiofobia e incapacidade	N.A
(AUGUSTINA *et al.*, 2024)	2024	Intensidade da dor e incapacidade	NRS e ODI
(JURAK *et al.*, 2025)	2025	Incapacidade e Intensidade da dor	VAS e ODI
(MOUDJARI *et al.*, 2025)	2025	Controle motor	Blueback physio
(SOTIROPOULOS; PLAVOUKOU; GEORGOUDIS, 2025)	2025	Intensidade da dor, Incapacidade, cinesiofobia e Propriocepção	Short-Form McGill Pain Questionnaire (SFMPQ); Roland Morris Disability Questionnaire (RMDQ); ampa Scale of Kinesiophobia (TSK); baropodômetro
(ULUSOY; IYIGUN, 2025)	2025	Intensidade da dor e incapacidade	VAS e ODI
(XIAO *et al.*, 2025)	2025	Intensidade da dor e incapacidade	NRS e ODI
(LARA-PALOMO *et al.*, 2025)	2025	Intensidade da dor e incapacidade	NRS e RMDQ
(GUL *et al.*, 2025)	2025	Intensidade da dor e incapacidade	NRS RMDQ
(HUANG, HUANJIE *et al.*, 2025)	2025	Intensidade da dor e incapacidade	VAS e ODI
(MOUHLI *et al.*, 2025)	2025	Intensidade da dor e incapacidade	VAS e ODI
(UCGUN; KAYA; KULLI, 2025)	2025	Intensidade da dor e incapacidade	VAS e RMQD
(VEERAMACHANENI *et al.*, 2025)	2025	Intensidade da dor	NRS
(GEDIN *et al.*, 2025)	2025	Intensidade da dor, incapacidade e qualidade de vida	NPRS, ODI, EQ-5D-5L
(LIU, YIXUAN *et al.*, 2025)	2025	Intensidade da dor e controle motor	VAS e teste de flexão, extensão, ponte e prancha

(ROSENSTEIN *et al.*, 2025)	2025	Volume muscular, força, Intensidade da dor e incapacidade	ressonância magnética, Dinamômetro, NRS e ODI
(ESPIN *et al.*, 2025)	2025	Intensidade da dor, frequência de dor e aspectos psicológicos	NRS, números de dias e escalas de Goldberg
(TOMSCHI; ZSCHUNKE; HILBERG, 2025)	2025	Sensibilidade à dor	Pressure Pain Threshold (PPT)
(CHEN, ZIYAN; WANG, 2025)	2025	Intensidade da dor	VAS
(LIU, MINGSHENG *et al.*, 2025)	2025	Intensidade da dor e incapacidade	VAS e ODI
(LI, YING *et al.*, 2025)	2025	Intensidade da dor e incapacidade	VAS e ODI
(EROL; BURAK; ELBASAN, 2025)	2025	Intensidade da dor e incapacidade	VAS e ODI
(NAGESWARI *et al.*, 2025)	2025	Incapacidade e Intensidade da dor	ODI, NPRS
(SHAHEEN; BELGEN KAYGISIZ, 2025)	2025	Intensidade da dor e incapacidade	NRS e ODI
(AREFI *et al.*, 2025)	2025	Intensidade da dor	VAS
(MUDD *et al.*, 2025)	2025	Incapacidade	RMDQ
(ENGEL *et al.*, 2025)	2025	Intensidade da dor	VAS
(GERAGHTY *et al.*, 2025)	2025	Incapacidade	RMDQ
(NEASON *et al.*, 2025)	2025	Intensidade da dor e incapacidade	VAS e ODI
(BHATTARAI; KHANNA, 2025)	2025	Intensidade da dor e incapacidade	VAS e Questionário de Qualidade de Vida Modificado (MODQ)
(ÇETINYOL; SAKA; ÇETINKAYA, 2025)	2025	Intensidade da dor e flexibilidade	VAS, Teste de Sentar e Alcançar (TSA), o Teste de Elevação da Perna Estendida (TEPE) e o Teste de Schober Modificado.
(DAR; GOLDBERG, 2025)	2025	Intensidade da dor e incapacidade funcional	EVA e ODI

REFERENCES

ABBASI, Soheila; HADIAN RASANANI, Mohammad-Reza; GHOTBI, Nastaran; OLYAEI, Gholam Reza; BOZORGMEHR, Ali; *et al.* Short-term effect of kinesiology taping on pain, functional disability and lumbar proprioception in individuals with nonspecific chronic low back pain: a double-blinded, randomized trial. *Chiropractic & Manual Therapies*, v. 28, n. 1, p. 63, 20 dez. 2020.

ABBASI, Soheila; HADIAN RASANANI, Mohammad Reza; GHOTBI, Nastaran; OLYAEI, Gholam Reza; RASOULI, Omid. The Effect of Kinesio Taping on Pain, Functional Disability, and Trunk Range of Motion in People With Nonspecific Chronic Low Back Pain: A Single-group Pretest-Posttest Trial. *Journal of Modern Rehabilitation*, v. 14, n. 1, p. 47–54, 28 maio 2020.

ABDEL-AZIEM, Amr A. *et al.* The Effects of Stabilization Exercises Combined With Pelvic Floor Exercise in Women With Nonspecific Low Back Pain: A Randomized Clinical Study. *Journal of Chiropractic Medicine*, v. 20, n. 4, p. 229–238, dez. 2021.

ABDELBASSET, Walid Kamal *et al.* A Prospective Comparative Study of Pulsed High-Intensity Laser Therapy and Pulsed Electromagnetic Field on Chronic Nonspecific Low Back Pain. *Photobiomodulation, Photomedicine, and Laser Surgery*, v. 39, n. 5, p. 362–368, 1 maio 2021.

ABDELBASSET, Walid Kamal *et al.* A Randomized Comparative Study between High-Intensity and Low-Level Laser Therapy in the Treatment of Chronic Nonspecific Low Back Pain. *Evidence-Based Complementary and Alternative Medicine*, v. 2020, n. 1, 29 jan. 2020.

ABDULLAH ALI AL-BANAWI, Lina *et al.* Effects of the Addition of Hands-on Procedures to McKenzie Exercises on Pain, Functional Disability and Back Mobility in Patients with Low Back Pain: A Randomised Clinical Trial. *Malaysian Journal of Medical Sciences*, v. 30, n. 3, p. 122–134, 27 jun. 2023.

ADENIS, Nicolas *et al.* Pain neuroscience education is not superior to spinal physiology and ergonomics education within a short multidisciplinary rehabilitation program: A randomized controlled trial. *Musculoskeletal Science and Practice*, v. 74, p. 103176, nov. 2024.

AFZAL, Muhammad Waqar *et al.* Effects of virtual reality exercises and routine physical therapy on pain intensity and functional disability in patients with chronic low back pain. *Journal of the Pakistan Medical Association*, v. 72, n. 3, 3 fev. 2022.

AGRE, Sharayu; AGRAWAL, Ronika. “At-Job” Exercise Program versus Ergonomic Brochure on Pain and Disability in Preprimary School Teachers with Low Back Pain. *Indian journal of physical therapy and research*, v. 5, n. 2, p. 176–181, jul. 2023.

AHMAD, Sahar Nazary Soltan *et al.* Comparison of cognitive functional therapy and movement system impairment treatment in chronic low back pain patients: a randomized controlled trial. *BMC Musculoskeletal Disorders*, v. 24, n. 1, p. 684, 29 ago. 2023.

AHMADI, Hanieh *et al.* Comparison of the effects of the Feldenkrais method versus core stability exercise in the management of chronic low back pain: a randomised control trial. *Clinical Rehabilitation*, v. 34, n. 12, p. 1449–1457, 29 dez. 2020.

AHMADIZADEH, Zahra *et al.* The Effect of Stabilization Exercises Along With Self-care Training on Transverse Abdominal Activity, Pain, and Disability in Mothers With Low Back Pain Having Children With Cerebral Palsy. *American Journal of Physical Medicine & Rehabilitation*, v. 99, n. 2, p. 156–160, fev. 2020.

AHMED, Usman Abba; MAHARAJ, Sonill Sooknunan; VAN OOSTERWIJCK, Jessica. Effects of dynamic stabilization exercises and muscle energy technique on selected biopsychosocial outcomes for patients with chronic non-specific low back pain: a double-blind randomized controlled trial. *Scandinavian Journal of Pain*, v. 21, n. 3, p. 495–511, 27 jul. 2021.

AISWARYA K RAVINDRAN *et al.* Effectiveness of Aerobic Exercise Versus Pilates in Postmenopausal Women with Non-Specific Chronic Low Back Pain. *Indian Journal of Physiotherapy & Occupational Therapy - An International Journal*, v. 16, n. 2, p. 1–8, 13 abr. 2022.

AKHTAR, M. Shahid *et al.* Comparison between Kinesio Taping & Traditional Physical Therapy Rehabilitation for treatment of Non-Specific Back Pain. *Pakistan Journal of Medical and Health Sciences*, v. 16, n. 12, p. 81–83, 31 dez. 2022.

AKHTAR, Muhammad Waseem; KARIMI, Hossein; GILLANI, Syed Amir. Effectiveness of core stabilization exercises and routine exercise therapy in management of pain in chronic nonspecific low back pain: A randomized controlled clinical trial. *Pakistan Journal of Medical Sciences*, v. 33, n. 4, 23 ago. 2017.

AKILA.B *et al.* Effectiveness of Lumbar Stabilization Exercise in Improving Lumbar Spine Endurance by Using Sorenson's Test and EMG Analysis for People with Chronic Low Back Ache. *Indian Journal of Physiotherapy & Occupational Therapy - An International Journal*, v. 18, p. 1–6, 21 jan. 2024.

AKSOY, C.; KARAN, A.; DIRAÇOĞLU, D. Low back pain: Results of an open clinical trial comparing the standard treatment alone to the combination of standard treatment and thiocolchicoside. *Journal of Orthopaedics and Traumatology*, v. 3, n. 2, p. 103–108, out. 2002. Disponível em: <https://link.springer.com/article/10.1007/s101950200036>. Acesso em: 8 jun. 2026.

ALARAB, Azzam; SHAMEH, Ratib Abu; AHMAD, Muntaser S. Muscle contraction exercise for low back pain. *Hong Kong Physiotherapy Journal*, v. 43, n. 01, p. 53–60, 31 jun. 2023.

ALFONSO-MORA, Margareth Lorena *et al.* Mézières Method as a practice of embodiment in patients with low back pain: a mixed study. *Annals of Medicine*, v. 55, n. 2, 12 dez. 2023.

ALI, Md. Nasir; SETHI, Kritika; NOOHU, Majumi M. Comparison of two mobilization techniques in management of chronic non-specific low back pain. *Journal of Bodywork and Movement Therapies*, v. 23, n. 4, p. 918–923, out. 2019.

ALIKHAJEH, Yaser; BARABADI, Elyas; MOHAMMAD RAHIMI, Gholam Rasul. A Comparison of 6 Weeks of Aquatic Exercise and Kinesio Taping in Patients With Chronic Nonspecific Low Back Pain. *Journal of Sport Rehabilitation*, v. 30, n. 1, p. 37–42, 1 jan. 2021.

ALIZAD ARABLOUYE BISHE, Atieh; BABAKHANI, Farideh; KAZEMI SUFI, Siamak. A Comparison of Combined Pilates-Kinesio Tape and Mulligan-Kinesio Tape Exercises on Pain, Lumbar Range of Motion, Hamstring Strength, and Pelvic Rotation in Women with Non-specific Chronic Low Back Pain. *Journal of Clinical Research in Paramedical Sciences*, v. 13, n. 1, 17 ago. 2024.

ALKHATHAMI, Khalid *et al.* Effectiveness of Spinal Stabilization Exercises on Movement Performance in Adults with Chronic Low Back Pain. *International Journal of Sports Physical Therapy*, v. 18, n. 1, 1 fev. 2023.

ALMEIDA, Nicole *et al.* Immediate Effects of the Combination of Interferential Therapy Parameters on Chronic Low Back Pain: A Randomized Controlled Trial. *Pain Practice*, v. 20, n. 6, p. 615–625, 2 jul. 2020.

ALQHTANI, Raee Saeed *et al.* Efficacy of Core-Strengthening and Intensive Dynamic Back Exercises on Pain, Core Muscle Endurance, and Functional Disability in Patients with Chronic Non-Specific Low Back Pain: A Randomized Comparative Study. *Journal of Clinical Medicine*, v. 13, n. 2, p. 475, 15 jan. 2024.

ALRWAILY, Muhammad *et al.* Stabilization exercises combined with neuromuscular electrical stimulation for patients with chronic low back pain: a randomized controlled trial. *Brazilian Journal of Physical Therapy*, v. 23, n. 6, p. 506–515, nov. 2019.

AL-SHAREEF, Amal T.; OMAR, Mohammed T.A.; IBRAHIM, Amal H.M. Effect of Kinesio Taping on Pain and Functional Disability in Chronic Nonspecific Low Back Pain. *Spine*, v. 41, n. 14, p. E821–E828, 15 jul. 2016.

ALTINBILEK, Turgay. A comparison of application frequency of physical therapy modalities in patients with chronic mechanical low back pain. *Turkish Journal of Physical Medicine and Rehabilitation*, v. 66, n. 2, p. 201–209, 8 jun. 2020.

ALTMAIER, Elizabeth M. *et al.* The effectiveness of psychological interventions for the rehabilitation of low back pain: a randomized controlled trial evaluation. *Pain*, v. 49, n. 3, p. 329–335, 1992. Disponível em: <https://journals.lww.com/pain/fulltext/1992/06000/the_effectiveness_of_psychological_interventions.8.aspx>. Acesso em: 8 jun. 2026.

ALVANI, Ehsan; SHIRVANI, Hossein; SHAMSODDINI, Alireza. Neuromuscular exercises on pain intensity, functional disability, proprioception, and balance of military personnel with chronic low back pain. *The Journal of the Canadian Chiropractic Association*, v. 65, n. 2, p. 193–206, ago. 2021.

ALZAHRANI, Hosam *et al.* Wearables-based walking program in addition to usual physiotherapy care for the management of patients with low back pain at medium or high risk of chronicity: A pilot randomized controlled trial. *PLOS ONE*, v. 16, n. 8, p. e0256459, 26 ago. 2021.

AMARAL, S.; PÁSSARO, A.C.; CASAROTTO, R.A. Effect of the association of continuous shortwave diathermy and Pilates-based exercises on pain, depression, and anxiety in chronic non-specific low back pain: a randomized clinical trial. *Brazilian Journal of Medical and Biological Research*, v. 56, 2023.

AMIRI, Fatemeh. Comparing the effectiveness of flexi-bar and stability exercises on postural control in chronic nonspecific low back pain: A randomized controlled study. *Turkish Journal of Physical Medicine and Rehabilitation*, v. 69, n. 4, p. 510–519, 1 out. 2023.

AMPOMAH, KWASI *et al.* Blood Flow–restricted Exercise Does Not Induce a Cross-Transfer of Effect: A Randomized Controlled Trial. *Medicine & Science in Sports & Exercise*, v. 51, n. 9, p. 1817–1827, set. 2019.

AMSTEL, Robbert Van *et al.* Fascia Tissue Manipulations in Chronic Low Back Pain: A Pragmatic Comparative Randomized Clinical Trial of the 4xT Method® and Exercise Therapy. *Life*, v. 14, n. 1, p. 7, 20 dez. 2023.

ANAN, Tomomi *et al.* Effects of an Artificial Intelligence–Assisted Health Program on Workers With Neck/Shoulder Pain/Stiffness and Low Back Pain: Randomized Controlled Trial. *JMIR mHealth and uHealth*, v. 9, n. 9, p. e27535, 24 set. 2021.

ANDERSEN, Tonny Elmose *et al.* A randomized controlled trial of brief Somatic Experiencing for chronic low back pain and comorbid post-traumatic stress disorder symptoms. *European Journal of Psychotraumatology*, v. 8, n. 1, 1 jan. 2017.

ANNASWAMY, Thiru M. *et al.* Lumbar Bracing for Chronic Low Back Pain. *American Journal of Physical Medicine & Rehabilitation*, v. 100, n. 8, p. 742–749, ago. 2021.

ANSARI, S.; ELMIEH, A.; ALIPOUR, A. The effect of aquatic exercise on functional disability, flexibility and function of trunk muscles in postmenopausal women with chronic non-specific low back pain: Randomized controlled trial. *Science & Sports*, v. 36, n. 3, p. e103–e110, jun. 2021.

ARAMPATZIS, Adamantios *et al.* A random-perturbation therapy in chronic non-specific low-back pain patients: a randomised controlled trial. *European Journal of Applied Physiology*, v. 117, n. 12, p. 2547–2560, 19 dez. 2017.

AREEUDOMWONG, Pattanasin *et al.* A randomized controlled trial on the long-term effects of proprioceptive neuromuscular facilitation training, on pain-related outcomes and back muscle activity, in patients with chronic low back pain. *Musculoskeletal Care*, v. 15, n. 3, p. 218–229, 28 set. 2017.

AREFI, Sara *et al.* MLS Laser Reduce Pain in Patients with Chronic Low Back Pain. *Anesthesiology and Pain Medicine*, v. 15, n. 1, 25 fev. 2025.

ARGUISUELAS, María D. *et al.* Effects of Myofascial Release in Nonspecific Chronic Low Back Pain. *Spine*, v. 42, n. 9, p. 627–634, 1 maio 2017.

ARMBRUST, Danilo *et al.* Effects of osteopathic manipulative treatment associated with transcranial direct current stimulation in individuals with chronic low back pain: A double-blind, randomised placebo-controlled trial. *Clinical Rehabilitation*, v. 38, n. 12, p. 1609–1621, 3 dez. 2024.

ASADA, Fuminari *et al.* Effect of quick simple exercise on non-specific low back pain in Japanese workers: a randomized controlled trial. *Environmental Health and Preventive Medicine*, v. 28, n. 0, p. 22–00203, 2023.

ASGARI, Mahsa; RAEISI, Zahra. Effects of Comprehensive Physical Exercises Combined With Cognitive Functional Therapy on Women With Low Back Pain. *Iranian Rehabilitation Journal*, v. 22, n. 3, p. 395–404, 1 set. 2024.

AUGER, Kyle *et al.* Effects of osteopathic manipulative treatment and bio-electromagnetic energy regulation therapy on lower back pain. *Journal of Osteopathic Medicine*, v. 121, n. 6, p. 561–569, 25 maio 2021.

AUGUSTINA, Jenifer S. *et al.* Comparing the effectiveness of laser therapy with pelvic floor strengthening exercises and interferential therapy in reducing postpartum low back pain. *Fizjoterapia Polska*, v. 24, n. 5, p. 432–435, 31 dez. 2024.

AURE, Olav Frode; HOEL NILSEN, Jens; VASSELJEN, Ottar. Manual therapy and exercise therapy in patients with chronic low back pain: a randomized, controlled trial with 1-year follow-up. *Spine*, v. 28, n. 6, p. 525–531, mar. 2003. Disponível em: <https://pubmed.ncbi.nlm.nih.gov/12642755/>. Acesso em: 8 jun. 2026.

AVILA, Leonardo *et al.* Effectiveness of Cognitive Functional Therapy Versus Core Exercises and Manual Therapy in Patients With Chronic Low Back Pain After Spinal Surgery: Randomized Controlled Trial. *Physical Therapy*, v. 104, n. 1, 1 jan. 2024.

AYTAR, Ayça *et al.* EFFECTIVENESS OF DIFFERENT PHYSICAL THERAPY EXERCISE TECHNIQUES IN CHRONIC LOW BACK PAIN: A RANDOMIZED CONTROLLED STUDY. *Türk Fizyoterapi ve Rehabilitasyon Dergisi*, v. 34, n. 1, p. 141–147, 20 abr. 2023.

AZAB, Alshimaa R. *et al.* Therapeutic value of kinesio taping in reducing lower back pain and improving back muscle endurance in adolescents with hemophilia. *Journal of Musculoskeletal Neuronal Interactions*, v. 20, p. 256–264, jun. 2020.

AZADINIA, Fatemeh *et al.* Effects of lumbosacral orthosis on dynamical structure of center of pressure fluctuations in patients with non-specific chronic low back pain: A randomized controlled trial. *Journal of Bodywork and Movement Therapies*, v. 23, n. 4, p. 930–936, out. 2019.

BACKHAUSEN, Mette G. *et al.* The effects of an unsupervised water exercise program on low back pain and sick leave among healthy pregnant women – A randomised controlled trial. *PLOS ONE*, v. 12, n. 9, p. e0182114, 6 set. 2017.

BAE, Cho-Rong *et al.* Effects of assisted sit-up exercise compared to core stabilization exercise on patients with non-specific low back pain: A randomized controlled trial. *Journal of Back and Musculoskeletal Rehabilitation*, v. 31, n. 5, p. 871–880, 25 out. 2018.

BAGHANI, Parvin. Supervised Group Exercise Therapy Versus Home-based Exercise Therapy: The Effect of McGill Exercises on Pain, Disability and Trunk Stability in Middle-aged Women With Non-specific Chronic Low Back Pain. *Physical Treatments - Specific Physical Therapy Journal*, v. 13, n. 2, p. 127–134, 1 abr. 2023.

BARONE GIBBS, Bethany *et al.* Reducing sedentary behaviour to decrease chronic low back pain: the stand back randomised trial. *Occupational and Environmental Medicine*, v. 75, n. 5, p. 321–327, maio 2018.

BASLER, Heinz Dieter; JÄKLE, Christian; KRÖNER-HERWIG, Birgit. Incorporation of cognitive-behavioral treatment into the medical care of chronic low back patients: a controlled randomized study in German pain treatment centers. *Patient Education and Counseling*, v. 31, n. 2, p. 113–124, 1 jun. 1997. Disponível em: <https://www.sciencedirect.com/science/article/abs/pii/S0738399197009968>. Acesso em: 8 jun. 2026.

BATES, Nathaniel A. *et al.* Physical clinical care and artificial-intelligence-guided core resistance training improve endurance and patient-reported outcomes in subjects with lower back pain. *Clinical Biomechanics*, v. 103, p. 105902, mar. 2023.

BATISTELLA, Carla Elis *et al.* Effects of the Russian current in the treatment of low back pain in women: A randomized clinical trial. *Journal of Bodywork and Movement Therapies*, v. 24, n. 2, p. 118–122, abr. 2020.

BEKKERING, Geertruida E. *et al.* Implementation of Clinical Guidelines on Physical Therapy for Patients With Low Back Pain: Randomized Trial Comparing Patient Outcomes After a Standard and Active Implementation Strategy. *Physical Therapy*, v. 85, n. 6, p. 544–555, 1 jun. 2005. Disponível em: <https://dx.doi.org/10.1093/ptj/85.6.544>. Acesso em: 8 jun. 2026.

BENDIX, A. F. *et al.* Active treatment programs for patients with chronic low back pain: a prospective, randomized, observer-blinded study. *European Spine Journal*, v. 4, n. 3, p. 148–152, jun. 1995. Disponível em: <https://link.springer.com/article/10.1007/BF00298239>. Acesso em: 8 jun. 2026.

BENDIX, Ane F. *et al.* Functional restoration for chronic low back pain. Two-year follow-up of two randomized clinical trials. *Spine*, v. 23, n. 6, p. 717–725, 15 mar. 1998. Disponível em: <https://pubmed.ncbi.nlm.nih.gov/9549794/>. Acesso em: 8 jun. 2026.

BENDIX, Tom *et al.* Functional restoration versus outpatient physical training in chronic low back pain: a randomized comparative study. *Spine*, v. 25, n. 19, p. 2494–2500, 1 out. 2000. Disponível em: <https://pubmed.ncbi.nlm.nih.gov/11013502/>. Acesso em: 8 jun. 2026.

BENEDICT, Timothy M. *et al.* Pain neuroscience education improves post-traumatic stress disorder, disability, and pain self-efficacy in veterans and service members with chronic low back pain: Preliminary results from a randomized controlled trial with 12-month follow-up. *Military Psychology*, v. 36, n. 4, p. 376–392, 3 jul. 2024.

BERBEROĞLU, Utku; ÜLGER, Özlem. Multimedia Instructions for Motor Control Exercises in Patients With Chronic Nonspecific Low Back Pain. *Journal of Sport Rehabilitation*, v. 32, n. 4, p. 424–432, 1 maio 2023.

BHADAURIA, Esha A.; GURUDUT, Peeyoosha. Comparative effectiveness of lumbar stabilization, dynamic strengthening, and Pilates on chronic low back pain: randomized clinical trial. *Journal of Exercise Rehabilitation*, v. 13, n. 4, p. 477–485, 29 ago. 2017.

BHAT P, Vignesh *et al.* Myofascial release versus Mulligan sustained natural apophyseal glides' immediate and short-term effects on pain, function, and mobility in non-specific low back pain. *PeerJ*, v. 9, p. e10706, 15 mar. 2021.

BHATTARAI, Damodar; KHANNA, Archana. Effect of Core Stabilization Exercises in Combination with Conventional Physiotherapy in Chronic Low Back Pain. *Integrative and Complementary Therapies*, v. 31, n. 1, p. 12–16, 1 fev. 2025.

BLANCHARD, Agathe *et al.* Technical Feasibility of Supervision of Stretching Exercises by a Humanoid Robot Coach for Chronic Low Back Pain: The R-COOL Randomized Trial. *BioMed Research International*, v. 2022, n. 1, 9 jan. 2022.

BLANCO-GIMÉNEZ, P.; VICENTE-MAMPEL, J.; GARGALLO, P.; BARAJA-VEGAS, L.; *et al.* Clinical relevance of combined treatment with exercise in patients with chronic low back pain: a randomized controlled trial. *Scientific Reports*, v. 14, n. 1, p. 17042, 24 jul. 2024.

BLANCO-GIMÉNEZ, P; VICENTE-MAMPEL, J.; GARGALLO, P; MAROTO-IZQUIERDO, S; *et al.* Effect of exercise and manual therapy or kinesiotaping on sEMG and pain perception in chronic low back pain: a randomized trial. *BMC Musculoskeletal Disorders*, v. 25, n. 1, p. 583, 25 jul. 2024.

BLÖDT, S. *et al.* Qigong versus exercise therapy for chronic low back pain in adults – A randomized controlled non-inferiority trial. *European Journal of Pain*, v. 19, n. 1, p. 123–131, 5 jan. 2015.

BLOMBERG, S. *et al.* Manual therapy with steroid injections--a new approach to treatment of low back pain. A controlled multicenter trial with an evaluation by orthopedic surgeons. *Spine*, v. 19, n. 5, p. 569–577, 1994. Disponível em: <https://pubmed.ncbi.nlm.nih.gov/8184352/>. Acesso em: 8 jun. 2026.

BOAS FERNANDES, Walkyria Vilas *et al.* Effect of osteopathic visceral manipulation for individuals with functional constipation and chronic nonspecific low back pain: Randomized controlled trial. *Journal of Bodywork and Movement Therapies*, v. 34, p. 96–103, abr. 2023.

BORUJENI, Behnam Gholami; YALFANI, Ali. Effect of respiratory muscle training session on ankle muscle activity in athletes with chronic low back pain performing overhead squats: a randomized controlled trial. *International Journal of Evidence-Based Healthcare*, v. 18, n. 2, p. 256–264, jun. 2020.

BRANCHINI, Mirco *et al.* Fascial Manipulation® for chronic aspecific low back pain: a single blinded randomized controlled trial. *F1000Research*, v. 4, p. 1208, 8 jan. 2016.

BROCKOW, T. *et al.* Analgesic effectiveness of subcutaneous carbon-dioxide insufflations as an adjunct treatment in patients with non-specific neck or low back pain. *Complementary Therapies in Medicine*, v. 9, n. 2, p. 68–76, 2001. Disponível em: <https://pubmed.ncbi.nlm.nih.gov/11444885/>. Acesso em: 8 jun. 2026.

BRUEHL, Stephen *et al.* Anger and pain sensitivity in chronic low back pain patients and pain-free controls: The role of endogenous opioids. *Pain*, v. 99, n. 1–2, p. 223–233, set. 2002. Disponível em: <https://pubmed.ncbi.nlm.nih.gov/12237200/>. Acesso em: 8 jun. 2026.

BRUEHL, Stephen *et al.* Does aerobic exercise training alter responses to opioid analgesics in individuals with chronic low back pain? A randomized controlled trial. *Pain*, v. 162, n. 8, p. 2204–2213, ago. 2021.

BURNS, Scott A. *et al.* When Treating Coexisting Low Back Pain and Hip Impairments, Focus on the Back: Adding Specific Hip Treatment Does Not Yield Additional Benefits—A Randomized Controlled Trial. *Journal of Orthopaedic & Sports Physical Therapy*, v. 51, n. 12, p. 581–601, dez. 2021.

BURTON, A. Kim *et al.* Information and advice to patients with back pain can have a positive effect. A randomized controlled trial of a novel educational booklet in primary care. *Spine*, v. 24, n. 23, p. 2484–2491, 1 dez. 1999. Disponível em: <https://pubmed.ncbi.nlm.nih.gov/10626311/>. Acesso em: 8 jun. 2026.

ÇAKMAK, Özge; ATICI, Emine; GÜLŞEN, Mustafa. THE EFFECTS OF INSTRUMENT-ASSISTED SOFT TISSUE MOBILIZATION AND KINESIO TAPING ON PAIN, FUNCTIONAL DISABILITY AND DEPRESSION IN PATIENTS WITH CHRONIC LOW BACK PAIN: A RANDOMIZED TRIAL. *Türk Fizyoterapi ve Rehabilitasyon Dergisi*, v. 33, n. 3, p. 179–186, 20 dez. 2022.

CAÑA-PINO, A.; APOLO-ARENAS, M.D.; FALLA, D.; *et al.* Supervised exercise with or without laser-guided feedback for people with non-specific chronic low back pain. A randomized controlled clinical trial. *Journal of Electromyography and Kinesiology*, v. 70, p. 102776, jun. 2023.

CAÑA-PINO, Alejandro; APOLO-ARENAS, María D.; CARMONA DEL BARCO, Pablo; *et al.* Supervised exercise therapy versus laser-guided exercise therapy on postural control in subjects with non-specific chronic low back pain: a randomized controlled clinical trial. *European Journal of Physical and Rehabilitation Medicine*, v. 59, n. 2, abr. 2023.

CANKAYA, Muhammed Safa; PALA, Omer Osman. Outcomes of Mulligan Concept Applications in Obese Individuals with Chronic Mechanical Low Back Pain: A Randomized Controlled Trial. *Life*, v. 14, n. 6, p. 754, 13 jun. 2024.

CARVALHO, Rodrigo G.S. *et al.* Effectiveness of additional deep-water running for disability, lumbar pain intensity, and functional capacity in patients with chronic low back pain: A randomised controlled trial with 3-month follow-up. *Musculoskeletal Science and Practice*, v. 49, p. 102195, out. 2020.

CASTRO, Julia *et al.* Cognitive functional therapy compared with core exercise and manual therapy in patients with chronic low back pain: randomised controlled trial. *Pain*, v. 163, n. 12, p. 2430–2437, dez. 2022.

CELENAY, Seyda Toprak; KAYA, Derya Ozer; UCURUM, Sevtap Gunay. Adding connective tissue manipulation to physiotherapy for chronic low back pain improves pain, mobility, and well-being: a randomized controlled trial. *Journal of Exercise Rehabilitation*, v. 15, n. 2, p. 308–315, 26 abr. 2019.

ÇETINYOL, Ozan; SAKA, Seda; ÇETINKAYA, Ayşenur. Acute effects of myofascial release technique on flexibility and pain: Outcome for chronic low back pain. *Journal of Bodywork and Movement Therapies*, v. 41, p. 194–198, mar. 2025.

CHALÃ©AT-VALAYER, Emmanuelle *et al.* Long-term effectiveness of an educational and physical intervention for preventing low-back pain recurrence: a randomized controlled trial. *Scandinavian Journal of Work, Environment & Health*, v. 42, n. 6, p. 510–519, jun. 2016.

CHAN, Alexander Y.P. *et al.* Individualised functional restoration plus guideline-based advice vs advice alone for non-reducible discogenic low back pain: a randomised controlled trial. *Physiotherapy*, v. 103, n. 2, p. 121–130, jun. 2017.

CHEING, Gladys L.Y.; HUI-CHAN, Christina W.Y. Transcutaneous electrical nerve stimulation: Nonparallel antinociceptive effects on chronic clinical pain and acute experimental pain. *Archives of Physical Medicine and Rehabilitation*, v. 80, n. 3, p. 305–312, 1999. Disponível em: <https://pubmed.ncbi.nlm.nih.gov/10084439/>. Acesso em: 8 jun. 2026.

CHEN, Zhen-Hua *et al.* [Warming acupuncture combined with "three steps and seven methods" of tuina for chronic nonspecific low back pain of yang deficiency and cold-dampness blockage: a randomized controlled trial]. *Zhongguo zhen jiu = Chinese acupuncture & moxibustion*, v. 42, p. 505–10, maio 2022.

CHEN, Ziyan; WANG, Xiaodong. Effects of balance and hamstring training on nonspecific low back pain: A randomized controlled trial. *Scientific Reports*, v. 15, n. 1, p. 7116, 28 fev. 2025.

CHERKIN, Daniel C *et al.* A comparison of physical therapy, chiropractic manipulation, and provision of an educational booklet for the treatment of patients with low back pain. *New England Journal of Medicine*, v. 339, n. 15, p. 1021–1029, 1998.

CHERKIN, Daniel C. *et al.* Randomized Trial Comparing Traditional Chinese Medical Acupuncture, Therapeutic Massage, and Self-care Education for Chronic Low Back Pain. *Archives of Internal Medicine*, v. 161, n. 8, p. 1081–1088, 23 abr. 2001. Disponível em: <https://jamanetwork.com/journals/jamainternalmedicine/fullarticle/647957>. Acesso em: 8 jun. 2026.

CHHABRA, H. S.; SHARMA, Sunil; VERMA, Shalini. Smartphone app in self-management of chronic low back pain: a randomized controlled trial. *European Spine Journal*, v. 27, n. 11, p. 2862–2874, 15 nov. 2018.

CHILDS, John D.; FLYNN, Timothy W.; FRITZ, Julie M. A perspective for considering the risks and benefits of spinal manipulation in patients with low back pain. *Manual Therapy*, v. 11, n. 4, p. 316–320, nov. 2006. Disponível em: <https://pubmed.ncbi.nlm.nih.gov/16839800/>. Acesso em: 8 jun. 2026.

CHIRADEJNANT, Adit *et al.* Efficacy of "therapist-selected" versus "randomly selected" mobilisation techniques for the treatment of low back pain: A randomised controlled trial. *Australian Journal of Physiotherapy*, v. 49, n. 4, p. 233–241, 2003. Disponível em: <https://pubmed.ncbi.nlm.nih.gov/14632622/>. Acesso em: 8 jun. 2026.

CHOI, Hea-Kyung *et al.* Effects of active rehabilitation therapy on muscular back strength and subjective pain degree in chronic lower back pain patients. *Journal of Physical Therapy Science*, v. 28, n. 10, p. 2700–2702, 2016.

CHROBOK, Leon *et al.* On-Site Physiotherapy in Emergency Department Patients Presenting with Nonspecific Low Back Pain: A Randomized Controlled Trial. *Journal of Clinical Medicine*, v. 13, n. 11, p. 3149, 27 maio 2024.

ÇIFTÇI, Merve; USGU, Serkan. THE EFFECT OF TRADITIONAL MAT EXERCISES VERSUS REFORMER PILATES AND HAMMOCK YOGA ON PAIN, ENDURANCE, BALANCE, DISABILITY, AND QUALITY OF LIFE IN INDIVIDUALS WHO HAD CHRONIC BACK PAIN. *Karya Journal of Health Science*, v. 5, n. 1, p. 1–7, 30 abr. 2024.

CLOSE, Ciara *et al.* A pilot randomised controlled trial (RCT) investigating the effectiveness of reflexology for managing pregnancy low back and/or pelvic pain. *Complementary Therapies in Clinical Practice*, v. 23, p. 117–124, maio 2016.

COATS, Teresa L. *et al.* Effects of valdecoxib in the treatment of chronic low back pain: Results of a randomized, placebo-controlled trial. *Clinical Therapeutics*, v. 26, n. 8, p. 1249–1260, ago. 2004. Disponível em: <https://pubmed.ncbi.nlm.nih.gov/15476906/>. Acesso em: 8 jun. 2026.

CORREIA BULHÕES, Lidiane Cristina *et al.* Effects of a mat Pilates-based exercise program for low back pain in helicopter pilots of the Brazilian Air Force: Randomized controlled trial. *Journal of Bodywork and Movement Therapies*, v. 39, p. 544–549, jul. 2024.

CORTELL-TORMO, Juan M. *et al.* Effects of functional resistance training on fitness and quality of life in females with chronic nonspecific low-back pain. *Journal of Back and Musculoskeletal Rehabilitation*, v. 31, n. 1, p. 95–105, 6 fev. 2018.

COUDEYRE, E. *et al.* Un simple livret d'information peut contribuer à réduire l'incapacité fonctionnelle de patients lombalgiques subaigus et chroniques. Étude contrôlée randomisée en milieu de rééducation. *Annales de Readaptation et de Medecine Physique*, v. 49, n. 8, p. 600–608, nov. 2006. Disponível em: <https://pubmed.ncbi.nlm.nih.gov/16793163/>. Acesso em: 8 jun. 2026.

CRUZ-DÍAZ, David *et al.* Short- and long-term effects of a six-week clinical Pilates program in addition to physical therapy on postmenopausal women with chronic low back pain: a randomized controlled trial. *Disability and Rehabilitation*, v. 38, n. 13, p. 1300–1308, 18 jun. 2016.

CUI, Di *et al.* Randomized-controlled trial assessing a digital care program versus conventional physiotherapy for chronic low back pain. *npj Digital Medicine*, v. 6, n. 1, p. 121, 7 jul. 2023.

DADARKHAH, Afsaneh *et al.* Remote Versus in-Person Exercise Instruction for Chronic Nonspecific Low Back Pain Lasting 12 Weeks or Longer: A Randomized Clinical Trial. *Journal of the National Medical Association*, v. 113, n. 3, p. 278–284, jun. 2021.

DAGENAIS, Simon; HAYFLINGER, D. Cortney; MAYER, John M. Economic Evaluation of an Extended Telehealth Worksite Exercise Intervention to Reduce Lost Work Time from Low Back Pain in Career Firefighters. *Journal of Occupational Rehabilitation*, v. 31, n. 2, p. 431–443, 4 jun. 2021.

DAL, Cansu; KOÇ, Meltem; BAYAR, Banu. The effects of connective tissue massage and classical massage on pain, lumbar mobility, function, disability, and well-being in chronic low back pain: A three-arm randomized controlled trial. *EXPLORE*, v. 20, n. 6, p. 103029, nov. 2024.

DALICHAU, S.; SCHEELE, K. [Effects of elastic lumbar belts on the effect of a muscle training program for patients with chronic back pain]. *Zeitschrift fur Orthopadie und ihre Grenzgebiete*, v. 138, n. 1, p. 8–16, 2000. Disponível em: <https://pubmed.ncbi.nlm.nih.gov/10730357/>. Acesso em: 8 jun. 2026.

DAR, Gali; GOLDBERG, Alon. Dry needling of the gluteus-medius muscle, combined with standard care, for chronic low back pain – a pilot randomized sham-controlled trial. *Journal of Manual & Manipulative Therapy*, v. 33, n. 5, p. 392–400, 3 set. 2025.

DARLOW, Ben *et al.* The Fear Reduction Exercised Early (FREE) approach to management of low back pain in general practice: A pragmatic cluster-randomised controlled trial. *PLOS Medicine*, v. 16, n. 9, p. e1002897, 9 set. 2019.

DARNALL, Beth D. *et al.* Comparison of a Single-Session Pain Management Skills Intervention With a Single-Session Health Education Intervention and 8 Sessions of Cognitive Behavioral Therapy in Adults With Chronic Low Back Pain. *JAMA Network Open*, v. 4, n. 8, p. e2113401, 16 ago. 2021.

DARNALL, Beth D. *et al.* Empowered Relief, cognitive behavioral therapy, and health education for people with chronic pain: a comparison of outcomes at 6-month Follow-up for a randomized controlled trial. *PAIN Reports*, v. 9, n. 1, p. e1116, fev. 2024.

DAYANIR, Ismail Oguz *et al.* Comparison of Three Manual Therapy Techniques as Trigger Point Therapy for Chronic Nonspecific Low Back Pain: A Randomized Controlled Pilot Trial. *The Journal of Alternative and Complementary Medicine*, v. 26, n. 4, p. 291–299, 1 abr. 2020.

DE OLIVEIRA, Ronaldo Fernando *et al.* Directed vertebral manipulation is not better than generic vertebral manipulation in patients with chronic low back pain: a randomised trial. *Journal of Physiotherapy*, v. 66, n. 3, p. 174–179, jul. 2020.

DEFRIN, Ruth *et al.* Conservative correction of leg-length discrepancies of 10mm or less for the relief of chronic low back pain. *Archives of Physical Medicine and Rehabilitation*, v. 86, n. 11, p. 2075–2080, nov. 2005. Disponível em: <https://pubmed.ncbi.nlm.nih.gov/16271551/>. Acesso em: 8 jun. 2026.

DELANO, Enggista Hendriko *et al.* A Comparison Between the Effectiveness of Tepurak Therapy Versus Deep Tissue Massage Stretching on Low Back Function in Nonspecific Low Back Pain. *International Journal of Therapeutic Massage & Bodywork Research Education & Practice*, v. 17, n. 2, p. 12–19, 13 jun. 2024.

DEMIRCIOĞLU, Gamze; ÖZDEN, Ali Veysel; GENÇ, Hazal. Comparison of the efficacy of auricular vagus nerve stimulation and conventional low back rehabilitation in patients with chronic low back pain. *Complementary Therapies in Clinical Practice*, v. 56, p. 101862, ago. 2024.

DENNERLEIN, Jack T. *et al.* The effects of a new seat suspension system on whole body vibration exposure and driver low back pain and disability: Results from a randomized controlled trial in truck drivers. *Applied Ergonomics*, v. 98, p. 103588, jan. 2022.

DEPAOLI LEMOS, V. J. *et al.* Electroacupuncture and Transcutaneous Electrical Nerve Stimulation in Chronic Nonspecific Low Back Pain: a Blind Randomized Clinical Trial. *Muscles, Ligaments and Tendons Journal*, v. 11, n. 4, 31 dez. 2021.

DESAI, Anushka; SRINIVASAN, Anandh. Effect of core strengthening and dynamic hip mobility exercises on non-specific low back pain in female police officers. *Indian Journal of Community Health*, v. 36, n. 4, p. 573–577, 31 ago. 2024a.

DESAI, Anushka; SRINIVASAN, Anandh. Effect of core strengthening and dynamic hip mobility exercises on non-specific low back pain in female police officers. *Indian Journal of Community Health*, v. 36, n. 4, p. 573–577, 31 ago. 2024b.

DESCARREAUX, Martin *et al.* Evaluation of a specific home exercise program for low back pain. *Journal of Manipulative and Physiological Therapeutics*, v. 25, n. 8, p. 497–503, out. 2002.

DESCARREAUX, Martin; BLOUIN, Jean Sébastien; TEASDALE, Normand. Repositioning accuracy and movement parameters in low back pain subjects and healthy control subjects. *European spine journal : official publication of the European Spine Society, the European Spinal Deformity Society, and the European Section of the Cervical Spine Research Society*, v. 14, n. 2, p. 185–191, mar. 2005. Disponível em: <https://pubmed.ncbi.nlm.nih.gov/15759173/>. Acesso em: 8 jun. 2026.

DILEKÇI, Erdal; ÖZKUK, Kağan; KAKI, Barış. The short-term effects of balneotherapy on pain, disability and fatigue in patients with chronic low back pain treated with physical therapy: A randomized controlled trial. *Complementary Therapies in Medicine*, v. 54, p. 102550, nov. 2020.

DIMER DA LUZ, R. *et al.* Neuromuscular electrical stimulation associated with core stability exercises in nonspecific postural low back pain: a randomized clinical trial. *Muscle Ligaments and Tendons Journal*, v. 09, n. 03, p. 446, set. 2019.

DINIZ, Leandro M. *et al.* Effectiveness of brief patient information materials for promoting correct beliefs about imaging and inevitable consequences of low back pain: A randomised controlled trial. *Clinical Rehabilitation*, v. 36, n. 4, p. 527–537, 21 abr. 2022.

DONALDSON, Stuart *et al.* Randomized study of the application of single motor unit biofeedback training to chronic low back pain. *Journal of occupational rehabilitation*, v. 4, n. 1, p. 23–37, mar. 1994. Disponível em: <https://pubmed.ncbi.nlm.nih.gov/24234261/>. Acesso em: 8 jun. 2026.

EBADI, Safoora *et al.* No immediate analgesic effect of diadynamic current in patients with nonspecific low back pain in comparison to TENS. *Journal of Bodywork and Movement Therapies*, v. 22, n. 3, p. 693–699, jul. 2018.

EBADI, Safoora *et al.* The effect of continuous ultrasound on chronic non-specific low back pain: a single blind placebo-controlled randomized trial. *BMC Musculoskeletal Disorders*, v. 13, n. 1, p. 192, 2 dez. 2012.

EKLUND, Andreas *et al.* The Nordic Maintenance Care program: Effectiveness of chiropractic maintenance care versus symptom-guided treatment for recurrent and persistent low back pain—A pragmatic randomized controlled trial. *PLOS ONE*, v. 13, n. 9, p. e0203029, 12 set. 2018.

ELABD, Aliaa M. *et al.* Efficacy of Integrating Cervical Posture Correction With Lumbar Stabilization Exercises for Mechanical Low Back Pain: A Randomized Blinded Clinical Trial. *Journal of Applied Biomechanics*, v. 37, n. 1, p. 43–51, 1 fev. 2021.

ELABD, Aliaa M.; ELABD, Omar M. Effect of aerobic exercises on patients with chronic mechanical low back pain: A randomized controlled clinical trial. *Journal of Bodywork and Movement Therapies*, v. 37, p. 379–385, jan. 2024a.

ELABD, Aliaa M.; ELABD, Omar M. Efficacy of kinesio tape added to lumbar stabilization exercises on adult patients with mechanical low back pain: A randomized, single-blind clinical trial. *Journal of Bodywork and Movement Therapies*, v. 39, p. 218–224, jul. 2024b.

ELBASTI, Muhammet Sahin; AKCAN, Kübra. The Effect of Kinesio Tape Application on Clinical Parameters in Postmenopausal Women With Chronic Low Back Pain: A Randomized Controlled Study. *Cureus*, 20 nov. 2024.

ELKASSABANY, Nabil M. Response to the Kendall Letter. *Pain Medicine*, v. 20, n. 9, p. 1844–1845, 1 set. 2019.

ELLIOTT, Tessa Lillis Poirier *et al.* The Effect of Sitting on Stability Balls on Nonspecific Lower Back Pain, Disability, and Core Endurance. *Spine*, v. 41, n. 18, p. E1074–E1080, 15 set. 2016.

ELNAGGAR, Ibrahim Magdy *et al.* Effects of spinal flexion and extension exercises on low-back pain and spinal mobility in chronic mechanical low-back pain patients. *Spine*, v. 16, n. 8, p. 967–972, 1991. Disponível em: <https://pubmed.ncbi.nlm.nih.gov/1835157/>. Acesso em: 8 jun. 2026.

ENDAMLI, Doğan Burak; BAYRAMLAR, Kezban; TURHAN, Begümhan. Investigation of fascial treatment effectiveness on pain, flexibility, functional level, and kinesiophobia in patients with chronic low back pain. *Physiotherapy Quarterly*, v. 27, n. 3, p. 1–5, 10 set. 2019.

ENGEL, Tilman *et al.* Sensorimotor Stabilization Exercises With and Without Behavioral Treatment in Low Back Pain: Feasibility and Effects of a Multicenter Randomized Controlled Trial. *Archives of Rehabilitation Research and Clinical Translation*, v. 7, n. 1, p. 100430, mar. 2025.

ER, Göktuğ; YÜKSEL, İnci. A comparison of the effects of connective tissue massage and classical massage on chronic mechanical low back pain. *Medicine*, v. 102, n. 15, p. e33516, 14 abr. 2023.

EROL, Erkan; BURAK, Mustafa; ELBASAN, Bülent. Effects of instrument-assisted manipulation and mobilization with movement in chronic non-specific low back pain: A randomized controlled trial. *Journal of Back and Musculoskeletal Rehabilitation*, v. 38, n. 3, p. 640–650, 11 maio 2025.

ESPEJO-ANTÚNEZ, Luis *et al.* Detection of Changes on Parameters Related to Heart Rate Variability after Applying Current Interferential Therapy in Subjects with Non-Specific Low Back Pain. *Diagnostics*, v. 11, n. 12, p. 2175, 23 nov. 2021.

ESPIN, Ander *et al.* Videoconference-Supervised Group Exercise Reduces Low Back Pain in Eldercare Workers: Results from the ReViEEW Randomised Controlled Trial. *Journal of Occupational Rehabilitation*, v. 35, n. 1, p. 66–77, 17 mar. 2025.

EZEMA, Charles Ikechukwu *et al.* Transcutaneous electrical nerve stimulation effects on pain-intensity and endogenous opioids levels among chronic low-back pain patients: A randomised controlled trial. *Journal of Back and Musculoskeletal Rehabilitation*, v. 35, n. 5, p. 1053–1064, 2 set. 2022.

FAGUNDES LOSS, Jefferson *et al.* Immediate effects of a lumbar spine manipulation on pain sensitivity and postural control in individuals with nonspecific low back pain: a randomized controlled trial. *Chiropractic & Manual Therapies*, v. 28, n. 1, p. 25, 3 dez. 2020.

FANUSCU, Aybüke *et al.* Effects of Clinic-based and Telerehabilitation-based Motor Control Exercises in Individuals with Chronic Low-back Pain. *The Clinical Journal of Pain*, v. 40, n. 12, p. 700–708, dez. 2024.

FARLEY, Jedidiah *et al.* The Effect of Combining Spinal Manipulation and Dry Needling in Individuals With Nonspecific Low Back Pain. *The Journal of Pain*, v. 25, n. 8, p. 104506, ago. 2024.

FARRAGHER, Joshua B. *et al.* Neuromuscular Control and Resistance Training for People With Chronic Low Back Pain: A Randomized Controlled Trial. *Journal of Orthopaedic & Sports Physical Therapy*, v. 54, n. 5, p. 350–359, maio 2024.

FAZEL, Fatemeh; ZOLAKTAF, Vahid; NEZHADIAN, Shahram Lenjan. The Effect of Exercise Programs on Pain Management and Motor Control in Patients with Nonspecific Chronic Low Back Pain: A Randomized Matched Subjects Trial. *International Journal of Preventive Medicine*, v. 12, n. 1, jan. 2021.

FELDWIESER, Florian *et al.* Evaluation of biofeedback based bridging exercises on older adults with low back pain: A randomized controlled trial. *Physiotherapy Practice and Research: The Official Journal of The Irish Society of Chartered Physiotherapists*, v. 39, n. 1, p. 15–25, 25 jan. 2018.

FERREIRA, Marcela da Gama e Silva *et al.* Maitland in chronic lumbar pain of young adults improves pain and functionality. *Manual Therapy, Posturology & Rehabilitation Journal*, p. 1–7, 30 nov. 2017.

FIELD, Tiffany *et al.* Lower back pain and sleep disturbance are reduced following massage therapy. *Journal of Bodywork and Movement Therapies*, v. 11, n. 2, p. 141–145, abr. 2007.

FINK, Jennifer *et al.* A Quality Brief of an Oncological Multisite Massage and Acupuncture Therapy Program to Improve Cancer-Related Outcomes. *The Journal of Alternative and Complementary Medicine*, v. 26, n. 9, p. 822–826, 1 set. 2020.

FINTA, R *et al.* Does inspiration efficiency influence the stability limits of the trunk in patients with chronic low back pain? *Journal of Rehabilitation Medicine*, p. 0, 2020.

FINTA, Regina; NAGY, Edit; BENDER, Tamás. The effect of diaphragm training on lumbar stabilizer muscles: a new concept for improving segmental stability in the case of low back pain. *Journal of Pain Research*, v. Volume 11, p. 3031–3045, nov. 2018.

FISHER, Laura R. *et al.* Short-term Effects of Thoracic Spine Thrust Manipulation, Exercise, and Education in Individuals With Low Back Pain: A Randomized Controlled Trial. *Journal of Orthopaedic & Sports Physical Therapy*, v. 50, n. 1, p. 24–32, jan. 2020.

FORTIN, Maryse *et al.* The Effects of Combined Motor Control and Isolated Extensor Strengthening versus General Exercise on Paraspinal Muscle Morphology, Composition, and Function in Patients with Chronic Low Back Pain: A Randomized Controlled Trial. *Journal of Clinical Medicine*, v. 12, n. 18, p. 5920, 12 set. 2023.

FOSBERG, Kelly K. *et al.* The Effects of Thrust Joint Manipulation on the Resting and Contraction Thickness of Transversus Abdominis in Patients With Low Back Pain: A Randomized Control Trial. *Journal of Manipulative and Physiological Therapeutics*, v. 43, n. 4, p. 339–355, maio 2020.

FOX, Elisabeth J; MELZACK, Ronald. Transcutaneous electrical stimulation and acupuncture: comparison of treatment for low-back pain. *Pain*, v. 2, n. 2, p. 141–148, 1976.

FREITAS, João Paulo *et al.* One spinal manipulation session reduces local pain sensitivity but does not affect postural stability in individuals with chronic low back pain: a randomised, placebo-controlled trial. *Chiropractic & Manual Therapies*, v. 32, n. 1, p. 20, 31 maio 2024.

FRIEDRICH, M.; CERMAK, T.; MADERBACHER, P. The effect of brochure use versus therapist teaching on patients performing therapeutic exercise and on changes in impairment status. *Physical therapy*, v. 76, n. 10, p. 1082–1088, 1996. Disponível em: <https://pubmed.ncbi.nlm.nih.gov/8863761/>. Acesso em: 8 jun. 2026.

FRIEDRICH, Martin *et al.* Combined exercise and motivation program: Effect on the compliance and level of disability of patients with chronic low back pain: A randomized controlled trial. *Archives of Physical Medicine and Rehabilitation*, v. 79, n. 5, p. 475–487, 1998. Disponível em: <https://pubmed.ncbi.nlm.nih.gov/9596385/>. Acesso em: 8 jun. 2026.

FRITZ, Julie M.; WHITMAN, Julie M.; CHILDS, John D. Lumbar spine segmental mobility assessment: An examination of validity for determining intervention strategies in patients with low back pain. *Archives of Physical Medicine and Rehabilitation*, v. 86, n. 9, p. 1745–1752, set. 2005. Disponível em: <https://pubmed.ncbi.nlm.nih.gov/16181937/>. Acesso em: 8 jun. 2026.

FROST, H. *et al.* Randomised controlled trial for evaluation of fitness programme for patients with chronic low back pain. *BMJ (Clinical research ed.)*, v. 310, n. 6973, p. 151, 21 jan. 1995. Disponível em: <https://pubmed.ncbi.nlm.nih.gov/7833752/>. Acesso em: 8 jun. 2026.

FROST, Helen *et al.* Randomised controlled trial of physiotherapy compared with advice for low back pain. *BMJ (Clinical research ed.)*, v. 329, n. 7468, p. 708–711, 25 set. 2004. Disponível em: <https://pubmed.ncbi.nlm.nih.gov/15377573/>. Acesso em: 8 jun. 2026.

GARAUD, Thomas *et al.* Randomized study of the impact of a therapeutic education program on patients suffering from chronic low-back pain who are treated with transcutaneous electrical nerve stimulation. *Medicine*, v. 97, n. 52, p. e13782, dez. 2018.

GARCIA, Alessandra Narciso *et al.* Identifying Patients With Chronic Low Back Pain Who Respond Best to Mechanical Diagnosis and Therapy: Secondary Analysis of a Randomized Controlled Trial. *Physical Therapy*, v. 96, n. 5, p. 623–630, 1 maio 2016.

GARCIA, Alessandra Narciso *et al.* McKenzie Method of Mechanical Diagnosis and Therapy was slightly more effective than placebo for pain, but not for disability, in patients with chronic non-specific low back pain: a randomised placebo controlled trial with short and longer term follow-up. *British Journal of Sports Medicine*, v. 52, n. 9, p. 594–600, maio 2018.

GARCIA, Laura *et al.* Durability of the Treatment Effects of an 8-Week Self-ADMinistered Home-Based Virtual Reality Program for Chronic Low Back Pain: 6-Month Follow-up Study of a Randomized Clinical Trial. *Journal of Medical Internet Research*, v. 24, n. 5, p. e37480, 25 maio 2022.

GARCIA, Laura M *et al.* An 8-Week Self-ADMinistered At-Home Behavioral Skills-Based Virtual Reality Program for Chronic Low Back Pain: Double-Blind, Randomized, Placebo-Controlled Trial Conducted During COVID-19. *Journal of Medical Internet Research*, v. 23, n. 2, p. e26292, 22 fev. 2021.

GARCIA, Laura M. *et al.* Three-Month Follow-Up Results of a Double-Blind, Randomized Placebo-Controlled Trial of 8-Week Self-ADMinistered At-Home Behavioral Skills-Based Virtual Reality (VR) for Chronic Low Back Pain. *The Journal of Pain*, v. 23, n. 5, p. 822–840, maio 2022.

GARVEY, Timothy A; MARKS, Michael R; WIESEL, Sam W. A prospective, randomized, double-blind evaluation of trigger-point injection therapy for low-back pain. *Spine*, v. 14, n. 9, p. 962–964, 1989.

GEDIN, Filip *et al.* Effectiveness and cost-effectiveness of chiropractic and physiotherapy for chronic low back pain: a multicenter RCT in Sweden. *BMC Musculoskeletal Disorders*, v. 26, n. 1, p. 190, 25 fev. 2025.

GERAGHTY, Adam W A *et al.* Supporting self-management of low back pain with an internet intervention with and without telephone support in primary care (SupportBack 2): a randomised controlled trial of clinical and cost-effectiveness. *The Lancet Rheumatology*, v. 6, n. 7, p. e424–e437, jul. 2024.

GERAGHTY, Adam W A *et al.* Supporting self-management with an internet intervention for low back pain in primary care: a RCT (SupportBack 2). *Health Technology Assessment*, p. 1–90, abr. 2025.

GHADYANI, Leila *et al.* Effectiveness of Multidisciplinary Group-Based Intervention versus Individual Physiotherapy for Improving Chronic Low Back Pain in Nursing Staff: A Clinical Trial with 3- and 6-Month Follow-Up Visits from Tehran, Iran. *Asian Spine Journal*, v. 11, n. 3, p. 396–404, 30 jun. 2017.

GHADYANI, Leila *et al.* Work-Related Low Back Pain Treatment: A Randomized Controlled Trial from Tehran, Iran, Comparing Multidisciplinary Educational Program versus Physiotherapy Education. *Asian Spine Journal*, v. 10, n. 4, p. 690, 2016.

GHOLAMI BORUJENI, Behnam; YALFANI, Ali. Reduction of postural sway in athletes with chronic low back pain through eight weeks of inspiratory muscle training: A randomized controlled trial. *Clinical Biomechanics*, v. 69, p. 215–220, out. 2019.

GHOLAMI-BORUJENI, Behnam; YALFANI, Ali; AHMADNEZHAD, Leila. Eight-Week Inspiratory Muscle Training Alters Electromyography Activity of the Ankle Muscles During Overhead and Single-Leg Squats: A Randomized Controlled Trial. *Journal of Applied Biomechanics*, v. 37, n. 1, p. 13–20, 1 fev. 2021.

GHONAME, El Sayed A. *et al.* Percutaneous Electrical Nerve Stimulation for Low Back Pain: A Randomized Crossover Study. *JAMA*, v. 281, n. 9, p. 818–823, 3 mar. 1999. Disponível em: <https://jamanetwork.com/journals/jama/fullarticle/188949>. Acesso em: 8 jun. 2026.

GHONAME, El-sayed A. *et al.* The effect of stimulus frequency on the analgesic response to percutaneous electrical nerve stimulation in patients with chronic low back pain. *Anesthesia and analgesia*, v. 88, n. 4, p. 841–846, abr. 1999. Disponível em: <https://pubmed.ncbi.nlm.nih.gov/10195535/>. Acesso em: 8 jun. 2026.

GILBERT, J R *et al.* Clinical trial of common treatments for low back pain in family practice. *Br Med J (Clin Res Ed)*, v. 291, n. 6498, p. 791–794, 1985.

GINSBERG, Francis; FAMAEY, Jean Pierre. A double-blind study of topical massage with Rado-Salil® ointment in mechanical low-back pain. *Journal of international medical research*, v. 15, n. 3, p. 148–153, 1987.

GLASER, John A. *et al.* Electrical muscle stimulation as an adjunct to exercise therapy in the treatment of nonacute low back pain: A randomized trial. *Journal of Pain*, v. 2, n. 5, p. 295–300, 2001. Disponível em: <https://pubmed.ncbi.nlm.nih.gov/14622808/>. Acesso em: 8 jun. 2026.

GMÜNDER, R.; KISSLING, Rudolf. [The Efficacy of homeopathy in the treatment of chronic low back pain compared to standardized physiotherapy]. *Zeitschrift fur Orthopadie und ihre Grenzgebiete*, v. 140, n. 5, p. 503–508, set. 2002. Disponível em: <https://pubmed.ncbi.nlm.nih.gov/12226773/>. Acesso em: 8 jun. 2026.

GODFREY, Charles M.; MORGAN, Peter P.; SCHATZKER, Joseph. A randomized trial of manipulation for low-back pain in a medical setting. *Spine*, v. 9, n. 3, p. 301–304, 1984. Disponível em: <https://pubmed.ncbi.nlm.nih.gov/6233718/>. Acesso em: 8 jun. 2026.

GOERTZ, Christine M. *et al.* Effects of spinal manipulation on sensorimotor function in low back pain patients – A randomised controlled trial. *Manual Therapy*, v. 21, p. 183–190, fev. 2016.

GONZÁLEZ, Álvaro Cuñado *et al.* Validation of a sham novel neural mobilization technique in patients with non-specific low back pain: A randomized, placebo-controlled trial. *Musculoskeletal Science and Practice*, v. 53, p. 102378, jun. 2021.

GORJI, Sahar Modares *et al.* Pain Neuroscience Education and Motor Control Exercises versus Core Stability Exercises on Pain, Disability, and Balance in Women with Chronic Low Back Pain. *International Journal of Environmental Research and Public Health*, v. 19, n. 5, p. 2694, 25 fev. 2022.

GRAY, Darryl T. *et al.* Conventional radiography, rapid MR imaging, and conventional MR imaging for low back pain: activity-based costs and reimbursement. *Radiology*, v. 227, n. 3, p. 669–680, 1 jun. 2003. Disponível em: <https://pubmed.ncbi.nlm.nih.gov/12773674/>. Acesso em: 8 jun. 2026.

GREENFIELD, Sheldon *et al.* Nurse-protocol management of low back pain: Outcomes, patient satisfaction and efficiency of primary care. *Western Journal of Medicine*, v. 123, n. 5, p. 350, 1975.

GROESSL, Erik J. *et al.* Cost-effectiveness of Yoga for Chronic Low Back Pain in Veterans. *Medical Care*, v. 58, p. S142–S148, set. 2020.

GUASTALA, Fábio Alexandre Moreschi *et al.* Effect of global postural re-education and isostretching in patients with nonspecific chronic low back pain: a randomized clinical trial. *Fisioterapia em Movimento*, v. 29, n. 3, p. 515–525, set. 2016.

GUÉTIN, S. *et al.* Intérêt de la musicothérapie dans la prise en charge de la lombalgie chronique en milieu hospitalier (Étude contrô lée, randomisée sur 65 patients). *Annales de Readaptation et de Medecine Physique*, v. 48, n. 5, p. 217–224, jun. 2005. Disponível em: <https://pubmed.ncbi.nlm.nih.gov/15914256/>. Acesso em: 8 jun. 2026.

GUL, Sameera *et al.* Effects of instrument assisted soft tissue mobilisation in patients with chronic mechanical low back pain----A randomized control trial. *Journal of the Pakistan Medical Association*, v. 75, n. 02, p. 181–185, 26 jan. 2025.

GUNDEWALL, Brita; LILJEQVIST, Margareta; HANSSON, Tommy. Primary prevention of back symptoms and absence from work. A prospective randomized study among hospital employees. *Spine*, v. 18, n. 5, p. 587–594, 1993. Disponível em: <https://pubmed.ncbi.nlm.nih.gov/8484150/>. Acesso em: 8 jun. 2026.

HAAS, Mitchell *et al.* Chronic disease self-management program for low back pain in the elderly. *Journal of Manipulative and Physiological Therapeutics*, v. 28, n. 4, p. 228–237, 2005. Disponível em: <https://pubmed.ncbi.nlm.nih.gov/15883575/>. Acesso em: 8 jun. 2026.

HADIQA ADNAN *et al.* The effects of a static exercise program verses Swiss ball training for core muscles of the lower back and pelvic region in patients with low back pain after child delivery. a single blind randomized control trial. *Journal of the Pakistan Medical Association*, p. 1–13, 3 fev. 2021.

HAHNE, Andrew J. *et al.* Who Benefits Most From Individualized Physiotherapy or Advice for Low Back Disorders? A Preplanned Effect Modifier Analysis of a Randomized Controlled Trial. *Spine*, v. 42, n. 21, p. E1215–E1224, 1 nov. 2017.

HALDORSEN, E. M.H. *et al.* Predictors for outcome of a multi-modal cognitive behavioural treatment program for low back pain patients-a 12-month follow-up study. *European journal of pain (London, England)*, v. 2, n. 4, p. 293–307, 1998. Disponível em: <https://pubmed.ncbi.nlm.nih.gov/10700325/>. Acesso em: 8 jun. 2026.

HALL, Amanda M. *et al.* Does pain-catastrophising mediate the effect of tai chi on treatment outcomes for people with low back pain? *Complementary Therapies in Medicine*, v. 25, p. 61–66, abr. 2016.

HAMPTON ATKINSON, J. *et al.* A placebo-controlled randomized clinical trial of nortriptyline for chronic low back pain. *Pain*, v. 76, n. 3, p. 287–296, jun. 1998. Disponível em: <https://pubmed.ncbi.nlm.nih.gov/9718247/>. Acesso em: 8 jun. 2026.

HAMZA, Mohamed A. *et al.* Effect of the duration of electrical stimulation on the analgesic response in patients with low back pain. *Anesthesiology*, v. 91, n. 6, p. 1622–1627, 1999. Disponível em: <https://www.ovid.com/jnls/anesthesiology/fulltext/10.1097/00000542-199912000-00012~effect-of-the-duration-of-electrical-stimulation-on-the>. Acesso em: 8 jun. 2026.

HARE-MORTENSEN, Lene; LAURIDSEN, Henrik; GRUNNET-NILSSON, Niels. The relative responsiveness of 3 different types of clinical outcome measures on chiropractic patients with low back pain. *Journal of Manipulative and Physiological Therapeutics*, v. 29, n. 2, p. 95–99, fev. 2006. Disponível em: <https://pubmed.ncbi.nlm.nih.gov/16461167/>. Acesso em: 8 jun. 2026.

HARKAPAA, K. *et al.* A controlled study on the outcome of inpatient and outpatient treatment of low back pain. Part III. Long-term follow-up of pain, disability, and compliance. *Journal of Rehabilitation Medicine*, v. 22, n. 4, p. 181–188, 1990. Disponível em: <https://medicaljournalssweden.se/jrm/article/view/41671>. Acesso em: 8 jun. 2026.

HÄRKÄPÄÄ, Kristiina *et al.* A controlled study on the outcome of inpatient and outpatient treatment of low back pain. Part I. Pain, disability, compliance, and reported treatment benefits three months after treatment. *Journal of Rehabilitation Medicine*, v. 21, n. 2, p. 81–89, 1989.

HÄRKÄPÄÄ, Kristiina *et al.* Health locus of control beliefs and psychological distress as predictors for treatment outcome in low-back pain patients: results of a 3-month follow-up of a controlled intervention study. *Pain*, v. 46, n. 1, p. 35–41, 1991. Disponível em: <https://pubmed.ncbi.nlm.nih.gov/1832753/>. Acesso em: 8 jun. 2026.

HARRISON, Jeremy J. *et al.* Sustained versus repetitive standing trunk extension results in greater spinal growth and pain improvement in back pain: A randomized clinical trial. *Journal of Back and Musculoskeletal Rehabilitation*, v. 37, n. 2, p. 395–405, 14 mar. 2024.

HASANPOUR-DEHKORDI, Ali; DEHGHANI, Arman; SOLATI, Kamal. A comparison of the effects of pilates and mckenzie training on pain and general health in men with chronic low back pain: A randomized trial. *Indian Journal of Palliative Care*, v. 23, n. 1, p. 36, 2017.

HEAPY, Alicia A. *et al.* Interactive Voice Response–Based Self-management for Chronic Back Pain. *JAMA Internal Medicine*, v. 177, n. 6, p. 765, 1 jun. 2017.

HEDLUND, Rune *et al.* The long-term outcome of lumbar fusion in the Swedish lumbar spine study. *The Spine Journal*, v. 16, n. 5, p. 579–587, maio 2016.

HELMHOUT, P. H. *et al.* Comparison of a high-intensity and a low-intensity lumbar extensor training program as minimal intervention treatment in low back pain: a randomized trial. *European spine journal : official publication of the European Spine Society, the European Spinal Deformity Society, and the European Section of the Cervical Spine Research Society*, v. 13, n. 6, p. 537–547, out. 2004. Disponível em: <https://pubmed.ncbi.nlm.nih.gov/15095072/>. Acesso em: 8 jun. 2026.

HELMHOUT, Pieter H. *et al.* Rationale and design of a multicenter randomized controlled trial on a "minimal intervention" in Dutch army personnel with nonspecific low back pain [ISRCTN19334317]. *BMC musculoskeletal disorders*, v. 5, n. 1, 9 nov. 2004. Disponível em: <https://pubmed.ncbi.nlm.nih.gov/15535881/>. Acesso em: 8 jun. 2026.

HERNANDEZ-LUCAS, Pablo *et al.* Effects of a back school-based intervention on non-specific low back pain in adults: a randomized controlled trial. *BMC Complementary Medicine and Therapies*, v. 23, n. 1, p. 229, 10 jul. 2023.

HERTZMAN-MILLER, Ruth P. *et al.* Comparing the Satisfaction of Low Back Pain Patients Randomized to Receive Medical or Chiropractic Care: Results From the UCLA Low-Back Pain Study. *American Journal of Public Health*, v. 92, n. 10, p. 1628–1633, out. 2002.

HICKS, Gregory E. *et al.* Preliminary development of a clinical prediction rule for determining which patients with low back pain will respond to a stabilization exercise program. *Archives of Physical Medicine and Rehabilitation*, v. 86, n. 9, p. 1753–1762, set. 2005. Disponível em: <https://pubmed.ncbi.nlm.nih.gov/16181938/>. Acesso em: 8 jun. 2026.

HLOBIL, Hynek *et al.* The Effects of a Graded Activity Intervention for Low Back Pain in Occupational Health on Sick Leave, Functional Status and Pain: 12-Month Results of a Randomized Controlled Trial. *Journal of Occupational Rehabilitation 2005 15:4*, v. 15, n. 4, p. 569–580, dez. 2005. Disponível em: <https://link.springer.com/article/10.1007/s10926-005-8035-y>. Acesso em: 8 jun. 2026.

HODGES, Paul William *et al.* Effect of a Consumer-Focused Website for Low Back Pain on Health Literacy, Treatment Choices, and Clinical Outcomes: Randomized Controlled Trial. *Journal of Medical Internet Research*, v. 23, n. 6, p. e27860, 15 jun. 2021.

HOEIJENBOS, Margreet *et al.* Cost-effectiveness of an active implementation strategy for the Dutch physiotherapy guideline for low back pain. *Health Policy*, v. 75, n. 1, p. 85–98, dez. 2005. Disponível em: <https://pubmed.ncbi.nlm.nih.gov/16298231/>. Acesso em: 8 jun. 2026.

HONG, Mindy *et al.* Effectiveness of Hybrid Form Impulse Therapy (HFIT) Compared to Traditional Transcutaneous Electronic Nerve Stimulation (TENS) in Patients with Chronic Low Back and Knee Pain: A Randomized Controlled Trial. *Journal of Pain Research*, v. Volume 17, p. 2417–2430, jul. 2024.

HOOKER, Quenten L. *et al.* Motor skill training versus strength and flexibility exercise in people with chronic low back pain: Preplanned analysis of effects on kinematics during a functional activity. *Clinical Biomechanics*, v. 92, p. 105570, fev. 2022.

HSIEH, Lisa Li Chen *et al.* A randomized controlled clinical trial for low back pain treated by acupressure and physical therapy. *Preventive Medicine*, v. 39, n. 1, p. 168–176, 1 jul. 2004. Disponível em: <https://www.sciencedirect.com/science/article/abs/pii/S009174350400057X>. Acesso em: 8 jun. 2026.

HSIEH, Lisa Li Chen *et al.* Treatment of low back pain by acupressure and physical therapy: randomised controlled trial. *BMJ (Clinical research ed.)*, v. 332, n. 7543, p. 696–698, 25 mar. 2006. Disponível em: <https://pubmed.ncbi.nlm.nih.gov/16488895/>. Acesso em: 8 jun. 2026.

HSIEH, Ru Lan; LEE, Wen Chung. One-shot percutaneous electrical nerve stimulation vs. transcutaneous electrical nerve stimulation for low back pain: comparison of therapeutic effects. *American journal of physical medicine & rehabilitation*, v. 81, n. 11, p. 838–843, 1 nov. 2002. Disponível em: <https://pubmed.ncbi.nlm.nih.gov/12394995/>. Acesso em: 8 jun. 2026.

HUANG, Huanjie *et al.* Effects of dynamic neuromuscular stabilization training on the core muscle contractility and standing postural control in patients with chronic low back pain: a randomized controlled trial. *BMC Musculoskeletal Disorders*, v. 26, n. 1, p. 213, 1 mar. 2025.

HUANG, Wen-Hung; TANG, Chun-Kai; SHIH, Yi-Fen. Effectiveness of gluteal control training in chronic low back pain patients with functional leg length inequality. *Scientific Reports*, v. 14, n. 1, p. 24432, 18 out. 2024.

HUBLEY-KOZEY, Cheryl L.; VEZINA, M. Johanne. Muscle activation during exercises to improve trunk stability in men with low back pain. *Archives of Physical Medicine and Rehabilitation*, v. 83, n. 8, p. 1100–1108, 2002. Disponível em: <https://pubmed.ncbi.nlm.nih.gov/12161831/>. Acesso em: 8 jun. 2026.

HÜGLI, Anna Sabrina *et al.* Adherence to home exercises in non-specific low back pain. A randomised controlled pilot trial. *Journal of Bodywork and Movement Therapies*, v. 19, n. 1, p. 177–185, jan. 2015.

HURRI, H. The Swedish back school in chronic low back pain. Part I. Benefits. *Scandinavian journal of rehabilitation medicine*, v. 21, n. 1, p. 33–40, 1989.

HURWITZ, Eric L. *et al.* A randomized trial of medical care with and without physical therapy and chiropractic care with and without physical modalities for patients with low back pain: 6-month follow-up outcomes from the UCLA low back pain study. *Spine*, v. 27, n. 20, p. 2193–2204, 15 out. 2002. Disponível em: <https://pubmed.ncbi.nlm.nih.gov/12394892/>. Acesso em: 8 jun. 2026.

HUSSEIN, Hisham Mohamed; MORSI, Amal Ahmed; ABDELRAOOF, Neveen Abdellatif. The immediate effect of sustained natural apophyseal glide on postural stability and pain in individuals presenting with flexion-dominant chronic low back pain: A randomized single-blinded placebo-controlled trial. *Journal of Back and Musculoskeletal Rehabilitation*, v. 34, n. 6, p. 1079–1086, 3 nov. 2021.

HUSSIEN, Hisham Mohamed *et al.* Effect of Mulligan Concept Lumbar SNAG on Chronic Nonspecific Low Back Pain. *Journal of Chiropractic Medicine*, v. 16, n. 2, p. 94–102, jun. 2017.

IBRAHIM, Adel M. *et al.* Effect of Maitland mobilization on lumbar proprioception, pain, and disability in patients with chronic nonspecific low back pain. *Physiotherapy Quarterly*, v. 31, n. 4, p. 87–92, 27 mar. 2023.

IBRAHIM, Aminu A.; AKINDELE, Mukadas O.; GANIYU, Sokunbi O. Effectiveness of patient education plus motor control exercise versus patient education alone versus motor control exercise alone for rural community-dwelling adults with chronic low back pain: a randomised clinical trial. *BMC Musculoskeletal Disorders*, v. 24, n. 1, p. 142, 23 fev. 2023.

IBRAHIMI GHAVAMABADI, Leila *et al.* Effectiveness of a training program on the low back pain and functional disability in industrial workers. *International Journal of Health Promotion and Education*, v. 60, n. 6, p. 343–353, 2 nov. 2022.

IDOWU, Opeyemi Ayodiipo *et al.* Graded activity with and without daily-monitored-walking in patients with type 2 diabetes with low back pain: secondary analysis of a randomized-clinical trial. *Archives of Physiotherapy*, v. 11, n. 1, p. 10, 15 dez. 2021.

IN, Tae-Sung *et al.* Effects of the Multidimensional Treatment on Pain, Disability, and Sitting Posture in Patients with Low Back Pain: A Randomized Controlled Trial. *Pain Research and Management*, v. 2021, p. 1–8, 30 jun. 2021.

INYOUNG, Kong. The effect of the diaphragm stretching technique in the diaphragm contraction rate and trunk muscle activity in back pain patients. *Journal of Back and Musculoskeletal Rehabilitation*, v. 37, n. 1, p. 225–231, 5 jan. 2024.

IOSUB, Monica Elena; TIRLA, Sebastian; LAZAR, Liviu. Impact of Vojta therapy combined with standard care on psychometric and functional parameters in patients with chronic lower back pain: a randomized controlled trial. *Journal of Medicine and Life*, v. 17, n. 5, p. 478–485, maio 2024.

IQRA NAYYAB *et al.* The effects of an exercise program for core muscle strengthening in patients with low back pain after cesarean-section: a single blind randomized controlled trial. *Journal of the Pakistan Medical Association*, p. 1–15, 9 fev. 2021.

ITOH, Kazunori *et al.* Effects of trigger point acupuncture on chronic low back pain in elderly patients--a sham-controlled randomised trial. *Acupuncture in medicine : journal of the British Medical Acupuncture Society*, v. 24, n. 1, p. 5–12, 2006. Disponível em: <https://pubmed.ncbi.nlm.nih.gov/16618043/>. Acesso em: 8 jun. 2026.

ITOH, Kazunori; KATSUMI, Yasukazu; KITAKOJI, Hiroshi. Trigger point acupuncture treatment of chronic low back pain in elderly patients--a blinded RCT. *Acupuncture in medicine : journal of the British Medical Acupuncture Society*, v. 22, n. 4, p. 170–177, 2004. Disponível em: <https://pubmed.ncbi.nlm.nih.gov/15628774/>. Acesso em: 8 jun. 2026.

ITOH, Naohiro *et al.* Evaluation of the Effect of Patient Education and Strengthening Exercise Therapy Using a Mobile Messaging App on Work Productivity in Japanese Patients With Chronic Low Back Pain: Open-Label, Randomized, Parallel-Group Trial. *JMIR mHealth and uHealth*, v. 10, n. 5, p. e35867, 16 maio 2022.

JACOBS, Jesse V. *et al.* Effects of low back pain and of stabilization or movement-system-impairment treatments on induced postural responses: A planned secondary analysis of a randomised controlled trial. *Manual Therapy*, v. 21, p. 210–219, fev. 2016.

JASSI, Fabrício José *et al.* Star-Shape Kinesio Taping Is Not Better Than a Minimal Intervention or Sham Kinesio Taping for Pain Intensity and Postural Control in Chronic Low Back Pain: A Randomized Controlled Trial. *Archives of Physical Medicine and Rehabilitation*, v. 102, n. 7, p. 1352- 1360.e3, jul. 2021.

JENSEN, Lone Donbaek *et al.* Prevention of low back pain in female eldercare workers: Randomized controlled work site trial. *Spine*, v. 31, n. 16, p. 1761–1769, jul. 2006. Disponível em: <https://journals.lww.com/spinejournal/fulltext/2006/07150/prevention_of_low_back_pain_in_female_eldercare.2.aspx>. Acesso em: 8 jun. 2026.

JEONG, Ui-Cheol *et al.* The effects of gluteus muscle strengthening exercise and lumbar stabilization exercise on lumbar muscle strength and balance in chronic low back pain patients. *Journal of Physical Therapy Science*, v. 27, n. 12, p. 3813–3816, 2015.

JEONG, Ui-Cheol *et al.* The effects of self-mobilization techniques for the sciatic nerves on physical functions and health of low back pain patients with lower limb radiating pain. *Journal of Physical Therapy Science*, v. 28, n. 1, p. 46–50, 2016.

JONES, Susan L.; JONES, Paul K.; KATZ, Janet. Compliance for low-back pain patients in the emergency department. A randomized trial. *Spine*, v. 13, n. 5, p. 553–556, 1988. Disponível em: <https://pubmed.ncbi.nlm.nih.gov/2973132/>. Acesso em: 8 jun. 2026.

JOSHI, Shabnam; YADAV, Rekha; PAWALIA, Alka. Can a cervical muscle inhibition technique reduce chronic low back pain? *Advances in Rehabilitation*, v. 35, n. 4, p. 38–46, 1 dez. 2021.

JOUSSET, Nathalie *et al.* Effects of functional restoration versus 3 hours per week physical therapy: a randomized controlled study. *Spine*, v. 29, n. 5, p. 487–493, 1 mar. 2004. Disponível em: <https://pubmed.ncbi.nlm.nih.gov/15129059/>. Acesso em: 8 jun. 2026.

JUNG, Kyoung-sim *et al.* Influences of Kinesio Taping with Therapeutic Exercise in Patients with Low Back Pain. *Healthcare*, v. 9, n. 8, p. 927, 22 jul. 2021.

JUNG, Kyoung-sim *et al.* The Effectiveness of Trunk Stabilization Exercise Combined with Vibration for Adolescent Patients with Nonspecific Low Back Pain. *International Journal of Environmental Research and Public Health*, v. 17, n. 19, p. 7024, 25 set. 2020.

JUNG, Kyoung-Sim; IN, Tae-Sung. Effects of Interferential Current Treatment on Pain, Functional Ability, and Health-Related Quality of Life in Chronic Stroke Patients with Lumbago; A Randomized Controlled Study. *Journal of The Korean Society of Physical Medicine*, v. 15, n. 1, p. 25–32, 29 fev. 2020.

JURAK, Ivan *et al.* Evaluating the Efficacy of Capacitive Resistive Monopolar Radiofrequency Combined With Proprioceptive Neuromuscular Facilitation in Managing Chronic Low Back Pain: A Randomised Controlled Trial. *Physiotherapy Research International*, v. 30, n. 1, 21 jan. 2025.

K. VISHALI; R.V. VIJAYA KUMAR; VASANTHAN. Effect of Pain Education on Quality of Life of Chronic Low Back Pain Patients. *Indian Journal of Physiotherapy & Occupational Therapy - An International Journal*, v. 16, n. 3, p. 25–31, 9 ago. 2022.

KABAŁA, Tomasz *et al.* Influence of modern technologies used in rehabilitation on selected functional parameters of the spine of patients with low back pain. *Acta of Bioengineering and Biomechanics*, v. 22, p. 1–17, 2020.

KAEDING, T. S. *et al.* Whole-body vibration training as a workplace-based sports activity for employees with chronic low-back pain. *Scandinavian Journal of Medicine & Science in Sports*, v. 27, n. 12, p. 2027–2039, 6 dez. 2017.

KAINZ, Birgit *et al.* [Comparison of three outpatient therapy forms for treatment of chronic low back pain -- findings of a multicentre, cluster randomized study]. *Die Rehabilitation*, v. 45, n. 2, p. 65–77, abr. 2006. Disponível em: <https://pubmed.ncbi.nlm.nih.gov/16575712/>. Acesso em: 8 jun. 2026.

KAŁUŻNA, Anna *et al.* The effect of kinesiotherapy supported by visual biofeedback on a stabilometric platform on health-related quality of life among patients with non-specific low back pain. A randomized, open-label study with a 6-month follow-up. *Medical Research Journal*, v. 8, n. 3, p. 216–225, 29 set. 2023.

KAMALI, Fahimeh *et al.* Comparison of manipulation and stabilization exercises in patients with sacroiliac joint dysfunction patients: A randomized clinical trial. *Journal of Bodywork and Movement Therapies*, v. 23, n. 1, p. 177–182, jan. 2019.

KANAAN, Saddam F. *et al.* Evaluating the effectiveness of a comprehensive education on low back pain treatment outcomes: A controlled clinical study. *Clinical Rehabilitation*, v. 37, n. 1, p. 98–108, 7 jan. 2023.

KANG, Jeong-Il; JEONG, Dae-Keun; CHOI, Hyun. Effect of exhalation exercise on trunk muscle activity and oswestry disability index of patients with chronic low back pain. *Journal of Physical Therapy Science*, v. 28, n. 6, p. 1738–1742, 2016.

KANG, Tae-Woo *et al.* Effect of 6-week lumbar stabilization exercise performed on stable versus unstable surfaces in automobile assembly workers with mechanical chronic low back pain. *Work*, v. 60, n. 3, p. 445–454, 31 jul. 2018.

KANKAANPÄÄ, Markku *et al.* The efficacy of active rehabilitation in chronic low back pain. Effect on pain intensity, self-experienced disability, and lumbar fatigability. *Spine*, v. 24, n. 10, p. 1034–1042, 15 maio 1999. Disponível em: <https://pubmed.ncbi.nlm.nih.gov/10332798/>. Acesso em: 8 jun. 2026.

KANLI, Kansu. Investigation of the effects of different treatment approaches on lumbar stabilizer muscles and diaphragm motility in individuals with chronic low back pain. *Turkish Journal of Physical Medicine and Rehabilitation*, v. 70, n. 3, p. 358–369, 26 ago. 2024.

KAPLAN, Şeyhmus *et al.* Short-Term Effects of Kinesio Taping in Women with Pregnancy-Related Low Back Pain: A Randomized Controlled Clinical Trial. *Medical Science Monitor*, v. 22, p. 1297–1301, 18 abr. 2016.

KARADUMAN, Ceren; ATAŞ BALCI, Leyla. The effects of in-person-supervised, tele-supervised, and unsupervised stabilization exercises on pain, functionality, and kinesiophobia in patients with chronic low back pain: a randomized, single-blind trial. *Physiotherapy Theory and Practice*, v. 40, n. 11, p. 2492–2502, 30 nov. 2024.

KARARTI, Caner *et al.* The effects of dynamic neuromuscular stabilization approach on clinical outcomes in older patients with chronic nonspecific low back pain: a randomized, controlled clinical trial. *Somatosensory & Motor Research*, v. 40, n. 3, p. 116–125, 3 jul. 2023.

KARASEL, Seide; ONCEL, Sema; SONMEZ, Ipek. The Effect of Short-Wave Diathermy and Exercise on Depressive Affect in Chronic Low Back Pain Patients. *Medical Archives*, v. 75, n. 3, p. 216, 2021.

KAROUI, Souad *et al.* Evaluation de la force musculaire chez le lombalgique chronique: Comparaison entre reeducation isocinetique et reeducation classique. *La Tunisie Médicale*, v. 102, n. 5, 12 maio 2024.

KARP, Jordan F. *et al.* Effect of Problem-Solving Therapy Versus Supportive Management in Older Adults with Low Back Pain and Depression While on Antidepressant Pharmacotherapy. *The American Journal of Geriatric Psychiatry*, v. 26, n. 7, p. 765–777, jul. 2018.

KASIMIS, Konstantinos *et al.* Effects of Manual Therapy Plus Pain Neuroscience Education with Integrated Motivational Interviewing in Individuals with Chronic Non-Specific Low Back Pain: A Randomized Clinical Trial Study. *Medicina*, v. 60, n. 4, p. 556, 29 mar. 2024.

KASIMIS, Konstantinos *et al.* Short-Term Effects of Manual Therapy plus Capacitive and Resistive Electric Transfer Therapy in Individuals with Chronic Non-Specific Low Back Pain: A Randomized Clinical Trial Study. *Medicina*, v. 59, n. 7, p. 1275, 9 jul. 2023.

KAYA, Selçuk; CUG, Mutlu; BEHM, David G. Foam rolling during a simulated half-time attenuates subsequent soccer-specific performance decrements. *Journal of Bodywork and Movement Therapies*, v. 26, p. 193–200, abr. 2021.

KAZEMI, Seyedeh-Somayeh *et al.* The effectiveness of social media and in-person interventions for low back pain conditions in nursing personnel (SMILE). *Nursing Open*, v. 8, n. 3, p. 1220–1231, 15 maio 2021.

KEIJSERS, Jolanda F.E.M. *et al.* A back school in The Netherlands: evaluating the results. *Patient education and counseling*, v. 14, n. 1, p. 31–44, 1989. Disponível em: <https://pubmed.ncbi.nlm.nih.gov/10313784/>. Acesso em: 8 jun. 2026.

KELLER, Anne *et al.* Trunk muscle strength, cross-sectional area, and density in patients with chronic low back pain randomized to lumbar fusion or cognitive intervention and exercises. *Spine*, v. 29, n. 1, p. 3–8, 1 jan. 2004. Disponível em: <https://pubmed.ncbi.nlm.nih.gov/14699268/>. Acesso em: 8 jun. 2026.

KELLER, S. *et al.* Multidisciplinary rehabilitation for chronic back pain in an outpatient setting: A controlled randomized trial. *European Journal of Pain*, v. 1, n. 4, p. 279–292, 1 dez. 1997. Disponível em: </doi/pdf/10.1016/S1090-3801%2897%2990037-9>. Acesso em: 8 jun. 2026.

KENDRICK, D. *et al.* Radiography of the lumbar spine in primary care patients with low back pain: randomised controlled trial. *BMJ (Clinical research ed.)*, v. 322, n. 7283, p. 400–405, 17 fev. 2001. Disponível em: <https://pubmed.ncbi.nlm.nih.gov/11179160/>. Acesso em: 8 jun. 2026.

KERR, Daniel P.; WALSH, Deirdre M.; BAXTER, David. Acupuncture in the management of chronic low back pain: a blinded randomized controlled trial. *The Clinical journal of pain*, v. 19, n. 6, p. 364–370, 2003. Disponível em: <https://pubmed.ncbi.nlm.nih.gov/14600536/>. Acesso em: 8 jun. 2026.

KHALEDI, Arash; GHEITASI, Mehdi. Isometric vs Isotonic Core Stabilization Exercises to Improve Pain and Disability in Patients with Non-specific Chronic Low Back Pain: A Randomized Controlled Trial. *Anesthesiology and Pain Medicine*, v. 14, n. 1, 15 fev. 2024.

KHALIL, Tarek M. *et al.* Stretching in the rehabilitation of low-back pain patients. *Spine*, v. 17, n. 3, p. 311–317, 1992. Disponível em: <https://pubmed.ncbi.nlm.nih.gov/1533060/>. Acesso em: 8 jun. 2026.

KHAN, Muhammad *et al.* The effects of lumbar stabilization exercises with and without jaw movements in non-specific low back pain (A randomized controlled trial). *Pakistan Journal of Medical Sciences*, v. 40, n. 6, 21 maio 2024.

KHODADAD, Behrouz *et al.* Comparing the Effectiveness of Cognitive Functional Treatment and Lumbar Stabilization Treatment on Pain and Movement Control in Patients With Low Back Pain. *Sports Health: A Multidisciplinary Approach*, v. 12, n. 3, p. 289–295, 16 maio 2020.

KHOLOOSY, Leyla *et al.* Evaluation of the Therapeutic Effect of Low Level Laser in Controlling Low Back Pain: A Randomized Controlled Trial. *Journal of Lasers in Medical Sciences*, v. 11, n. 2, p. 120–125, 15 mar. 2020.

KHUSHBOO KUIYA; SNEHA JOSHI; VIJAY KAUSHIK. To Determine the Effect of Iliopsoas MET along with the Conventional Therapy on Pain, Functional Disability and lumbar lordosis in the Patients with Non-Specific Low Back Pain. *Indian Journal of Physiotherapy and Occupational Therapy - An International Journal*, v. 17, n. 1, p. 22–26, 16 jan. 2023.

KİANİ, Nida *et al.* Cupping therapy combined with conventional physical therapy improves pain and health related quality of life among female patients with low back pain. *Journal of Experimental and Clinical Medicine*, v. 39, n. 4, p. 948–953, 29 out. 2022.

KİBAR, Sibel *et al.* The Effectiveness of Combined Transcutaneous Electrical Nerve Stimulation and Interferential Current Therapy on Chronic Low Back Pain: A Randomized, Double-Blind, Sham-Controlled Study. *Fiziksel Tıp ve Rehabilitasyon Bilimleri Dergisi*, v. 23, n. 1, p. 32–40, 2020.

KIM, Beomryong; YIM, Jongeun. Core Stability and Hip Exercises Improve Physical Function and Activity in Patients with Non-Specific Low Back Pain: A Randomized Controlled Trial. *The Tohoku Journal of Experimental Medicine*, v. 251, n. 3, p. 193–206, 2020.

KIM, Byungho J. *et al.* Manipulative rehabilitation applied soon after lumbar disc surgery improves late post-operative functional disability: A preliminary 2-year follow-up study. *Journal of Back and Musculoskeletal Rehabilitation*, v. 30, n. 5, p. 999–1004, 22 set. 2017.

KIM, Heejae *et al.* Effect of Whole Body Horizontal Vibration Exercise in Chronic Low Back Pain Patients: Vertical Versus Horizontal Vibration Exercise. *Annals of Rehabilitation Medicine*, v. 42, n. 6, p. 804–813, 31 dez. 2018.

KIM, Jae-Hong *et al.* Efficacy of invasive laser acupuncture in treating chronic non-specific low back pain: A randomized controlled trial. *PLOS ONE*, v. 17, n. 5, p. e0269282, 31 maio 2022.

KIM, Ji-Hwan; PARK, Hun-Young. Effects of combined hip exercise and passive stretching on muscle stiffness, pain perception and painrelated disability, and physical function in older adults with low back pain. *Physical Activity and Nutrition*, v. 26, n. 3, p. 016–024, 30 set. 2022.

KIM, Seong-Kyong; KIM, Hee-Seung; CHUNG, Sung-Soo. Effects of an Individualized Educational Program for Korean Patients With Chronic Low Back Pain: A Randomized Controlled Trial. *Journal of Nursing Research*, v. 29, n. 6, p. e177, dez. 2021.

KIM, Seung-Kook *et al.* Clinical outcomes and cost-effectiveness of massage chair therapy versus basic physiotherapy in lower back pain patients. *Medicine*, v. 99, n. 12, p. e19514, mar. 2020.

KIM, Seungmin *et al.* Immediate effect of sling exercises combined with mechanic vibration and oscillation mobilization in chronic nonspecific low back pain patients: a randomized controlled trial. *Disability and Rehabilitation*, v. 46, n. 1, p. 40–47, 2 jan. 2024.

KIM, Soo Kyoung; PARK, Hyojung. The Effect of Auricular Acupressure for Chronic Low Back Pain in Elders. *Holistic Nursing Practice*, v. 35, n. 4, p. 182–190, jul. 2021.

KIM, Soochul; JEE, Yongseok. Effects of 3D Moving Platform Exercise on Physiological Parameters and Pain in Patients with Chronic Low Back Pain. *Medicina*, v. 56, n. 7, p. 351, 15 jul. 2020.

KIM, TaeYeong *et al.* Effectiveness of Simulated Horseback Riding for Patients With Chronic Low Back Pain: A Randomized Controlled Trial. *Journal of Sport Rehabilitation*, v. 29, n. 2, p. 179–185, 1 fev. 2020.

KIM, Young Kyun; CHO, Sung Yeon; LEE, Kun Ho. Effects of transcutaneous electrical nerve stimulation and instrument-assisted soft tissue mobilization combined treatment on chronic low back pain: A randomized controlled trial. *Journal of Back and Musculoskeletal Rehabilitation*, v. 34, n. 5, p. 895–902, 6 set. 2021.

KLADNY, Bernd; FISCHER, F. C.; HAASE, I. [Evaluation of specific stabilizing exercise in the treatment of low back pain and lumbar disk disease in outpatient rehabilitation]. *Zeitschrift fur Orthopadie und ihre Grenzgebiete*, v. 141, n. 4, p. 401–405, jul. 2003. Disponível em: <https://pubmed.ncbi.nlm.nih.gov/12928996/>. Acesso em: 8 jun. 2026.

KLEIN, R. G.; EEK, B. C. Low-energy laser treatment and exercise for chronic low back pain: double-blind controlled trial. *Archives of Physical Medicine and Rehabilitation*, v. 71, n. 1, p. 34–37, 1 jan. 1990. Disponível em: <https://europepmc.org/article/med/2136991>. Acesso em: 8 jun. 2026.

KO, Jong Hyeon *et al.* Effect of an 8-week lumbar stabilization and lower extremity strength exercise on lumbar pain and physical function in middle-aged women with chronic back pain. *Physical Activity and Nutrition*, v. 28, n. 4, p. 001–008, 31 dez. 2024.

KO, Kwang-Jun *et al.* Effects of 12-week lumbar stabilization exercise and sling exercise on lumbosacral region angle, lumbar muscle strength, and pain scale of patients with chronic low back pain. *Journal of Physical Therapy Science*, v. 30, n. 1, p. 18–22, 2018.

KOKIC, I *et al.* Effect of therapeutic exercises on pregnancy-related low back pain and pelvic girdle pain: Secondary analysis of a randomized controlled trial. *Journal of Rehabilitation Medicine*, v. 49, n. 3, p. 251–257, 2017.

KOLU, Päivi *et al.* Neuromuscular exercise and counseling for treating recurrent low back pain in female healthcare workers—Findings from a 24-month follow-up study of a randomized controlled trial. *Scandinavian Journal of Medicine & Science in Sports*, v. 33, n. 11, p. 2239–2249, 19 nov. 2023.

KOMINSKI, Gerald F. *et al.* Economic evaluation of four treatments for low-back pain: results from a randomized controlled trial. *Medical care*, v. 43, n. 5, p. 428–435, maio 2005. Disponível em: <https://pubmed.ncbi.nlm.nih.gov/15838406/>. Acesso em: 8 jun. 2026.

KONG, Jiang-Ti *et al.* Effect of Electroacupuncture vs Sham Treatment on Change in Pain Severity Among Adults With Chronic Low Back Pain. *JAMA Network Open*, v. 3, n. 10, p. e2022787, 27 out. 2020.

KONG, Pui Wah *et al.* Functional versus conventional strength and conditioning programs for back injury prevention in emergency responders. *Frontiers in Bioengineering and Biotechnology*, v. 10, 9 set. 2022.

KOSIĆ, Marko; MALNAR, Daniela; LEKIĆ, Andrica. A Study of 60 Patients with Low Back Pain to Compare Outcomes Following Magnetotherapy, Ultrasound, Laser, and Electrotherapy with and without Lumbosacral Kinesiotherapy. *Medical Science Monitor*, v. 30, 23 fev. 2024.

KOUMANTAKIS, George A.; WATSON, Paul J.; OLDHAM, Jacqueline A. Supplementation of general endurance exercise with stabilisation training versus general exercise only: Physiological and functional outcomes of a randomised controlled trial of patients with recurrent low back pain. *Clinical Biomechanics*, v. 20, n. 5, p. 474–482, 2005. Disponível em: <https://pubmed.ncbi.nlm.nih.gov/15836934/>. Acesso em: 8 jun. 2026.

KOUTARAPU, Sunita; GHUMARE, Dipti. Proprioceptive Neuromuscular Facilitation Exercises Versus Lumbar Stabilization Exercises for Chronic Low Back Pain Patients. *The Indian Journal of Occupational Therapy*, v. 54, n. 1, p. 23–28, jan. 2022.

KOVACS, Francisco M. *et al.* Effect of firmness of mattress on chronic non-specific low-back pain: Randomised, double-blind, controlled, multicentre trial. *Lancet*, v. 362, n. 9396, p. 1599–1604, 15 nov. 2003. Disponível em: <https://pubmed.ncbi.nlm.nih.gov/14630439/>. Acesso em: 8 jun. 2026.

KOVACS, Francisco M. *et al.* Effectiveness and cost-effectiveness analysis of neuroreflexotherapy for subacute and chronic low back pain in routine general practice: a cluster randomized, controlled trial. *Spine*, v. 27, n. 11, p. 1149–1159, 1 jun. 2002. Disponível em: <https://pubmed.ncbi.nlm.nih.gov/12045510/>. Acesso em: 8 jun. 2026.

KREKOUKIAS, Georgios *et al.* Gait Kinetic and Kinematic Changes in Chronic Low Back Pain Patients and the Effect of Manual Therapy: A Randomized Controlled Trial. *Journal of Clinical Medicine*, v. 10, n. 16, p. 3593, 15 ago. 2021.

KU, Boncho *et al.* Short-Term Efficacy of Pulsed Radiofrequency Thermal Stimulation on Acupoints for Chronic Low Back Pain: A Preliminary Study of a Randomized, Single-Blinded, Placebo-Controlled Trial. *Evidence-Based Complementary and Alternative Medicine*, v. 2018, n. 1, 12 jan. 2018.

KULLICH, Werner *et al.* [Music therapy--effect on pain, sleep and quality of life in low back pain]. *Wiener medizinische Wochenschrift (1946)*, v. 153, n. 9–10, p. 217–221, 2003. Disponível em: <https://pubmed.ncbi.nlm.nih.gov/12836459/>. Acesso em: 8 jun. 2026.

KUMAR, Syal *et al.* Effectiveness of Ayurvedic Massage (*Sahacharadi Taila*) in Patients with Chronic Low Back Pain: A Randomized Controlled Trial. *The Journal of Alternative and Complementary Medicine: Paradigm, Practice, and Policy Advancing Integrative Health*, v. 23, n. 2, p. 109–115, 1 fev. 2017.

KUVAČIĆ, Goran *et al.* Effectiveness of yoga and educational intervention on disability, anxiety, depression, and pain in people with CLBP: A randomized controlled trial. *Complementary Therapies in Clinical Practice*, v. 31, p. 262–267, maio 2018.

KYLLÖNEN, E. S. *et al.* Influence of estrogen-progestin replacement therapy and exercise on lumbar spine mobility and low back symptoms in a healthy early postmenopausal female population: a 2-year randomized controlled trial. *European spine journal : official publication of the European Spine Society, the European Spinal Deformity Society, and the European Section of the Cervical Spine Research Society*, v. 7, n. 5, p. 381–386, 1998. Disponível em: <https://pubmed.ncbi.nlm.nih.gov/9840471/>. Acesso em: 8 jun. 2026.

LANG, Angelica E. *et al.* A randomized controlled trial investigating effects of an individualized pedometer driven walking program on chronic low back pain. *BMC Musculoskeletal Disorders*, v. 22, n. 1, p. 206, 19 dez. 2021.

LANGAGERGAARD, Vivian *et al.* The comparative effects of brief or multidisciplinary intervention on return to work at 1 year in employees on sick leave due to low back pain: A randomized controlled trial. *Clinical Rehabilitation*, v. 35, n. 9, p. 1290–1304, 11 set. 2021.

LAOSEE, Orapin *et al.* The effectiveness of traditional Thai massage versus massage with herbal compress among elderly patients with low back pain: A randomised controlled trial. *Complementary Therapies in Medicine*, v. 48, p. 102253, jan. 2020.

LARA-PALOMO, Inmaculada Carmen *et al.* Effectiveness of monopolar diathermy by radiofrequency combined with exercise in patients with chronic low back pain: A randomized clinical trial. *Journal of Back and Musculoskeletal Rehabilitation*, v. 38, n. 3, p. 420–433, 13 maio 2025.

LARA-PALOMO, Inmaculada Carmen *et al.* Effects of monopolar pulsed-capacitive dielectric radiofrequency diathermy in patients with chronic low back pain: a randomised clinical trial. *Scientific Reports*, v. 14, n. 1, p. 14059, 18 jun. 2024.

LARSEN, I *et al.* Effect of adding lay-tutors to the educational part of a back school programme for patients with subacute, non-specific low back pain: A randomized controlled clinical trial with a two-year follow-up. *Journal of Rehabilitation Medicine*, v. 51, n. 9, p. 698–704, 2019.

LE MOAL, Vincent *et al.* Rehabilitation therapy using the HUBER platform in chronic non-specific low back pain: a randomized controlled trial. *European Journal of Physical and Rehabilitation Medicine*, v. 59, n. 5, nov. 2023.

LEE, Jung Hwan *et al.* A Comparison of the Effect of a 4.4-MHz Radiofrequency Deep Heating Therapy and Ultrasound on Low Back Pain: A Randomized, Double-Blind, Multicenter Trial. *Journal of Clinical Medicine*, v. 11, n. 17, p. 5011, 26 ago. 2022.

LEE, Jung-Seok; KANG, Suh-Jung. The effects of strength exercise and walking on lumbar function, pain level, and body composition in chronic back pain patients. *Journal of Exercise Rehabilitation*, v. 12, n. 5, p. 463–470, 31 out. 2016.

LEEMANS, Lynn *et al.* Transcutaneous electrical nerve stimulation and heat to reduce pain in a chronic low back pain population: a randomized controlled clinical trial. *Brazilian Journal of Physical Therapy*, v. 25, n. 1, p. 86–96, jan. 2021.

LEHMANN, T R; RUSSELL, D W; SPRATT, K F. The impact of patients with nonorganic physical findings on a controlled trial of transcutaneous electrical nerve stimulation and electroacupuncture. *Spine*, v. 8, n. 6, p. 625–634, 1983.

LEHMANN, Thomas R *et al.* Efficacy of electroacupuncture and TENS in the rehabilitation of chronic low back pain patients. *Pain*, v. 26, n. 3, p. 277–290, 1986.

LEIBING, Eric *et al.* Acupuncture treatment of chronic low-back pain - A randomized, blinded, placebo-controlled trial with 9-month follow-up. *Pain*, v. 96, n. 1–2, p. 189–196, 2002. Disponível em: <https://pubmed.ncbi.nlm.nih.gov/11932074/>. Acesso em: 8 jun. 2026.

LENA, Orges *et al.* A randomized controlled trial concerning the implementation of the postural Mézières treatment in elite athletes with low back pain. *Postgraduate Medicine*, v. 134, n. 6, p. 559–572, 18 ago. 2022.

LETAFATKAR, Amir *et al.* The efficacy of a HUBER exercise system mediated sensorimotor training protocol on proprioceptive system, lumbar movement control and quality of life in patients with chronic non-specific low back pain. *Journal of Back and Musculoskeletal Rehabilitation*, v. 30, n. 4, p. 767–778, jul. 2017.

LEWIS, Jeremy S. *et al.* A randomized clinical trial comparing two physiotherapy interventions for chronic low back pain. *Spine*, v. 30, n. 7, p. 711–721, 1 abr. 2005. Disponível em: <https://pubmed.ncbi.nlm.nih.gov/15803071/>. Acesso em: 8 jun. 2026.

LI, Keqiang *et al.* The effect of the Baduanjin exercise on COVID-19-related anxiety, psychological well-being and lower back pain of college students during the pandemic. *BMC Sports Science, Medicine and Rehabilitation*, v. 14, n. 1, p. 102, 8 jun. 2022.

LI, Xin *et al.* Repetitive Transcranial Magnetic Stimulation Combined with Sling Exercise Modulates the Motor Cortex in Patients with Chronic Low Back Pain. *Neuroscience*, v. 545, p. 196–206, maio 2024.

LI, Ying *et al.* The impact of core training combined with breathing exercises on individuals with chronic non-specific low back pain. *Frontiers in Public Health*, v. 13, 21 fev. 2025.

LI, Zhicheng *et al.* The Effect of Virtual Reality Training on Anticipatory Postural Adjustments in Patients with Chronic Nonspecific Low Back Pain: A Preliminary Study. *Neural Plasticity*, v. 2021, p. 1–13, 27 jul. 2021.

LICCIARDONE, John C. *et al.* Osteopathic manipulative treatment for chronic low back pain: a randomized controlled trial. *Spine*, v. 28, n. 13, p. 1355–1362, 1 jul. 2003. Disponível em: <https://pubmed.ncbi.nlm.nih.gov/12838090/>. Acesso em: 8 jun. 2026.

LICCIARDONE, John C.; GATCHEL, Robert J.; ARYAL, Subhash. Recovery From Chronic Low Back Pain After Osteopathic Manipulative Treatment: A Randomized Controlled Trial. *Journal of Osteopathic Medicine*, v. 116, n. 3, p. 144–155, 1 mar. 2016a.

LICCIARDONE, John C.; GATCHEL, Robert J.; ARYAL, Subhash. Targeting Patient Subgroups With Chronic Low Back Pain for Osteopathic Manipulative Treatment: Responder Analyses From a Randomized Controlled Trial. *Journal of Osteopathic Medicine*, v. 116, n. 3, p. 156–168, 1 mar. 2016b.

LIN, Cheng-Feng *et al.* Immediate effects of thoracic mobilization versus soft tissue release on trunk motion, pain, and lumbar muscle activity in patients with chronic low back pain. *Journal of Bodywork and Movement Therapies*, v. 40, p. 1664–1671, out. 2024.

LINTON, Steven J *et al.* The secondary prevention of low back pain: a controlled study with follow-up. *Pain*, v. 36, n. 2, p. 197–207, 1989.

LISI, Anthony J. *et al.* A Pulsed Electromagnetic Field Therapy Device for Non-Specific Low Back Pain: A Pilot Randomized Controlled Trial. *Pain and Therapy*, v. 8, n. 1, p. 133–140, 12 jun. 2019.

LIU, Lizhou *et al.* Acupuncture for chronic low back pain: a randomized controlled feasibility trial comparing treatment session numbers. *Clinical Rehabilitation*, v. 31, n. 12, p. 1592–1603, dez. 2017.

LIU, Mingsheng *et al.* The effect of core stability training combined with fascial release on patients with nonspecific low back pain. *Technology and Health Care*, v. 33, n. 1, p. 257–265, 1 jan. 2025.

LIU, Yixuan *et al.* The effect of blood flow restriction training on core muscle strength and pain in male collegiate athletes with chronic non-specific low back pain. *Frontiers in Public Health*, v. 12, 7 jan. 2025.

LJUNGGREN, Anne Elisabeth *et al.* Effect of exercise on sick leave due to low back pain. A randomized, comparative, long-term study. *Spine*, v. 22, n. 14, p. 1610–1617, 15 jul. 1997. Disponível em: <https://pubmed.ncbi.nlm.nih.gov/9253097/>. Acesso em: 8 jun. 2026.

LONG, Audrey; DONELSON, Ron; FUNG, Tak. Does it matter which exercise? A randomized control trial of exercise for low back pain. *Spine*, v. 29, n. 23, p. 2593–2602, 1 dez. 2004. Disponível em: <https://pubmed.ncbi.nlm.nih.gov/15564907/>. Acesso em: 8 jun. 2026.

LØNN, Jan Harald *et al.* Active back school: prophylactic management for low back pain. A randomized, controlled, 1-year follow-up study. *Spine*, v. 24, n. 9, p. 865–871, 1 maio 1999. Disponível em: <https://pubmed.ncbi.nlm.nih.gov/10327507/>. Acesso em: 8 jun. 2026.

LÓPEZ-MARCOS, José Javier *et al.* The Added Value of Face-to-Face Supervision to a Therapeutic Exercise-Based App in the Management of Patients with Chronic Low Back Pain: A Randomized Clinical Trial. *Sensors*, v. 24, n. 2, p. 567, 16 jan. 2024.

LOUW, Adriaan *et al.* The effect of manual therapy and neuroplasticity education on chronic low back pain: a randomized clinical trial. *Journal of Manual & Manipulative Therapy*, v. 25, n. 5, p. 227–234, 20 out. 2017.

LOVO, Stacey *et al.* Effectiveness of an interprofessional assessment and management approach for people with chronic low back disorders delivered via virtual care: A randomized controlled trial pilot intervention. *DIGITAL HEALTH*, v. 10, 5 jan. 2024.

LUQMAN SULAIMAN, Mohammed *et al.* Exploring Neurokinetic Therapy on Core Muscle Endurance, Lateral Pelvic Tilt, and Quadratus Lumborum Flexibility for subjects with Chronic Non-Specific Low Back Pain. *Muscles, Ligaments and Tendons Journal*, v. 14, n. 4, 31 dez. 2024.

LYTRAS, Dimitrios *et al.* Effects of a tailored mat-Pilates exercise program for older adults on pain, functioning, and balance in women with chronic non-specific low back pain: a randomized controlled trial. *Aging Clinical and Experimental Research*, v. 35, n. 12, p. 3059–3071, 7 nov. 2023.

MA, Qingyu *et al.* Tuina versus physiotherapy or a combination of both for the management of chronic nonspecific low back pain: A randomized controlled trial. *Complementary Therapies in Medicine*, v. 86, p. 103098, nov. 2024.

MACDONALD, A J *et al.* Superficial acupuncture in the relief of chronic low back pain. *Annals of the Royal College of Surgeons of England*, v. 65, n. 1, p. 44, 1983.

MACEDO, Liane Brito *et al.* The influence of Kinesio Taping on muscle fatigue in individuals with low back pain: A randomised controlled trial. *Journal of Back and Musculoskeletal Rehabilitation*, v. 34, n. 4, p. 613–621, 13 jul. 2021.

MADDOX, Todd *et al.* Twelve-month results for a randomized sham-controlled effectiveness trial of an in-home skills-based virtual reality program for chronic low back pain. *PAIN Reports*, v. 9, n. 5, p. e1182, out. 2024.

MAGALHÃES, Maurício Oliveira *et al.* Effectiveness of graded activity versus physiotherapy in patients with chronic nonspecific low back pain: midterm follow up results of a randomized controlled trial. *Brazilian Journal of Physical Therapy*, v. 22, n. 1, p. 82–91, jan. 2018.

MAHMOUDZADEH, Ashraf *et al.* The effect of dry needling on the radiating pain in subjects with discogenic low-back pain: A randomized control trial. *Journal of Research in Medical Sciences*, v. 21, n. 1, jan. 2016.

MANE, Namrata P.; VARADHARAJULU, G.; SHINDE, Sandeep. Effect of Motor Control Training on Isolated Lumbar Stabilizer and Core Muscle Training in Chronic Low Back Pain Patients. *Indian Journal of Public Health Research & Development*, v. 10, n. 7, p. 37, 2019.

MANIKANDAN, P. *et al.* Effectiveness of Pilates exercises on non-specific low back pain to determine pain and disability. *Biomedicine*, v. 41, n. 4, p. 825–829, 31 dez. 2021.

MANNICHE, Claus *et al.* Intensive dynamic back exercises with or without hyperextension in chronic back pain after surgery for lumbar disc protrusion. A clinical trial. *Spine*, v. 18, n. 5, p. 560–567, 1993. Disponível em: <https://pubmed.ncbi.nlm.nih.gov/8484146/>. Acesso em: 8 jun. 2026.

MANNION, Anne F *et al.* 1999 Volvo Award Winner in Clinical Studies: a randomized clinical trial of three active therapies for chronic low back pain. *Spine*, v. 24, n. 23, p. 2435, 1999.

MANNION, Anne F. *et al.* Active therapy for chronic low back pain part 1. Effects on back muscle activation, fatigability, and strength. *Spine*, v. 26, n. 8, p. 897–908, 15 abr. 2001. Disponível em: <https://pubmed.ncbi.nlm.nih.gov/11317112/>. Acesso em: 8 jun. 2026.

MANSELL, Gemma *et al.* Identification of Indirect Effects in a Cognitive Patient Education (COPE) Intervention for Low Back Pain. *Physical Therapy*, v. 97, n. 12, p. 1138–1146, 1 dez. 2017.

MARCHAND, Serge *et al.* Is TENS purely a placebo effect? A controlled study on chronic low back pain. *Pain*, v. 54, n. 1, p. 99–106, 1993. Disponível em: <https://pubmed.ncbi.nlm.nih.gov/8378107/>. Acesso em: 8 jun. 2026.

MARCUZZI, Anna *et al.* Effect of an Artificial Intelligence–Based Self-Management App on Musculoskeletal Health in Patients With Neck and/or Low Back Pain Referred to Specialist Care. *JAMA Network Open*, v. 6, n. 6, p. e2320400, 27 jun. 2023.

MARINI, Mirca *et al.* Low back pain in healthy postmenopausal women and the effect of physical activity: A secondary analysis in a randomized trial. *PLOS ONE*, v. 12, n. 5, p. e0177370, 10 maio 2017.

MARÍN-MÉNDEZ, Honorio *et al.* Using a Robot to Treat Non-specific Low Back Pain: Results From a Two-Arm, Single-Blinded, Randomized Controlled Trial. *Frontiers in Neurorobotics*, v. 15, 14 set. 2021.

MARTIN, Paul R *et al.* Physiotherapy exercises for low back pain: process and clinical outcome. *International Rehabilitation Medicine*, v. 8, n. 1, p. 34–38, 1986.

MARTÍ-SALVADOR, Mireia *et al.* Osteopathic Manipulative Treatment Including Specific Diaphragm Techniques Improves Pain and Disability in Chronic Nonspecific Low Back Pain: A Randomized Trial. *Archives of Physical Medicine and Rehabilitation*, v. 99, n. 9, p. 1720–1729, set. 2018.

MARUGÁN-RUBIO, Daniel *et al.* Effectiveness of Ultrasonography Visual Biofeedback of the Diaphragm in Conjunction with Inspiratory Muscle Training on Muscle Thickness, Respiratory Pressures, Pain, Disability, Quality of Life and Pulmonary Function in Athletes with Non-Specific Low Back Pain: A Randomized Clinical Trial. *Journal of Clinical Medicine*, v. 11, n. 15, p. 4318, 25 jul. 2022.

MAS, Romina Raczy *et al.* Effectiveness of a multidisciplinary BIOPSYCHOSOCIAL intervention for non-specific SUBACUTE low back pain in a working population: a cluster randomized clinical trial. *BMC Health Services Research*, v. 19, n. 1, p. 962, 12 dez. 2019.

MASROOR, Sana *et al.* Effect of Adding Diaphragmatic Breathing Exercises to Core Stabilization Exercises on Pain, Muscle Activity, Disability, and Sleep Quality in Patients With Chronic Low Back Pain: A Randomized Control Trial. *Journal of Chiropractic Medicine*, v. 22, n. 4, p. 275–283, dez. 2023.

MASSÉ-ALARIE, Hugo *et al.* Influence of paravertebral muscles training on brain plasticity and postural control in chronic low back pain. *Scandinavian Journal of Pain*, v. 12, n. 1, p. 74–83, 1 jul. 2016.

MASSÉ-ALARIE, Hugo *et al.* Repetitive peripheral magnetic neurostimulation of multifidus muscles combined with motor training influences spine motor control and chronic low back pain. *Clinical Neurophysiology*, v. 128, n. 3, p. 442–453, mar. 2017.

MATARÁN-PEÑARROCHA, Guillermo A *et al.* Comparison of efficacy of a supervised versus non-supervised physical therapy exercise program on the pain, functionality and quality of life of patients with non-specific chronic low-back pain: a randomized controlled trial. *Clinical Rehabilitation*, v. 34, n. 7, p. 948–959, 10 jul. 2020.

MATHEWS, W; MORKEL, M; MATHEWS, J. Manipulation and traction for lumbago and sciatica: physiotherapeutic techniques used in two controlled trials. *Physiotherapy Practice*, v. 4, n. 4, p. 201–206, 1988.

MAYER, John M. *et al.* Comparison of Supervised and Telehealth Delivery of Worksite Exercise for Prevention of Low Back Pain in Firefighters. *Journal of Occupational & Environmental Medicine*, v. 62, n. 10, p. e586–e592, out. 2020.

MAYER, John M. *et al.* Continuous Low-Level Heat Wrap Therapy for the Prevention and Early Phase Treatment of Delayed-Onset Muscle Soreness of the Low Back: A Randomized Controlled Trial. *Archives of Physical Medicine and Rehabilitation*, v. 87, n. 10, p. 1310–1317, 1 out. 2006. Disponível em: <https://www.sciencedirect.com/science/article/abs/pii/S0003999306008458>. Acesso em: 8 jun. 2026.

MAYER, Tom G. *et al.* A randomized clinical trial of treatment for lumbar segmental rigidity. *Spine*, v. 29, n. 20, p. 2199–2205, 15 out. 2004. Disponível em: <https://pubmed.ncbi.nlm.nih.gov/15480128/>. Acesso em: 8 jun. 2026.

MCCARTHY, Christopher J; POTTER, Louise; OLDHAM, Jackie A. Comparing targeted thrust manipulation with general thrust manipulation in patients with low back pain. A general approach is as effective as a specific one. A randomised controlled trial. *BMJ Open Sport & Exercise Medicine*, v. 5, n. 1, p. e000514, 5 out. 2019.

MCCAULEY, J D *et al.* Hypnosis compared to relaxation in the outpatient management of chronic low back pain. *Archives of Physical Medicine and Rehabilitation*, v. 64, n. 11, p. 548–552, 1983.

MCKEE, M. Diane *et al.* Individual vs. Group Delivery of Acupuncture Therapy for Chronic Musculoskeletal Pain in Urban Primary Care—a Randomized Trial. *Journal of General Internal Medicine*, v. 35, n. 4, p. 1227–1237, 19 abr. 2020.

MEADE, T W *et al.* Comparison of chiropractic and hospital outpatient management of low back pain: a feasibility study. *Journal of Epidemiology and Community Health (1979-)*, p. 12–17, 1986.

MEADE, T. W. *et al.* Low back pain of mechanical origin: randomised comparison of chiropractic and hospital outpatient treatment. *British Medical Journal*, v. 300, n. 6737, p. 1431–1437, 2 jun. 1990. Disponível em: <https://www.bmj.com/content/300/6737/1431>. Acesso em: 8 jun. 2026.

MEADE, T. W. *et al.* Randomised comparison of chiropractic and hospital outpatient management for low back pain: results from extended follow up. *BMJ (Clinical research ed.)*, v. 311, n. 7001, p. 349, 5 ago. 1995. Disponível em: <https://pubmed.ncbi.nlm.nih.gov/7640538/>. Acesso em: 8 jun. 2026.

MEHYAR, Fahed *et al.* Effect of grade III lumbar mobilization on back muscles in chronic low back pain: A randomized controlled trial. *Journal of Allied Health*, v. 49, p. 20–28, fev. 2020.

MELLIN, Guy *et al.* A controlled study on the outcome of inpatient and outpatient treatment of low back pain. Part II. Effects on physical measurements three months after treatment. *Journal of Rehabilitation Medicine*, v. 21, n. 2, p. 91–95, 1989.

MELZACK, R *et al.* Ice massage and transcutaneous electrical stimulation: comparison of treatment for low-back pain. *Pain*, v. 9, n. 2, p. 209–217, 1980.

MELZACK, Ronald; VETERE, Phyllis; FINCH, Lois. Transcutaneous electrical nerve stimulation for low back pain: a comparison of TENS and massage for pain and range of motion. *Physical Therapy*, v. 63, n. 4, p. 489–493, 1983.

MERLO, Jeanne Karlette *et al.* Effects of a Mat Pilates Exercise Program Associated with Photobiomodulation Therapy in Patients with Chronic Nonspecific Low Back Pain: A Randomized, Double-Blind, Sham-Controlled Trial. *Healthcare*, v. 12, n. 14, p. 1416, 16 jul. 2024.

METİN ÖKMEN, Burcu *et al.* The effects of the number of physical therapy sessions on pain, disability,and quality of life in patients with chronic low back pain. *TURKISH JOURNAL OF MEDICAL SCIENCES*, v. 47, p. 1425–1431, 2017.

MICHALSEN, Andreas *et al.* Effectiveness of focused meditation for patients with chronic low back pain—A randomized controlled clinical trial. *Complementary Therapies in Medicine*, v. 26, p. 79–84, jun. 2016.

MILLER, Eric R. *et al.* A Comparison of the McKenzie Approach to a Specific Spine Stabilization Program for Chronic Low Back Pain. *Journal of Manual & Manipulative Therapy*, v. 13, n. 2, p. 103–112, 2005. Disponível em: <https://www.tandfonline.com/doi/abs/10.1179/106698105790824996>. Acesso em: 8 jun. 2026.

MILLER, Paul *et al.* Cost-effectiveness of lumbar spine radiography in primary care patients with low back pain. *Spine*, v. 27, n. 20, p. 2291–2297, 15 out. 2002. Disponível em: <https://pubmed.ncbi.nlm.nih.gov/12394910/>. Acesso em: 8 jun. 2026.

MINAKAWA, Yoichi *et al.* Trigger Point Acupuncture and Exercise for Chronic Low Back Pain in Older Adult: a Preliminary Randomized Clinical Trial. *Journal of Acupuncture and Meridian Studies*, v. 15, n. 2, p. 143–151, 30 abr. 2022.

MINETTO, M.A. *et al.* Effectiveness of an innovative mattress overlay for improving rehabilitation in low back pain: A pilot randomized controlled study. *Journal of Back and Musculoskeletal Rehabilitation*, v. 31, n. 6, p. 1075–1083, 28 nov. 2018.

MITCHELL, Robert I.; CARMEN, Glenn M. The functional restoration approach to the treatment of chronic pain in patients with soft tissue and back injuries. *Spine*, v. 19, n. 6, p. 633–642, 1994. Disponível em: <https://pubmed.ncbi.nlm.nih.gov/8009327/>. Acesso em: 8 jun. 2026.

MIYAMOTO, Gisela Cristiane *et al.* Education With Therapeutic Alliance Did Not Improve Symptoms in Patients With Chronic Low Back Pain and Low Risk of Poor Prognosis Compared to Education Without Therapeutic Alliance: A Randomized Controlled Trial. *Journal of Orthopaedic & Sports Physical Therapy*, v. 51, n. 8, p. 392–400, ago. 2021.

MOFFETT, J A Klaber *et al.* A controlled, prospective study to evaluate the effectiveness of a back school in the relief of chronic low back pain. *Spine*, v. 11, n. 2, p. 120–122, 1986.

MOFFETT, J. K. *et al.* Randomised controlled trial of exercise for low back pain: clinical outcomes, costs, and preferences. *BMJ*, v. 319, n. 7205, p. 279–283, 31 jul. 1999.

MOHAMMADI, Vahid; LETAFATKAR, Amir; JAFARNEZHADGERO, Amir Ali. Effects of Core Stability Training on Kinematic and Kinetic Variables in Patients With Chronic Low Back Pain. *Physical Treatments - Specific Physical Therapy Journal*, v. 13, n. 1, p. 55–66, 1 jan. 2023.

MOLDE HAGEN, Eli; GRASDAL, Astrid; ERIKSEN, Hege R. Does Early Intervention With a Light Mobilization Program Reduce Long-Term Sick Leave for Low Back Pain: A 3-Year Follow-up Study. *Spine*, v. 28, n. 20, p. 2309–2315, out. 2003.

MOLSBERGER, Albrecht F. *et al.* Does acupuncture improve the orthopedic management of chronic low back pain--a randomized, blinded, controlled trial with 3 months follow up. *Pain*, v. 99, n. 3, p. 579–587, 2002. Disponível em: <https://pubmed.ncbi.nlm.nih.gov/12406534/>. Acesso em: 8 jun. 2026.

MOREIRA, Roberta F. C. *et al.* Effects of a workplace exercise program on physical capacity and lower back symptoms in hospital nursing assistants: a randomized controlled trial. *International Archives of Occupational and Environmental Health*, v. 94, n. 2, p. 275–284, 16 fev. 2021.

MORRIS, Craig E.; BONNEFIN, Debra; DARVILLE, Caroline. The Torsional Upper Crossed Syndrome: A multi-planar update to Janda's model, with a case series introduction of the mid-pectoral fascial lesion as an associated etiological factor. *Journal of Bodywork and Movement Therapies*, v. 19, n. 4, p. 681–689, out. 2015.

MORSØ, Lars *et al.* Effectiveness of stratified treatment for back pain in Danish primary care: A randomized controlled trial. *European Journal of Pain*, v. 25, n. 9, p. 2020–2038, 2 out. 2021.

MOSELEY, Lorimer. Combined physiotherapy and education is efficacious for chronic low back pain. *Australian Journal of Physiotherapy*, v. 48, n. 4, p. 297–302, 2002. Disponível em: <https://pubmed.ncbi.nlm.nih.gov/12443524/>. Acesso em: 8 jun. 2026.

MOSLEMI, Fateme; SAFAVI FAROKHI, Ziaeddin. Effects of electroacupuncture on pain, functional disability and ultrasonographic changes of gluteus maximus muscle in non-specific chronic low back pain patients with gluteus maximus muscle trigger points. *Koomesh journal*, v. 22, n. 4, p. 604–610, 1 set. 2020.

MOUDJARI, Ines *et al.* Evaluation of the Performance of Blueback Physio <math altimg="si1.svg"> <msup> <mrow/> <mrow> <mi mathvariant="normal">®</mi> </mrow> </msup> </math> Medical Device in the Management of Patients Suffering from Chronic Low Back Pain: Randomized Clinical Trial. *IRBM*, v. 46, n. 1, p. 100875, fev. 2025.

MOUHLI, Najla *et al.* Comparison of Mesotherapy and Transcutaneous Electrical Nerve Stimulation (TENS) in the Management of Chronic Non-Specific Low Back Pain: A Randomized Clinical Trial. *La Tunisie Médicale*, v. 103, n. 1, 2 jan. 2025.

MUDD, Emma *et al.* Healthy Lifestyle Care vs Guideline-Based Care for Low Back Pain. *JAMA Network Open*, v. 8, n. 1, p. e2453807, 10 jan. 2025.

MUDDA, Sofia *et al.* Efficacy of Integrated Yoga and Naturopathy With Physiotherapy or Acupuncture for Low Back Pain: A Parallel, Two-Arm, Randomized Trial. *Cureus*, 29 fev. 2024.

MUMTAZ, Sara *et al.* Effect of Core Stability Exercises with Conventional Physiotherapy in Reducing Pain among Patients with Non-Specific Low Back Pain: RCT. *Pakistan Journal of Medical and Health Sciences*, v. 15, n. 10, p. 3063–3065, 30 out. 2021.

NABAVI, Narjess *et al.* The Effect of 2 Different Exercise Programs on Pain Intensity and Muscle Dimensions in Patients With Chronic Low Back Pain: A Randomized Controlled Trial. *Journal of Manipulative and Physiological Therapeutics*, v. 41, n. 2, p. 102–110, fev. 2018.

NAGESWARI, C. *et al.* Effect of Pilates Exercises on Pain, Endurance, Quality-of-Life, and Disability in Postmenopausal Women With Low Back Pain. *Musculoskeletal Care*, v. 23, n. 1, 16 mar. 2025.

NAJAFI GHAGHOLESTANI, Behnoosh *et al.* Effects of Dynamic Neuromuscular Stabilization and Aquatic Exercises on the Pain, Disability, Lumbopelvic Control, and Spinal Posture of Patients With Non-specific Low Back Pain. *Iranian Rehabilitation Journal*, v. 20, n. 3, p. 333–344, 1 nov. 2022.

NAMBI, Gopal *et al.* Clinical and Endocrinological Responses to Different Exercise Training Methods in Chronic Low Back Pain: A Randomized Controlled Trial. *Endocrine, Metabolic & Immune Disorders - Drug Targets*, v. 23, n. 6, p. 801–810, maio 2023.

NAMBI, Gopal; ABDELBASSET, Walid Kamal; ELSAYED, Shereen H.; *et al.* Comparative Effects of Isokinetic Training and Virtual Reality Training on Sports Performances in University Football Players with Chronic Low Back Pain-Randomized Controlled Study. *Evidence-Based Complementary and Alternative Medicine*, v. 2020, n. 1, 16 jan. 2020.

NAMBI, Gopal *et al.* Effects of Virtual Reality Exercises versus Isokinetic Exercises in comparison with Conventional Exercises on the Imaging Findings and Inflammatory Biomarker Changes in Soccer Players with Non-Specific Low Back Pain: A Randomized Controlled Trial. *International Journal of Environmental Research and Public Health*, v. 20, n. 1, p. 524, 28 dez. 2022.

NAMBI, Gopal; ABDELBASSET, Walid Kamal; ALQAHTANI, Bader A.; *et al.* Isokinetic back training is more effective than core stabilization training on pain intensity and sports performances in football players with chronic low back pain. *Medicine*, v. 99, n. 21, p. e20418, 22 maio 2020.

NAMBI, Gopal; ABDELBASSET, Walid Kamal; ALSUBAIE, Saud F.; *et al.* Isokinetic training – its radiographic and inflammatory effects on chronic low back pain. *Medicine*, v. 99, n. 51, p. e23555, 18 dez. 2020.

NAMBI, Gopal *et al.* Short-Term Psychological and Hormonal Effects of Virtual Reality Training on Chronic Low Back Pain in Soccer Players. *Journal of Sport Rehabilitation*, v. 30, n. 6, p. 884–893, 1 ago. 2021.

NARDIN, Daniele Mayumi Kurata *et al.* Effects of photobiomodulation and deep water running in patients with chronic non-specific low back pain: a randomized controlled trial. *Lasers in Medical Science*, v. 37, n. 4, p. 2135–2144, 4 jun. 2022.

NAROUEI, Shideh *et al.* Effects of core stabilization exercises on thickness and activity of trunk and hip muscles in subjects with nonspecific chronic low back pain. *Journal of Bodywork and Movement Therapies*, v. 24, n. 4, p. 138–146, out. 2020.

NAVA-BRINGAS, Tania Inés *et al.* Stabilization Exercises Versus Flexion Exercises in Degenerative Spondylolisthesis: A Randomized Controlled Trial. *Physical Therapy*, v. 101, n. 8, 1 ago. 2021.

NEAMAT ALLAH, Neama *et al.* Effect of repeated application of rigid tape on pain and mobility deficits associated with sacroiliac joint dysfunction. *Journal of Back and Musculoskeletal Rehabilitation*, v. 32, n. 3, p. 487–496, 5 maio 2019.

NEASON, Christopher *et al.* Running is acceptable and efficacious in adults with non-specific chronic low back pain: the ASTEROID randomised controlled trial. *British Journal of Sports Medicine*, v. 59, n. 2, p. 99–108, jan. 2025.

NEWTON, Christopher *et al.* Cognitive Functional Therapy compared with usual physiotherapy care in people with persistent low back pain: a mixed methods feasibility randomised controlled trial in the United Kingdom National Health Service. *Physiotherapy*, v. 123, p. 118–132, jun. 2024.

NEWTON-JOHN, Toby R.O.; SPENCE, Susan H.; SCHOTTE, David. Cognitive-Behavioural Therapy versus EMG Biofeedback in the treatment of chronic low back pain. *Behaviour Research and Therapy*, v. 33, n. 6, p. 691–697, 1995. Disponível em: <https://pubmed.ncbi.nlm.nih.gov/7654161/>. Acesso em: 8 jun. 2026.

NEYAZ, Osama *et al.* Effectiveness of Hatha Yoga Versus Conventional Therapeutic Exercises for Chronic Nonspecific Low-Back Pain. *The Journal of Alternative and Complementary Medicine*, v. 25, n. 9, p. 938–945, 1 set. 2019.

NGO, Oanh Thi Kim; TRINH, Dieu-Thuong Thi; TANG, Wei. Electroacupuncture at Traditional Acupoints or Myofascial Trigger Points for Chronic Nonspecific Low Back Pain: High or Alternated Frequency? A Double-Blinded Randomized Controlled Trial. *Medical Acupuncture*, v. 36, n. 5, p. 250–263, 21 out. 2024.

NGUYEN, Christelle *et al.* Effect of Osteopathic Manipulative Treatment vs Sham Treatment on Activity Limitations in Patients With Nonspecific Subacute and Chronic Low Back Pain. *JAMA Internal Medicine*, v. 181, n. 5, p. 620, 1 maio 2021.

NGUYEN, Christelle *et al.* Intensive spa and exercise therapy program for returning to work for low back pain patients: a randomized controlled trial. *Scientific Reports*, v. 7, n. 1, p. 17956, 20 dez. 2017.

NICHOLAS, Michael K.; WILSON, Peter H.; GOYEN, Jocelyn. Operant-behavioural and cognitive-behavioural treatment for chronic low back pain. *Behaviour Research and Therapy*, v. 29, n. 3, p. 225–238, 1991. Disponível em: <https://pubmed.ncbi.nlm.nih.gov/1831972/>. Acesso em: 8 jun. 2026.

NIM, Casper Glissmann *et al.* Changes in pain sensitivity and spinal stiffness in relation to responder status following spinal manipulative therapy in chronic low Back pain: a secondary explorative analysis of a randomized trial. *BMC Musculoskeletal Disorders*, v. 22, n. 1, p. 23, 6 dez. 2021.

NIM, Casper Glissmann *et al.* The effect on clinical outcomes when targeting spinal manipulation at stiffness or pain sensitivity: a randomized trial. *Scientific Reports*, v. 10, n. 1, p. 14615, 3 set. 2020.

NISHIMURA, Takaaki *et al.* Effect of Interventions for Improving Lumbar Motor Control on Low Back Pain in Sedentary Office Workers: A Randomized Controlled Trials. *Physical therapy research*, v. 24, p. 240–248, 2021.

NOUWEN, Arie. EMG biofeedback used to reduce standing levels of paraspinal muscle tension in chronic low back pain. *Pain*, v. 17, n. 4, p. 353–360, 1983.

NWUGA, Gladys; NWUGA, Vincent. Relative therapeutic efficacy of the Williams and McKenzie protocols in back pain management. *Physiotherapy practice*, v. 1, n. 2, p. 99–105, 1985.

OH, Wan Soo; SHIM, Jae Chol. A Randomized Controlled Trial of Radiofrequency Denervation of the Ramus Communicans Nerve for Chronic Discogenic Low Back Pain. *The Clinical Journal of Pain*, v. 20, n. 1, p. 55–60, jan. 2004.

OH, Youn-Jung; PARK, Sam-Ho; LEE, Myung-Mo. Comparison of Effects of Abdominal Draw-In Lumbar Stabilization Exercises with and without Respiratory Resistance on Women with Low Back Pain: A Randomized Controlled Trial. *Medical Science Monitor*, v. 26, 21 jan. 2020.

OH, Youn-Jung; PARK, Sam-Ho; MYUNG-MO. The effect of soft tissue mobilization on pain, disability level and depressive symptoms in patients with chronic low back pain. *Annals of Clinical and Analytical Medicine*, v. 11, n. 04, 2020.

O'HAGAN, Edel T. *et al.* Development of a booster intervention for graded sensorimotor retraining (RESOLVE) in people with persistent low back pain: A nested, randomised, feasibility trial. *Musculoskeletal Care*, v. 21, n. 2, p. 444–452, 26 jun. 2023.

O'KEEFFE, Mary *et al.* Cognitive functional therapy compared with a group-based exercise and education intervention for chronic low back pain: a multicentre randomised controlled trial (RCT). *British Journal of Sports Medicine*, v. 54, n. 13, p. 782–789, jul. 2020.

OKUDAN, Begum; ÇELIK, Derya; KOBAN, Orkun. The effectiveness of telerehabilitation-based exercise combined with pain neuroscience education for patients with facet joint arthrosis: A randomized controlled study. *European Journal of Pain*, v. 28, n. 3, p. 382–395, 25 mar. 2024.

OLKOSKI, Mabel M. *et al.* Comparing the effects of aquatic exercises with or without high intensity on the functional status, muscular endurance, and performance of patients with chronic low back pain. *The Journal of Sports Medicine and Physical Fitness*, v. 61, n. 5, maio 2021.

ONGLEY, MilneJ *et al.* A new approach to the treatment of chronic low back pain. *The Lancet*, v. 330, n. 8551, p. 143–146, 1987.

ONSY, Hadil Mohamed *et al.* Effect of Gluteus Medius Strengthening on Pain, Function, and Muscle Macromorphology in Nonspecific Chronic Low Back Pain: Randomized Controlled Trial. *Muscles, Ligaments and Tendons Journal*, v. 14, n. 3, 30 set. 2024.

OPEYEMI AYODIIPO IDOWU; ADE FATAI ADENIYI. Efficacy of Graded Activity with and without Daily-Monitored-Walking on Pain and Back Endurance among Patients with Concomitant Low-Back Pain and Type-2 Diabetes: A Randomized Trial. *Ethiopian Journal of Health Sciences*, v. 30, n. 2, 1 mar. 2020.

ORTIZ, Ralph *et al.* Sustained acoustic medicine treatment of discogenic chronic low back pain: A randomized, multisite, double-blind, placebo-controlled trial. *Journal of Back and Musculoskeletal Rehabilitation*, v. 37, n. 5, p. 1321–1332, 3 set. 2024.

O'SULLIVAN, Peter B.; TWOMEY, Lance T.; ALLISON, Garry T. Evaluation of specific stabilizing exercise in the treatment of chronic low back pain with radiologic diagnosis of spondylolysis or spondylolisthesis. *Spine*, v. 22, n. 24, p. 2959–2967, 15 dez. 1997. Disponível em: <https://pubmed.ncbi.nlm.nih.gov/9431633/>. Acesso em: 8 jun. 2026.

OVERMAN, Steven S *et al.* Physical therapy care for low back pain: monitored program of first-contact nonphysician care. *Physical Therapy*, v. 68, n. 2, p. 199–207, 1988.

OZ, Muzeyyen; ULGER, Ozlem. Yoga, Physical Therapy and Home Exercise Effects on Chronic Low Back Pain: Pain Perception, Function, Stress, and Quality of Life in a Randomized Trial. *Perceptual and Motor Skills*, v. 131, n. 6, p. 2216–2243, 15 dez. 2024.

ÖZDEN, Fatih *et al.* The effect of video exercise-based telerehabilitation on clinical outcomes, expectation, satisfaction, and motivation in patients with chronic low back pain. *Irish Journal of Medical Science (1971 -)*, v. 191, n. 3, p. 1229–1239, 6 jun. 2022.

ÖZDEN, Fatih *et al.* The effect of visual feedback-based clinical monitoring application in patients with chronic low back pain: a randomized controlled trial. *European Spine Journal*, v. 33, n. 2, p. 505–516, 6 fev. 2024.

OŻÓG, Piotr *et al.* Analysis of Muscle Activity Following the Application of Myofascial Release Techniques for Low-Back Pain—A Randomized-Controlled Trial. *Journal of Clinical Medicine*, v. 10, n. 18, p. 4039, 7 set. 2021.

OŻÓG, Piotr *et al.* Analysis of Postural Stability Following the Application of Myofascial Release Techniques for Low Back Pain—A Randomized-Controlled Trial. *International Journal of Environmental Research and Public Health*, v. 20, n. 3, p. 2198, 26 jan. 2023.

OZSOY, Gulsah *et al.* <p>The Effects Of Myofascial Release Technique Combined With Core Stabilization Exercise In Elderly With Non-Specific Low Back Pain: A Randomized Controlled, Single-Blind Study</p>. *Clinical Interventions in Aging*, v. Volume 14, p. 1729–1740, out. 2019.

PAGNEZ, Maria Alice Mainenti *et al.* Different exposure times of flexion distraction technique in the L5-S1 distance and local pain of patients with chronic low back pain: A feasibility study. *International Journal of Osteopathic Medicine*, v. 51, p. 100714, mar. 2024.

PALLAY, RM *et al.* Etoricoxib reduced pain and disability and improved quality of life in patients with chronic low back pain: a 3 month, randomized, controlled trial. *Scandinavian Journal of Rheumatology*, v. 33, n. 4, p. 257–266, 12 jul. 2004.

PAOLUCCI, Teresa *et al.* Improved interoceptive awareness in chronic low back pain: a comparison of Back school versus Feldenkrais method. *Disability and Rehabilitation*, v. 39, n. 10, p. 994–1001, 8 maio 2017.

PAPOLI, Amirmohammad Faal; HOSSEINI, Seyed Mohammad; MIRKARIMPOUR, Seyed Hossein. Effects of different treatments on pain, functional disability, position sense and range of motion in elite bodybuilders with chronic low back pain. *Scientific Reports*, v. 14, n. 1, p. 9176, 22 abr. 2024.

PARK, Chanhee; YI, Chunghwi; *et al.* Long-term effects of deep-learning digital therapeutics on pain, movement control, and preliminary cost-effectiveness in low back pain: A randomized controlled trial. *DIGITAL HEALTH*, v. 9, 3 jan. 2023.

PARK, Chanhee; KIM, Kyoungtae; *et al.* Physiotherapeutic effects of an innovative golf swing-assist device on discomfort and mobility in amateur golfers with low back pain: A randomized controlled trial. *Technology and Health Care*, v. 31, p. 137–144, 28 abr. 2023.

PARK, Chul-Hyun *et al.* Long-term effects of lumbar flexion versus extension exercises for chronic axial low back pain: a randomized controlled trial. *Scientific Reports*, v. 14, n. 1, p. 2714, 1 fev. 2024.

PARK, Kyoung Sun *et al.* A Pragmatic Randomized Controlled Trial on the Effectiveness and Safety of Pharmacopuncture for Chronic Lower Back Pain. *Journal of Pain Research*, v. Volume 16, p. 2697–2712, ago. 2023.

PARK, Kyue-nam *et al.* Effects of Motor Control Exercise Vs Muscle Stretching Exercise on Reducing Compensatory Lumbopelvic Motions and Low Back Pain: A Randomized Trial. *Journal of Manipulative and Physiological Therapeutics*, v. 39, n. 8, p. 576–585, out. 2016.

PARK, Sihwa *et al.* A Randomized Controlled Trial Investigating the Effects of Equine Simulator Riding on Low Back Pain, Morphological Changes, and Trunk Musculature in Elderly Women. *Medicina*, v. 56, n. 11, p. 610, 13 nov. 2020.

PARK, Sun Ja; KIM, Young Mi; YANG, Sung Rae. Effects of lumbar segmental stabilization exercise and respiratory exercise on the vital capacity in patients with chronic back pain. *Journal of Back and Musculoskeletal Rehabilitation*, v. 33, n. 5, p. 841–848, 18 set. 2020.

PASSOS, Muana Hiandra Pereira Dos *et al.* Effects of an eight-week physical exercise program on low back pain and function in fruit workers: A randomized controlled trial. *Journal of Back and Musculoskeletal Rehabilitation*, v. 37, n. 3, p. 733–742, 10 maio 2024.

PATIL, NitinJ *et al.* A randomized trial comparing effect of yoga and exercises on quality of life in among nursing population with chronic low back pain. *International Journal of Yoga*, v. 11, n. 3, p. 208, 2018.

PATTI, Antonino *et al.* Pain Perception and Stabilometric Parameters in People With Chronic Low Back Pain After a Pilates Exercise Program. *Medicine*, v. 95, n. 2, p. e2414, jan. 2016.

PAULO, Luana Rocha *et al.* Can a Single Trial of a Thoracolumbar Myofascial Release Technique Reduce Pain and Disability in Chronic Low Back Pain? A Randomized Balanced Crossover Study. *Journal of Clinical Medicine*, v. 10, n. 9, p. 2006, 7 maio 2021.

PAUNGMALI, Aatit *et al.* Lumbopelvic Core Stabilization Exercise and Pain Modulation Among Individuals with Chronic Nonspecific Low Back Pain. *Pain Practice*, v. 17, n. 8, p. 1008–1014, 25 nov. 2017.

PECOS-MARTÍN, Daniel *et al.* Optimizing dry needling for chronic low back pain: A novel approach to treatment site selection: A randomized controlled trial. *Journal of Bodywork and Movement Therapies*, v. 40, p. 2113–2119, out. 2024.

PEHLEVAN, Elif; ŞEVGIN, Ömer. Effect of exercise given to factory workers with ergonomics training on pain and functionality: A randomized controlled trial. *WORK: A Journal of Prevention, Assessment & Rehabilitation*, v. 78, n. 1, p. 195–205, 13 maio 2024.

PENG, Meng-Si *et al.* Efficacy of Therapeutic Aquatic Exercise vs Physical Therapy Modalities for Patients With Chronic Low Back Pain. *JAMA Network Open*, v. 5, n. 1, p. e2142069, 7 jan. 2022.

PETIT, Audrey *et al.* Effectiveness of three treatment strategies on occupational limitations and quality of life for patients with non-specific chronic low back pain: Is a multidisciplinary approach the key feature to success: study protocol for a randomized controlled trial. *BMC Musculoskeletal Disorders*, v. 15, n. 1, p. 131, 16 dez. 2014.

PETROFSKY, Jerrold; LAYMON, Michael; LEE, Haneul. The effect of transcutaneous electrical nerve stimulation and low-level continuous heat on non-specific low back pain: a randomized controlled trial. *Gazzetta Medica Italiana Archivio per le Scienze Mediche*, v. 179, n. 6, jul. 2020.

PETROZZI, M. John *et al.* Addition of MoodGYM to physical treatments for chronic low back pain: A randomized controlled trial. *Chiropractic & Manual Therapies*, v. 27, n. 1, p. 54, 25 dez. 2019.

PHATTHARASUPHARERK, Suttinee *et al.* Effects of Qigong practice in office workers with chronic non-specific low back pain: A randomized control trial. *Journal of Bodywork and Movement Therapies*, v. 23, n. 2, p. 375–381, abr. 2019.

PINHO, Helena *et al.* Pain intensity and pain sensitivity are not increased by a single session of high-intensity interval aerobic exercise in individuals with chronic low back pain: A randomized and controlled trial. *Musculoskeletal Science and Practice*, v. 66, p. 102824, ago. 2023.

PLANDOWSKA, Magdalena *et al.* A Randomized Controlled Trial of Active Stretching of the Hamstrings and Core Control for Low Back Pain and Musculoskeletal Discomfort during Prolonged Sitting among Young People. *Journal of Clinical Medicine*, v. 13, n. 17, p. 5048, 26 ago. 2024.

POCOVI, Natasha C *et al.* Effectiveness and cost-effectiveness of an individualised, progressive walking and education intervention for the prevention of low back pain recurrence in Australia (WalkBack): a randomised controlled trial. *The Lancet*, v. 404, n. 10448, p. 134–144, jul. 2024.

POLLY, David W. *et al.* Two-Year Outcomes from a Randomized Controlled Trial of Minimally Invasive Sacroiliac Joint Fusion vs. Non-Surgical Management for Sacroiliac Joint Dysfunction. *International Journal of Spine Surgery*, v. 10, p. 28, 1 jan. 2016.

POPOVICH, John M. *et al.* The effects of osteopathic manipulative treatment on pain and disability in patients with chronic low back pain: a single-blinded randomized controlled trial. *Journal of Osteopathic Medicine*, v. 124, n. 5, p. 219–230, 23 abr. 2024.

POURAHMADI, Mohammad R. *et al.* Effect of Elastic Therapeutic Taping on Abdominal Muscle Endurance in Patients With Chronic Nonspecific Low Back Pain: A Randomized, Controlled, Single-Blind, Crossover Trial. *Journal of Manipulative and Physiological Therapeutics*, v. 41, n. 7, p. 609–620, set. 2018.

PROSKURA, Patrycja; RUTKOWSKA-KUCHARSKA, Alicja; SOBERA, Małgorzata. Evaluation of the effects of a novel exercise program in the treatment of low back pain in women working in a seated position: A randomized trial. *Journal of Back and Musculoskeletal Rehabilitation*, v. 36, n. 4, p. 845–859, 30 jun. 2023.

QIMING, Zhang *et al.* Effect of deep muscle stimulation on muscle tone and stiffness of erector spinaes in patients with chronic nonspecific low back pain: a digital muscle testing. *Chinese Journal of Tissue Engineering Research*, v. 27, n. 8, p. 1250, 18 mar. 2023. Disponível em: <https://www.cjter.com/EN/10.12307/2022.1024>. Acesso em: 10 jun. 2026.

RABIEEZADEH, Alireza *et al.* The effects of an 8-week dynamic neuromuscular stabilization exercise on pain, functional disability, and quality of life in individuals with non-specific chronic low back pain: a randomized clinical trial with a two-month follow-up study. *BMC Sports Science, Medicine and Rehabilitation*, v. 16, n. 1, p. 161, 25 jul. 2024.

RABIEI, Pouya; SHEIKHI, Bahram; LETAFATKAR, Amir. Comparing Pain Neuroscience Education Followed by Motor Control Exercises With Group-Based Exercises for Chronic Low Back Pain: A Randomized Controlled Trial. *Pain Practice*, v. 21, n. 3, p. 333–342, 21 mar. 2021.

RAHBAR, Mohammad *et al.* Effect of hippotherapy simulator on pain, disability and range of motion of the spinal column in subjects with mechanical low back pain: A randomized single-blind clinical trial. *Journal of Back and Musculoskeletal Rehabilitation*, v. 31, n. 6, p. 1183–1192, 28 nov. 2018.

RAJAN, Abey P.; GURUDUT, Peeyoosha. Gross myofascial release of trunk with leg pull technique on low back pain with radiculopathy — A randomised controlled trial. *Hong Kong Physiotherapy Journal*, v. 43, n. 01, p. 61–71, 31 jun. 2023.

RAJFUR, Joanna *et al.* Dry Needling with the Use of FRSc Technique in Addition to Standard Rehabilitation Program for Chronic Low Back Pain: A Randomized Controlled Trial Using Both PROMs and Measurement Tools. *Journal of Pain Research*, v. Volume 17, p. 1041–1053, mar. 2024.

RAJFUR, Joanna *et al.* The effectiveness of dry needling in patients with chronic low back pain: a prospective, randomized, single-blinded study. *Scientific Reports*, v. 12, n. 1, p. 15803, 22 set. 2022.

RAJFUR, Katarzyna *et al.* Efficacy of Focused Extracorporeal Shock Wave Therapy in Chronic Low Back Pain: A Prospective Randomized 3-Month Follow-Up Study. *Medical Science Monitor*, v. 28, 20 maio 2022.

RASMUSSEN-BARR, E.; NILSSON-WIKMAR, L.; ARVIDSSON, I. Stabilizing training compared with manual treatment in sub-acute and chronic low-back pain. *Manual Therapy*, v. 8, n. 4, p. 233–241, 1 nov. 2003. Disponível em: <https://www.sciencedirect.com/science/article/abs/pii/S1356689X03000535>. Acesso em: 8 jun. 2026.

REILLY, Kevin *et al.* Differences between Supervised and Independent Strength and Conditioning Program with Chronic Low Back Syndromes. *Journal of Occupational Medicine*, p. 547–550, 1989.

REINECKE, Steven M.; HAZARD, Rowland G.; COLEMAN, Kevin. Continuous passive motion in seating: a new strategy against low back pain. *Journal of spinal disorders*, v. 7, n. 1, p. 29–35, 1994. Disponível em: <https://pubmed.ncbi.nlm.nih.gov/8186586/>. Acesso em: 8 jun. 2026.

REUST, P; CHANTRAINE, A; VISCHER, T L. Treatment of lumbar sciatica with or without neurological deficit using mechanical traction. A double-blind study. *Schweizerische Medizinische Wochenschrift*, v. 118, n. 8, p. 271–274, 1988.

RHON, Daniel I. *et al.* Effect of Risk-Stratified Care on Disability Among Adults With Low Back Pain Treated in the Military Health System. *JAMA Network Open*, v. 6, n. 7, p. e2321929, 6 jul. 2023.

RHON, Daniel I. *et al.* The influence of a MOBile-based video Instruction for Low back pain (MOBIL) on initial care decisions made by primary care providers: a randomized controlled trial. *BMC Family Practice*, v. 22, n. 1, p. 200, 9 dez. 2021.

RHON, Daniel I.; MILLER, Robert B.; FRITZ, Julie M. Effectiveness and Downstream Healthcare Utilization for Patients That Received Early Physical Therapy Versus Usual Care for Low Back Pain. *Spine*, v. 43, n. 19, p. 1313–1321, 1 out. 2018.

RIVERO-ARIAS, Oliver *et al.* Cost-utility analysis of physiotherapy treatment compared with physiotherapy advice in low back pain. *Spine*, v. 31, n. 12, p. 1381–1387, maio 2006. Disponível em: <https://pubmed.ncbi.nlm.nih.gov/16721304/>. Acesso em: 8 jun. 2026.

RIZZO, Rodrigo R. N. *et al.* The mediating effect of pain catastrophizing on pain intensity: The influence of the timing of assessments. *European Journal of Pain*, v. 25, n. 9, p. 1938–1947, 21 out. 2021.

RIZZO, Rodrigo R.N. *et al.* Hypnosis Enhances the Effects of Pain Education in Patients With Chronic Nonspecific Low Back Pain: A Randomized Controlled Trial. *The Journal of Pain*, v. 19, n. 10, p. 1103.e1-1103.e9, out. 2018.

ROLSTED HANSEN, Finn *et al.* Intensive, dynamic back-muscle exercises, conventional physiotherapy, or placebo-control treatment of low-back pain. A randomized, observer-blind trial. *Spine*, v. 18, n. 1, p. 98–108, 1993. Disponível em: <https://pubmed.ncbi.nlm.nih.gov/8434332/>. Acesso em: 8 jun. 2026.

ROLVING, Nanna *et al.* Does group-based cognitive therapy improve functional ability, pain, catastrophic thoughts and quality of life in patients with persistent low back pain and psychological risk factors? A randomised controlled trial in a secondary care setting. *Clinical Rehabilitation*, v. 36, n. 3, p. 317–330, 30 mar. 2022.

ROSE, Michael J. *et al.* Chronic low back pain rehabilitation programs: a study of the optimum duration of treatment and a comparison of group and individual therapy. *Spine*, v. 22, n. 19, p. 2246–2253, 1 out. 1997. Disponível em: <https://pubmed.ncbi.nlm.nih.gov/9346145/>. Acesso em: 8 jun. 2026.

ROSEEN, Eric J. *et al.* Yoga Versus Education for Veterans with Chronic Low Back Pain: a Randomized Controlled Trial. *Journal of General Internal Medicine*, v. 38, n. 9, p. 2113–2122, 17 jul. 2023.

ROSENSTEIN, Brent *et al.* Aquatic exercise versus standard care on paraspinal muscle morphology and function in chronic low back pain patients: a randomized controlled trial. *Scientific Reports*, v. 15, n. 1, p. 15798, 6 maio 2025.

RYYNÄNEN, Katja *et al.* Effectiveness of training in guideline-oriented biopsychosocial management of low-back pain in occupational health services – a cluster randomized controlled trial. *Scandinavian Journal of Work, Environment & Health*, v. 47, n. 5, p. 367–376, 1 jul. 2021.

SAARIJÄRVI, S. *et al.* Couple therapy improves mental well-being in chronic low back pain patients. A controlled, five year follow-up study. *Journal of Psychosomatic Research*, v. 36, n. 7, p. 651–656, 1 out. 1992. Disponível em: <https://www.sciencedirect.com/science/article/abs/pii/0022399992900546?via%3Dihub>. Acesso em: 8 jun. 2026.

SAARIJÄRVI, Simo. A controlled study of couple therapy in chronic low back pain patients. Effects on marital satisfaction, psychological distress and health attitudes. *Journal of Psychosomatic Research*, v. 35, n. 2–3, p. 265–272, 1991. Disponível em: <https://pubmed.ncbi.nlm.nih.gov/1828508/>. Acesso em: 8 jun. 2026.

SADLER, Sean *et al.* A randomised controlled trial investigating the effect of foot orthoses for the treatment of chronic nonspecific low back pain. *Musculoskeletal Care*, v. 21, n. 3, p. 856–864, 23 set. 2023.

SAFYELDEEN, Nourhan Ahmed *et al.* Effect of progressive muscle relaxation exercises versus pilates exercises on postnatal low back pain. *Advances in Rehabilitation*, v. 38, n. 3, p. 16–24, 1 set. 2024.

ŞAHIN, Nilay. Effectiveness of physical therapy and exercise on pain and functional status in patients with chronic low back pain: a randomized-controlled trial. *Turkish Journal of Physical Medicine and Rehabilitation*, v. 64, n. 1, p. 52–58, 21 mar. 2017.

SAIKLANG, Pongsatorn *et al.* An Evaluation of an Innovative Exercise to Relieve Chronic Low Back Pain in Sedentary Workers. *Human Factors: The Journal of the Human Factors and Ergonomics Society*, v. 64, n. 5, p. 820–834, 28 ago. 2022.

SAJADI, Nashmin *et al.* Effects of Different Frequencies of Whole Body Vibration on Repositioning Error in Patients With Chronic Low Back Pain in Different Angles of Lumbar Flexion. *Journal of Manipulative and Physiological Therapeutics*, v. 42, n. 4, p. 227–236, maio 2019.

SALEMI, Marianna de Melo *et al.* Effect of Dry Cupping Therapy on Pain and Functional Disability in Persistent Non-Specific Low Back Pain: A Randomized Controlled Clinical Trial. *Journal of Acupuncture and Meridian Studies*, v. 14, n. 6, p. 219–230, 31 dez. 2021.

SAMANI, Mahbobeh *et al.* A randomized controlled trial comparing the long-term use of soft lumbosacral orthoses at two different pressures in patients with chronic nonspecific low back pain. *Clinical Biomechanics*, v. 69, p. 87–95, out. 2019.

SANDERS, Ariëtte R. J. *et al.* The effectiveness of shared decision-making followed by positive reinforcement on physical disability in the long-term follow-up of patients with nonspecific low back pain in primary care: a clustered randomised controlled trial. *BMC Family Practice*, v. 19, n. 1, p. 102, 28 dez. 2018.

SANEI, Mohamad *et al.* Persian manual therapy method for chronic low-back pain with lumbar radiculopathy; a randomized controlled trial. *Journal of Bodywork and Movement Therapies*, v. 24, n. 3, p. 123–130, jul. 2020.

SANER, Jeannette *et al.* A tailored exercise program versus general exercise for a subgroup of patients with low back pain and movement control impairment: A randomised controlled trial with one-year follow-up. *Manual Therapy*, v. 20, n. 5, p. 672–679, out. 2015.

SANIKA, Vaidya; PREM, Venkatesan; KARVANNAN, Harikesan. Comparison of Glutues Maximus Activation to Flexion Bias Exercises Along with MET Technique in Subjects with Anterior Rotated Sacroiliac Joint Dysfunction -a Randomised Controlled Trial. *International journal of therapeutic massage & bodywork*, v. 14, n. 1, p. 30–38, mar. 2021.

SAPER, Robert B *et al.* Yoga vs. physical therapy vs. education for chronic low back pain in predominantly minority populations: study protocol for a randomized controlled trial. *Trials*, v. 15, n. 1, p. 67, 26 dez. 2014.

SARAÇOĞLU, İsmail *et al.* Preoperative pain neurophysiology education for lumbar radiculopathy: A randomized-controlled trial. *Turkish Journal of Physical Medicine and Rehabilitation*, v. 67, n. 3, p. 328–335, 1 set. 2021.

SARACOGLU, Ismail *et al.* The effectiveness of pain neuroscience education combined with manual therapy and home exercise for chronic low back pain: A single-blind randomized controlled trial. *Physiotherapy Theory and Practice*, v. 38, n. 7, p. 868–878, 3 jul. 2022.

SARACOGLU, Ismail *et al.* The short-term effects of neuroscience pain education on quality of life in patients with chronic low back pain: A single-blinded randomized controlled trial. *European Journal of Integrative Medicine*, v. 33, p. 101046, jan. 2020.

SATO, Takashi *et al.* Effects of Nintendo Ring Fit Adventure Exergame on Pain and Psychological Factors in Patients with Chronic Low Back Pain. *Games for Health Journal*, v. 10, n. 3, p. 158–164, 1 jun. 2021.

SATOR-KATZENSCHLAGER, Sabine M. *et al.* The short- and long-term benefit in chronic low back pain through adjuvant electrical versus manual auricular acupuncture. *Anesthesia and analgesia*, v. 98, n. 5, p. 1359–1364, 2004. Disponível em: <https://pubmed.ncbi.nlm.nih.gov/15105215/>. Acesso em: 8 jun. 2026.

SAWANT, Rakhi Sadanand; SHINDE, Sandeep Babasaheb. Effect of hydrotherapy based exercises for chronic nonspecific low back pain. *Indian Journal of Physiotherapy and Occupational Therapy - An International Journal*, v. 13, n. 1, p. 133, 2019.

SCHALLER, Andrea *et al.* Promoting physical activity in low back pain patients: six months follow-up of a randomised controlled trial comparing a multicomponent intervention with a low intensity intervention. *Clinical Rehabilitation*, v. 30, n. 9, p. 865–877, 4 set. 2016.

SCHENK, Ronald J.; JOZEFCZYK, Cherie; KOPF, Aric. A Randomized Trial Comparing Interventions in Patients with Lumbar Posterior Derangement. *Journal of Manual & Manipulative Therapy*, v. 11, n. 2, p. 95–102, 2003. Disponível em: <https://www.tandfonline.com/doi/abs/10.1179/106698103790826455>. Acesso em: 8 jun. 2026.

SCHMIDT, Anne Mette *et al.* The effect of an integrated multidisciplinary rehabilitation programme alternating inpatient interventions with home-based activities for patients with chronic low back pain: a randomized controlled trial. *Clinical Rehabilitation*, v. 34, n. 3, p. 382–393, 8 mar. 2020.

SCHNEIDER, Michael *et al.* Comparison of Spinal Manipulation Methods and Usual Medical Care for Acute and Subacute Low Back Pain. *Spine*, v. 40, n. 4, p. 209–217, fev. 2015.

SCHRÖDER, Karin *et al.* Effectiveness and Quality of Implementing a Best Practice Model of Care for Low Back Pain (BetterBack) Compared with Routine Care in Physiotherapy: A Hybrid Type 2 Trial. *Journal of Clinical Medicine*, v. 10, n. 6, p. 1230, 16 mar. 2021.

SCHRÖDER, Karin *et al.* Improved adherence to clinical guidelines for low back pain after implementation of the BetterBack model of care: A stepped cluster randomized controlled trial within a hybrid type 2 trial. *Physiotherapy Theory and Practice*, v. 39, n. 7, p. 1376–1390, 3 jul. 2023.

SCHWEIKERT, Bernd *et al.* Effectiveness and cost-effectiveness of adding a cognitive behavioral treatment to the rehabilitation of chronic low back pain. *The Journal of Rheumatology*, v. 33, n. 12, 2006. Acesso em: 8 jun. 2026.

SCULCO, Arthur Daniel *et al.* Effects of aerobic exercise on low back pain patients in treatment. *Spine Journal*, v. 1, n. 2, p. 95–101, mar. 2001. Disponível em: <https://pubmed.ncbi.nlm.nih.gov/14588388/>. Acesso em: 8 jun. 2026.

SEMRAU, Jana *et al.* Effects of behavioural exercise therapy on the effectiveness of multidisciplinary rehabilitation for chronic non-specific low back pain: a randomised controlled trial. *BMC Musculoskeletal Disorders*, v. 22, n. 1, p. 500, 29 dez. 2021.

SENBURSA, Gamze; PEKYAVAS, Nihan Ozunlu; BALTACI, Gul. Comparison of Physiotherapy Approaches in Low Back Pain: A Randomized Controlled Trial. *Korean Journal of Family Medicine*, v. 42, n. 2, p. 96–106, 20 mar. 2021.

SEO, Hye-Ran; KIM, Tae-Ho. The effects of Gyrotonic expansion system exercise and trunk stability exercise on muscle activity and lumbar stability for the subjects with chronic low back pain. *Journal of Exercise Rehabilitation*, v. 15, n. 1, p. 129–133, 25 fev. 2019.

SEO, U-Hyeok; KIM, Jung-Hee; LEE, Byoung-Hee. Effects of Mulligan Mobilization and Low-Level Laser Therapy on Physical Disability, Pain, and Range of Motion in Patients with Chronic Low Back Pain: A Pilot Randomized Controlled Trial. *Healthcare*, v. 8, n. 3, p. 237, 29 jul. 2020.

SHABAT, S. *et al.* The effect of insoles on the incidence and severity of low back pain among workers whose job involves long-distance walking. *European spine journal : official publication of the European Spine Society, the European Spinal Deformity Society, and the European Section of the Cervical Spine Research Society*, v. 14, n. 6, p. 546–550, ago. 2005. Disponível em: <https://pubmed.ncbi.nlm.nih.gov/15668775/>. Acesso em: 8 jun. 2026.

SHAH, Suken A.; SALLER, Jeremy. Evaluation and Diagnosis of Back Pain in Children and Adolescents. *Journal of the American Academy of Orthopaedic Surgeons*, v. 24, n. 1, p. 37–45, jan. 2016.

SHAHEEN, Hamza M.; BELGEN KAYGISIZ, Beliz. Comparison of different treatment positions of nerve slider technique for patients with low back pain: a randomized control trial. *European Journal of Physical and Rehabilitation Medicine*, v. 61, n. 1, fev. 2025.

SHAMSI, Mohammad Bagher; SARRAFZADEH, Javad; JAMSHIDI, Aliashraf. Comparing core stability and traditional trunk exercise on chronic low back pain patients using three functional lumbopelvic stability tests. *Physiotherapy Theory and Practice*, v. 31, n. 2, p. 89–98, 17 fev. 2015.

SHAMSI, MohammadBagher *et al.* Comparing the effects of static stretching and strengthening in lengthened position on EMG activity of hamstring muscle in patients with chronic non-specific LBP having shortened muscle: a randomised controlled clinical trial. *European Journal of Physiotherapy*, v. 22, n. 4, p. 184–190, 3 jul. 2020.

SHAMSI, MohammadBagher *et al.* The effect of core stability and general exercise on abdominal muscle thickness in non-specific chronic low back pain using ultrasound imaging. *Physiotherapy Theory and Practice*, v. 32, n. 4, p. 277–283, 18 maio 2016.

SHARIAT, Ardalan *et al.* The impact of modified exercise and relaxation therapy on chronic lower back pain in office workers: a randomized clinical trial. *Journal of Exercise Rehabilitation*, v. 15, n. 5, p. 703–708, 28 out. 2019.

SHARMA, Saurab *et al.* Results of a feasibility randomised clinical trial on pain education for low back pain in Nepal: the Pain Education in Nepal-Low Back Pain (PEN-LBP) feasibility trial. *BMJ Open*, v. 9, n. 3, p. e026874, 27 mar. 2019.

SHEBIB, Raad *et al.* Randomized controlled trial of a 12-week digital care program in improving low back pain. *npj Digital Medicine*, v. 2, n. 1, p. 1, 7 jan. 2019.

SHEKELLE, Paul G. *et al.* Spinal manipulation for low-back pain. *Annals of internal medicine*, v. 117, n. 7, p. 590–598, 1 out. 1992. Disponível em: <https://pubmed.ncbi.nlm.nih.gov/1388006/>. Acesso em: 8 jun. 2026.

SHERRY, E.; KITCHENER, P.; SMART, R. A prospective randomized controlled study of VAX-D and TENS for the treatment of chronic low back pain. *Neurological research*, v. 23, n. 7, p. 780–784, 2001. Disponível em: <https://pubmed.ncbi.nlm.nih.gov/11680522/>. Acesso em: 8 jun. 2026.

SHI, Weihong *et al.* The Physical and Psychological Effects of Telerehabilitation-Based Exercise for Patients With Nonspecific Low Back Pain: Prospective Randomized Controlled Trial. *JMIR mHealth and uHealth*, v. 12, p. e56580–e56580, 6 set. 2024.

SHIN, Sun-shil; LEE, Mi-ra; YOO, Won-gyu. The Intertester Reliability of a Modified Movement System Impairment Classification System Used to Evaluate Individuals With Prolonged Sitting: A Preliminary Study. *Journal of Manipulative and Physiological Therapeutics*, v. 43, n. 4, p. 294–302, maio 2020.

SIDIQ, Mohammad *et al.* Effects of pain education on disability, pain, quality of life, and self-efficacy in chronic low back pain: A randomized controlled trial. *PLOS ONE*, v. 19, n. 5, p. e0294302, 28 maio 2024.

SIĞLAN, Ümit; ÇOLAK, Serpil. Effects of diaphragmatic and iliopsoas myofascial release in patients with chronic low back pain: A randomized controlled study. *Journal of Bodywork and Movement Therapies*, v. 33, p. 120–127, jan. 2023.

ŞIMŞEK, Şule; YAĞCI, Nesrin; KORKMAZ, Merve Bergin. Mid-term Effect of Lumbar Sustained Natural Apophyseal Glides in Patients with Non-specific Chronic Low Back Pain: A Randomized Clinical Trial. *The Eurasian Journal of Medicine*, v. 55, n. 2, p. 152–157, 1 jun. 2023.

SIMULA, Anna S. *et al.* Patient education booklet to support evidence-based low back pain care in primary care – a cluster randomized controlled trial. *BMC Family Practice*, v. 22, n. 1, p. 178, 7 dez. 2021.

SINGPHOW, Chametcha *et al.* Effect of Yoga on Stress, Anxiety, Depression, and Spinal Mobility in Computer Users with Chronic Low Back Pain. *International Journal of Yoga*, v. 15, n. 2, p. 114–121, maio 2022.

SIPAVICIENE, Saule; KLIZIENE, Irina. Effect of different exercise programs on non-specific chronic low back pain and disability in people who perform sedentary work. *Clinical Biomechanics*, v. 73, p. 17–27, mar. 2020.

SIPKO, Tomasz; GLIBOWSKI, Edmund; KUCZYŃSKI, Michał. Acute effects of proprioceptive neuromuscular facilitation exercises on the postural strategy in patients with chronic low back pain. *Complementary Therapies in Clinical Practice*, v. 44, p. 101439, ago. 2021.

SITGES, Carolina *et al.* Acute Effects of a Brief Physical Exercise Intervention on Somatosensory Perception, Lumbar Strength, and Flexibility in Patients with Nonspecific Chronic Low-Back Pain. *Journal of Pain Research*, v. Volume 14, p. 487–500, fev. 2021.

SJOGREN, T. *et al.* Group hydrotherapy versus group land-based treatment for chronic low back pain. *Physiotherapy research international : the journal for researchers and clinicians in physical therapy*, v. 2, n. 4, p. 212–222, 1997. Disponível em: <https://pubmed.ncbi.nlm.nih.gov/9408932/>. Acesso em: 8 jun. 2026.

SJÖGREN, Tuulikki *et al.* Effects of a workplace physical exercise intervention on the intensity of low back symptoms in office workers: A cluster randomized controlled cross-over design. *Journal of Back and Musculoskeletal Rehabilitation*, v. 19, n. 1, p. 13–24, 2006. Disponível em: </doi/pdf/10.3233/BMR-2006-19103?download=true>. Acesso em: 8 jun. 2026.

SKOUEN, Jan S. *et al.* Relative cost-effectiveness of extensive and light multidisciplinary treatment programs versus treatment as usual for patients with chronic low back pain on long-term sick leave: randomized controlled study. *Spine*, v. 27, n. 9, p. 901–909, 1 maio 2002. Disponível em: <https://pubmed.ncbi.nlm.nih.gov/11979157/>. Acesso em: 8 jun. 2026.

SMEETS, Rob J.E.M.; VLAEYEN, Johan W.S.; HIDDING, Alita; *et al.* Active rehabilitation for chronic low back pain: cognitive-behavioral, physical, or both? First direct post-treatment results from a randomized controlled trial [ISRCTN22714229]. *BMC musculoskeletal disorders*, v. 7, 20 jan. 2006. Disponível em: <https://pubmed.ncbi.nlm.nih.gov/16426449/>. Acesso em: 8 jun. 2026.

SMEETS, Rob J.E.M.; VLAEYEN, Johan W.S.; KESTER, Arnold D.M.; *et al.* Reduction of Pain Catastrophizing Mediates the Outcome of Both Physical and Cognitive-Behavioral Treatment in Chronic Low Back Pain. *Journal of Pain*, v. 7, n. 4, p. 261–271, abr. 2006. Disponível em: <https://pubmed.ncbi.nlm.nih.gov/16618470/>. Acesso em: 8 jun. 2026.

SNOOK, Stover H. *et al.* The reduction of chronic nonspecific low back pain through the control of early morning lumbar flexion. A randomized controlled trial. *Spine*, v. 23, n. 23, p. 2601–2607, 1 dez. 1998. Disponível em: <https://pubmed.ncbi.nlm.nih.gov/9854759/>. Acesso em: 8 jun. 2026.

SOARES, P. *et al.* Efeitos do Programa Escola de Postura e Reeducação Postural Global sobre a amplitude de movimento e níveis de dor em pacientes com lombalgia crônica. *Revista Andaluza de Medicina del Deporte*, v. 9, n. 1, p. 23–28, mar. 2016.

SON, Aria *et al.* Efficacy of Self-Natural Posture Exercise (SNPE) programs on chronic low back pain: A randomized controlled feasibility trial with waitlist control. *Journal of Back and Musculoskeletal Rehabilitation*, v. 37, n. 6, p. 1601–1616, 8 nov. 2024.

SONG, Jeongkeun *et al.* Soft-Tissue Mobilization and Pain Neuroscience Education for Chronic Nonspecific Low Back Pain with Central Sensitization: A Prospective Randomized Single-Blind Controlled Trial. *Biomedicines*, v. 11, n. 5, p. 1249, 23 abr. 2023.

SONI, Jaykumar; TOLE, Mansi. Efficacy of Back School Program and Swiss Ball Exercise on Pain and Core Endurance in Individuals with Non-Specific Low Back Pain. *Current Progress in Medicine and Medical Research Vol. 2*. [S.l.]: B P International (a part of SCIENCEDOMAIN International), 2023. p. 14–23.

SORMUNEN, Erja *et al.* Participatory Ergonomics Intervention to Prevent Work Disability Among Workers with Low Back Pain: A Randomized Clinical Trial in Workplace Setting. *Journal of Occupational Rehabilitation*, v. 32, n. 4, p. 731–742, 6 dez. 2022.

SOTIROPOULOS, Spyridon; PLAVOUKOU, Theodora; GEORGOUDIS, George. Qigong Versus Usual Exercise in the Treatment of Chronic Nonspecific Low Back Pain as an Add-On to a Standardized Physiotherapy Program. *Cureus*, 31 mar. 2025.

SOUKUP, Margreth Grotle *et al.* The effect of a Mensendieck exercise program as secondary prophylaxis for recurrent low back pain. A randomized, controlled trial with 12-month follow-up. *Spine*, v. 24, n. 15, p. 1585–1592, 1 ago. 1999. Disponível em: <https://pubmed.ncbi.nlm.nih.gov/10457579/>. Acesso em: 8 jun. 2026.

SOUNDARARAJAN, Leo Rathinaraj Antony; THANKAPPAN, Sreeja Mannickal. Efficacy of the Multifidus Retraining Program in Computer Professionals with Chronic Low Back Pain. *Asian Spine Journal*, v. 10, n. 3, p. 450, 2016.

SOYLU, Caglar *et al.* Impact of the Abdominal Drawing-In Maneuver on Spinal Extensor Muscle Activity: A Randomized Controlled Double-Blind Trial Involving Individuals with Non-Specific Low Back Pain. *International Journal of Environmental Research and Public Health*, v. 21, n. 12, p. 1675, 16 dez. 2024.

SPINHOVEN, Philip *et al.* Catastrophizing and internal pain control as mediators of outcome in the multidisciplinary treatment of chronic low back pain. *European journal of pain (London, England)*, v. 8, n. 3, p. 211–219, 2004. Disponível em: <https://pubmed.ncbi.nlm.nih.gov/15109971/>. Acesso em: 8 jun. 2026.

SPRATT, Kevin F. *et al.* Efficacy of flexion and extension treatments incorporating braces for low-back pain patients with retrodisplacement, spondylolisthesis, or normal sagittal translation. *Spine*, v. 18, n. 13, p. 1839–1849, 1993. Disponível em: <https://pubmed.ncbi.nlm.nih.gov/8235870/>. Acesso em: 8 jun. 2026.

SRIVASTAVA, Saumya *et al.* Short-term effect of muscle energy technique and mechanical diagnosis and therapy in sacroiliac joint dysfunction: A pilot randomized clinical trial. *Journal of Bodywork and Movement Therapies*, v. 24, n. 3, p. 63–70, jul. 2020.

STEELE, James *et al.* A Randomized Controlled Trial of the Effects of Isolated Lumbar Extension Exercise on Lumbar Kinematic Pattern Variability During Gait in Chronic Low Back Pain. *PM&R*, v. 8, n. 2, p. 105–114, 21 fev. 2016.

STIER, R. *et al.* [Reconditioning in groups: an effective programme for the rehabilitation of patients with low back pain?]. *Die Rehabilitation*, v. 40, n. 6, p. 321–331, 2001. Disponível em: <https://pubmed.ncbi.nlm.nih.gov/11742422/>. Acesso em: 8 jun. 2026.

STRONG, Jenny. Incorporating cognitive-behavioral therapy with occupational therapy: A comparative study with patients with low back pain. *Journal of Occupational Rehabilitation*, v. 8, n. 1, p. 61–71, 1998. Disponível em: <https://link.springer.com/article/10.1023/A:1023008632131>. Acesso em: 8 jun. 2026.

STRONG, Larkin L. *et al.* Cost-effectiveness of two self-care interventions to reduce disability associated with back pain. *Spine*, v. 31, n. 15, p. 1639–1645, jul. 2006. Disponível em: <https://pubmed.ncbi.nlm.nih.gov/16816756/>. Acesso em: 8 jun. 2026.

STUCKEY, Sandra J; JACOBS, Alfred; GOLDFARB, Jack. EMG biofeedback training, relaxation training, and placebo for the relief of chronic back pain. *Perceptual and motor skills*, v. 63, n. 3, p. 1023–1036, 1986.

STURIONA, Leandro A. *et al.* Comparison between high-velocity low-amplitude manipulation and muscle energy technique on pain and trunk neuromuscular postural control in male workers with chronic low back pain: A randomised crossover trial. *South African Journal of Physiotherapy*, v. 76, n. 1, 26 out. 2020.

SUH, Jee Hyun *et al.* The effect of lumbar stabilization and walking exercises on chronic low back pain. *Medicine*, v. 98, n. 26, p. e16173, jun. 2019.

SUMAN, Arnela *et al.* Effectiveness and cost-utility of a multifaceted eHealth strategy to improve back pain beliefs of patients with non-specific low back pain: a cluster randomised trial. *BMJ Open*, v. 9, n. 12, p. e030879, 5 dez. 2019.

SUN, Ming-Mei *et al.* [Head acupuncture combined with exercise therapy for nonspecific low back pain : a randomized controlled trial]. *Zhongguo zhen jiu = Chinese acupuncture & moxibustion*, v. 42, p. 511–4, maio 2022.

SUN, Xu *et al.* [Intervention with thumbtack needling on spinal low back pain in Air Force crew: a randomized controlled trial]. *Zhongguo zhen jiu = Chinese acupuncture & moxibustion*, v. 40, p. 1180–4, nov. 2020.

SUNI, Jaana Helena *et al.* Effectiveness and cost-effectiveness of neuromuscular exercise and back care counseling in female healthcare workers with recurrent non-specific low back pain: a blinded four-arm randomized controlled trial. *BMC Public Health*, v. 18, n. 1, p. 1376, 17 dez. 2018.

SUTTON, Laura *et al.* Navigating the maze of osteoarthritis treatment: A qualitative study exploring the experience of individuals with osteoarthritis in Tasmania, Australia. *Musculoskeletal Care*, v. 21, n. 1, p. 264–271, 12 mar. 2023.

TAGLIAFERRI, Scott D. *et al.* Randomized Trial of General Strength and Conditioning versus Motor Control and Manual Therapy for Chronic Low Back Pain on Physical and Self-Report Outcomes. *Journal of Clinical Medicine*, v. 9, n. 6, p. 1726, 3 jun. 2020.

TAHERI, Parisa; KHOSRAWI, Saeed; RAMEZANI, Mitra. Extracorporeal Shock Wave Therapy Combined With Oral Medication and Exercise for Chronic Low Back Pain: A Randomized Controlled Trial. *Archives of Physical Medicine and Rehabilitation*, v. 102, n. 7, p. 1294–1299, jul. 2021.

TAKINACI, Z Didem *et al.* Clinical efficacy of spa therapy (balneotherapy) for chronic low back pain: A randomized single-blind trial. *European Journal of Integrative Medicine*, v. 29, p. 100928, ago. 2019.

TALO, Seija; RYTOKOSKI, Ulla; PUUKKA, Pauli. Patient classification, a key to evaluate pain treatment: a psychological study in chronic low back pain patients. *Spine*, v. 17, n. 9, p. 998–1011, 1992. Disponível em: <https://pubmed.ncbi.nlm.nih.gov/1384150/>. Acesso em: 8 jun. 2026.

TAMARTASH, Hassan; BAHRPEYMA, Farid; MOKHTARI DIZAJI, Manijhe. Comparative effect of lumbar myofascial release with electrotherapy on the elastic modulus of lumbar fascia and pain in patients with non-specific low back pain. *Journal of Bodywork and Movement Therapies*, v. 29, p. 174–179, jan. 2022.

TAMBEKAR, Neha *et al*. Effect of Butler's neural tissue mobilization and Mulligan's bent leg raise on pain and straight leg raise in patients of low back ache. *Journal of Bodywork and Movement Therapies*, v. 20, n. 2, p. 280–285, abr. 2016.

TANKHA, Hallie *et al*. Effectiveness of Virtual Yoga for Chronic Low Back Pain. *JAMA Network Open*, v. 7, n. 11, p. e2442339, 1 nov. 2024.

TANTAWY, Sayed A. *et al*. Laser photobiomodulation is more effective than ultrasound therapy in patients with chronic nonspecific low back pain: a comparative study. *Lasers in Medical Science*, v. 34, n. 4, p. 793–800, 17 jun. 2019.

TAULANIEMI, Annika *et al*. Neuromuscular exercise reduces low back pain intensity and improves physical functioning in nursing duties among female healthcare workers; secondary analysis of a randomised controlled trial. *BMC Musculoskeletal Disorders*, v. 20, n. 1, p. 328, 13 dez. 2019.

TAVARES, Fernando Augusto Gonçalves *et al*. Immediate effects of joint mobilization compared to sham and control intervention for pain intensity and disability in chronic low back pain patients: randomized controlled clinical trial. *Revista Dor*, v. 18, n. 1, 2017.

TAWREJ, Poonam; KAUR, Ravinder; GHODEY, Snehal. Immediate Effect of Muscle Energy Technique on Quadratus Lumborum Muscle in Patients with Non-Specific Low Back Pain. *Indian Journal of Physiotherapy and Occupational Therapy - An International Journal*, 27 jan. 2020.

TEGNER, Heidi *et al*. The Effect of Graded Activity and Pain Education After Lumbar Spinal Fusion on Sedentary Behavior 3 and 12 Months Postsurgery: A Randomized Controlled Trial. *Archives of Physical Medicine and Rehabilitation*, v. 105, n. 8, p. 1480–1489, ago. 2024.

TEUT, Michael *et al*. Qigong or Yoga Versus No Intervention in Older Adults With Chronic Low Back Pain—A Randomized Controlled Trial. *The Journal of Pain*, v. 17, n. 7, p. 796–805, jul. 2016.

TEYCHENNE, Megan *et al*. General strength and conditioning versus motor control with manual therapy for improving depressive symptoms in chronic low back pain: A randomised feasibility trial. *PLOS ONE*, v. 14, n. 8, p. e0220442, 1 ago. 2019.

THACKERAY, Anne *et al*. The Effectiveness of Mechanical Traction Among Subgroups of Patients With Low Back Pain and Leg Pain: A Randomized Trial. *Journal of Orthopaedic & Sports Physical Therapy*, v. 46, n. 3, p. 144–154, mar. 2016.

The Effect of Core Stability Exercises Combined With Abdominal Hollowing on Postural Balance in Patients With Non-specific Chronic Low Back Pain: A Randomized Controlled Trial. *Physical Treatments - Specific Physical Therapy Journal*, v. 13, n. 3, p. 165–174, 1 jul. 2023.

THOMAS, James S. *et al*. Feasibility and Safety of a Virtual Reality Dodgeball Intervention for Chronic Low Back Pain: A Randomized Clinical Trial. *The Journal of Pain*, v. 17, n. 12, p. 1302–1317, dez. 2016.

THOMAS, M.; LUNDBERG, T. Importance of modes of acupuncture in the treatment of chronic nociceptive low back pain. *Acta anaesthesiologica Scandinavica*, v. 38, n. 1, p. 63–69, 1994. Disponível em: <https://pubmed.ncbi.nlm.nih.gov/8140876/>. Acesso em: 8 jun. 2026.

TIMM, K. E. A Randomized-Control Study of Active and Passive Treatments for Chronic Low Back Pain Following L5 Laminectomy. *https://doi.org/10.2519/jospt.1994.20.6.276*, v. 20, n. 6, p. 276–286, 1 dez. 1994. Disponível em: </doi/pdf/10.2519/jospt.1994.20.6.276>. Acesso em: 8 jun. 2026.

TOELLE, Thomas R. *et al*. App-based multidisciplinary back pain treatment versus combined physiotherapy plus online education: a randomized controlled trial. *npj Digital Medicine*, v. 2, n. 1, p. 34, 3 maio 2019.

TOMÁS-RODRÍGUEZ, Mª. Isabel *et al.* Short- and medium-term effects of a single session of pain neuroscience education on pain and psychological factors in patients with chronic low back pain. A single-blind randomized clinical trial. *European Journal of Pain*, v. 28, n. 10, p. 1841–1854, 17 nov. 2024.

TOMSCHI, Fabian; ZSCHUNKE, Andre; HILBERG, Thomas. Ten Minutes of Core Stabilisation Exercise Result in Local Exercise-Induced Hypoalgesia in Patients With Chronic Unspecific Low Back Pain. *European Journal of Pain*, v. 29, n. 3, 8 mar. 2025.

TORLAK, Mustafa S *et al.* The effect of intermittent diet and/or physical therapy in patients with chronic low back pain: A single-blinded randomized controlled trial. *EXPLORE*, v. 18, n. 1, p. 76–81, jan. 2022.

TORSTENSEN, Tom Arild *et al.* Efficiency and costs of medical exercise therapy, conventional physiotherapy, and self-exercise in patients with chronic low back pain. A pragmatic, randomized, single-blinded, controlled trial with 1-year follow-up. *Spine*, v. 23, n. 23, p. 2616–2624, 1 dez. 1998. Disponível em: <https://pubmed.ncbi.nlm.nih.gov/9854761/>. Acesso em: 8 jun. 2026.

TRIANO, John J. *et al.* Manipulative therapy versus education programs in chronic low back pain. *Spine*, v. 20, n. 8, p. 948–955, 1995. Disponível em: <https://pubmed.ncbi.nlm.nih.gov/7644961/>. Acesso em: 8 jun. 2026.

TSUI, Margaret L.K.; CHEING, Gladys L.Y. The effectiveness of electroacupuncture versus electrical heat acupuncture in the management of chronic low-back pain. *Journal of alternative and complementary medicine (New York, N.Y.)*, v. 10, n. 5, p. 803–809, out. 2004. Disponível em: <https://pubmed.ncbi.nlm.nih.gov/15650469/>. Acesso em: 8 jun. 2026.

TSUKAYAMA, Hiroshi *et al.* Randomised controlled trial comparing the effectiveness of electroacupuncture and TENS for low back pain: a preliminary study for a pragmatic trial. *Acupuncture in medicine : journal of the British Medical Acupuncture Society*, v. 20, n. 4, p. 175–180, 2002. Disponível em: <https://pubmed.ncbi.nlm.nih.gov/12512791/>. Acesso em: 8 jun. 2026.

TURNER, Judith A. Comparison of group progressive-relaxation training and cognitive-behavioral group therapy for chronic low back pain. *Journal of consulting and clinical psychology*, v. 50, n. 5, p. 757, 1982.

TURNER, Judith A; CLANCY, Stephen. Strategies for coping with chronic low back pain: Relationship to pain and disability. *Pain*, v. 24, n. 3, p. 355–364, 1986.

TURNER, Judith A; CLANCY, Steve. Comparison of operant behavioral and cognitive-behavioral group treatment for chronic low back pain. *Journal of consulting and clinical psychology*, v. 56, n. 2, p. 261, 1988.

TURNER, Judith A.; JENSEN, Mark P. Efficacy of cognitive therapy for chronic low back pain. *Pain*, v. 52, n. 2, p. 169–177, 1993. Disponível em: <https://pubmed.ncbi.nlm.nih.gov/8455964/>. Acesso em: 8 jun. 2026.

TÜZÜN, Emİne Handan *et al.* Effectiveness of dry needling versus a classical physiotherapy program in patients with chronic low-back pain: a single-blind, randomized, controlled trial. *Journal of Physical Therapy Science*, v. 29, n. 9, p. 1502–1509, 2017.

UCGUN, Hikmet; KAYA, Meltem; KULLI, Hilal Denizoglu. The effect of pelvic proprioceptive neuromuscular facilitation techniques in patients with sacroiliac joint dysfunction: A randomized controlled study. *Journal of Back and Musculoskeletal Rehabilitation*, v. 38, n. 2, p. 229–240, 11 mar. 2025.

ULUSOY, Meltem; IYIGUN, Gozde. Comparison of Proprioceptive Neuromuscular Facilitation and Pilates exercises in patients with chronic low back pain: A randomized study. *Journal of Bodywork and Movement Therapies*, v. 42, p. 463–470, jun. 2025.

USHINOHAMA, Andrea *et al.* Effect of a single session of ear acupuncture on pain intensity and postural control in individuals with chronic low back pain: a randomized controlled trial. *Brazilian Journal of Physical Therapy*, v. 20, n. 4, p. 328–335, ago. 2016.

UZLIFATIN, Yaniar *et al.* Effect of Transcutaneous Auricular Vagus Nerve Stimulation Addition on Disability in Chronic Low Back Pain Patients: A Randomized Controlled Study. *Anaesthesia, Pain & Intensive Care*, v. 27, n. 1, p. 73–81, 1 dez. 2023.

VALENZA, MC *et al.* Results of a Pilates exercise program in patients with chronic non-specific low back pain: a randomized controlled trial. *Clinical Rehabilitation*, v. 31, n. 6, p. 753–760, 3 jun. 2017.

VALLE-JONES, J. C. *et al.* Controlled trial of a back support ('Lumbotrain') in patients with non-specific low back pain. *Current Medical Research and Opinion*, v. 12, n. 9, p. 604–613, 1992. Disponível em: <https://www.tandfonline.com/doi/abs/10.1185/03007999209111527>. Acesso em: 8 jun. 2026.

VAN POPPEL, Mireille N.M. *et al.* Lumbar supports and education for the prevention of low back pain in industry: a randomized controlled trial. *JAMA*, v. 279, n. 22, p. 1789–1794, 10 jun. 1998. Disponível em: <https://pubmed.ncbi.nlm.nih.gov/9628709/>. Acesso em: 8 jun. 2026.

VEERAMACHANENI, Ratnakar *et al.* Clinical Outcomes of a New Foot-Worn Non-Invasive Biomechanical Intervention Compared to Traditional Physical Therapy in Patients With Chronic Low Back Pain. A Randomized Clinical Trial. *Global Spine Journal*, v. 15, n. 6, p. 2890–2899, 15 jul. 2025.

VEIHELMANN, Andreas *et al.* Epidural neuroplasty versus physiotherapy to relieve pain in patients with sciatica: a prospective randomized blinded clinical trial. *Journal of orthopaedic science : official journal of the Japanese Orthopaedic Association*, v. 11, n. 4, p. 365–369, 2006. Disponível em: <https://pubmed.ncbi.nlm.nih.gov/16897200/>. Acesso em: 8 jun. 2026.

VELASCO-ROLDÁN, Olga *et al.* Immediate and Short-Term Effects of Kinesio Taping Tightness in Mechanical Low Back Pain: A Randomized Controlled Trial. *PM&R*, v. 10, n. 1, p. 28–35, 8 jan. 2018.

VERA-SAURA, Pablo *et al.* The Importance of Mind–Body in Pilates Method in Patients with Chronic Non-Specific Low Back Pain—A Randomized Controlled Trial. *Journal of Clinical Medicine*, v. 13, n. 16, p. 4731, 12 ago. 2024.

VERBRUGGHE, JONAS; AGTEN, ANOUK; STEVENS, SJOERD; HANSEN, DOMINIQUE; DEMOULIN, CHRISTOPHE; O. EIJNDE, BERT; *et al.* Exercise Intensity Matters in Chronic Nonspecific Low Back Pain Rehabilitation. *Medicine & Science in Sports & Exercise*, v. 51, n. 12, p. 2434–2442, dez. 2019.

VERBRUGGHE, Jonas; AGTEN, Anouk; STEVENS, Sjoerd; HANSEN, Dominique; DEMOULIN, Christophe; EIJNDE, Bert O.; *et al.* Exercise intensity matters in chronic nonspecific low back pain rehabilitation. *Medicine and Science in Sports and Exercise*, v. 51, p. 2434–2442, dez. 2019.

VERBRUGGHE, Jonas *et al.* High Intensity Training to Treat Chronic Nonspecific Low Back Pain: Effectiveness of Various Exercise Modes. *Journal of Clinical Medicine*, v. 9, n. 8, p. 2401, 27 jul. 2020.

VIJAYALAKSHMI, Ravilla *et al.* Effects of neural mobilization on sciatic nerve excursion, symptoms, and regional function in individuals with nerve-related low back pain. *Physiotherapy Quarterly*, v. 30, n. 3, p. 27–33, 9 maio 2022.

VILLATORO-LUQUE, Francisco Jesús *et al.* In non-specific low back pain, is an exercise program carried out through telerehabilitation as effective as one carried out in a physiotherapy center? A controlled randomized trial. *Musculoskeletal Science and Practice*, v. 65, p. 102765, jun. 2023.

VILLATORO-LUQUE, Francisco Jesús *et al.* Telerehabilitation for the treatment in chronic low back pain: A randomized controlled trial. *Journal of Telemedicine and Telecare*, v. 31, n. 5, p. 637–646, 30 jun. 2025.

VITMAN, Neta *et al.* A Comparison between Different Types and Frequency of Physiotherapy Treatment for Children and Adolescents with Postural Problems and Low Back Pain. *Physical & Occupational Therapy In Pediatrics*, v. 42, n. 2, p. 215–226, 4 mar. 2022.

WALEWICZ, Karolina *et al.* Effect of Radial Extracorporeal Shock Wave Therapy on Pain Intensity, Functional Efficiency, and Postural Control Parameters in Patients with Chronic Low Back Pain: A Randomized Clinical Trial. *Journal of Clinical Medicine*, v. 9, n. 2, p. 568, 19 fev. 2020.

WÄLTI, Philipp; KOOL, Jan; LUOMAJOKI, Hannu. Short-term effect on pain and function of neurophysiological education and sensorimotor retraining compared to usual physiotherapy in patients with chronic or recurrent non-specific low back pain, a pilot randomized controlled trial. *BMC Musculoskeletal Disorders*, v. 16, n. 1, p. 83, 10 dez. 2015.

WANG, Hongjiang *et al.* Effect of Progressive Postural Control Exercise Versus Core Stability Exercise in Young Adults with Chronic Low Back Pain: A Randomized Controlled Trial. *Pain and Therapy*, v. 12, n. 1, p. 293–308, 1 fev. 2023.

WANG, Shang-Quan *et al.* Clinical research on lumbar oblique-pulling manipulation in combination with sling exercise therapy for patients with chronic nonspecific low back pain. *Revista da Associação Médica Brasileira*, v. 65, n. 6, p. 886–892, jun. 2019.

WAONGENNGARM, Pooriput *et al.* Effects of an active break and postural shift intervention on preventing neck and low-back pain among high-risk office workers: a 3-arm cluster-randomized controlled trial. *Scandinavian Journal of Work, Environment & Health*, v. 47, n. 4, p. 306–317, 1 maio 2021.

WAQQAR, Saira; SHAKIL-UR-REHAMAN, Syed; AHMAD, Shakeel. McKenzie treatment versus mulligan sustained natural apophyseal glides for chronic mechanical low back pain. *Pakistan Journal of Medical Sciences*, v. 32, n. 2, 9 abr. 2016.

WAREŃCZAK, Agnieszka; LISIŃSKI, Przemysław. Does total hip replacement impact on postural stability? *BMC Musculoskeletal Disorders*, v. 20, n. 1, p. 229, 17 dez. 2019.

WARKE, K *et al.* Use of self-applied TENS for low back pain in people with multiple sclerosis. *International Journal of Therapy and Rehabilitation*, v. 11, n. 6, p. 275–280, jun. 2004.

WASEEM, Muhammad *et al.* Treatment of disability associated with chronic non-specific low back pain using core stabilization exercises in Pakistani population. *Journal of Back and Musculoskeletal Rehabilitation*, v. 32, n. 1, p. 149–154, 24 jan. 2019.

WATTANANON, Peemongkon *et al.* Effect of increased relative stiffness of the lumbar spine on hamstring muscle stretching in individuals with a history of low back pain suspected to have a clinical lumbar instability: A randomized crossover design. *Clinical Biomechanics*, v. 75, p. 104996, maio 2020.

WEBSTER, Lynn R. *et al.* Oxytrex Minimizes Physical Dependence While Providing Effective Analgesia: A Randomized Controlled Trial in Low Back Pain. *Journal of Pain*, v. 7, n. 12, p. 937–946, dez. 2006. Disponível em: <https://pubmed.ncbi.nlm.nih.gov/17157780/>. Acesso em: 8 jun. 2026.

WEINER, Debra K *et al.* Aging Back Clinics—a Geriatric Syndrome Approach to Treating Chronic Low Back Pain in Older Adults: Results of a Preliminary Randomized Controlled Trial. *Pain Medicine*, v. 21, n. 2, p. 274–290, 1 fev. 2020.

WEINER, Debra K. *et al.* Efficacy of percutaneous electrical nerve stimulation for the treatment of chronic low back pain in older adults. *Journal of the American Geriatrics Society*, v. 51, n. 5, p. 599–608, 1 maio 2003. Disponível em: <https://pubmed.ncbi.nlm.nih.gov/12752833/>. Acesso em: 8 jun. 2026.

WERNERS, Roland; PYNSENT, Paul B.; BULSTRODE, Christopher J.K. Randomized trial comparing interferential therapy with motorized lumbar traction and massage in the management of low back pain in a primary care setting. *Spine*, v. 24, n. 15, p. 1579–1584, 1 ago. 1999. Disponível em: <https://pubmed.ncbi.nlm.nih.gov/10457578/>. Acesso em: 8 jun. 2026.

WILLIAMS, Amanda *et al.* Effectiveness of a healthy lifestyle intervention for chronic low back pain: a randomised controlled trial. *Pain*, v. 159, n. 6, p. 1137–1146, 8 jun. 2018.

WITT, Claudia M. *et al.* Pragmatic randomized trial evaluating the clinical and economic effectiveness of acupuncture for chronic low back pain. *American journal of epidemiology*, v. 164, n. 5, p. 487–496, set. 2006. Disponível em: <https://pubmed.ncbi.nlm.nih.gov/16798792/>. Acesso em: 8 jun. 2026.

XIA, Ting *et al.* Similar Effects of Thrust and Nonthrust Spinal Manipulation Found in Adults With Subacute and Chronic Low Back Pain. *Spine*, v. 41, n. 12, p. E702–E709, 15 jun. 2016.

XIAO, Chongwu *et al.* Clinical Efficacy of Multimodal Exercise Telerehabilitation Based on AI for Chronic Nonspecific Low Back Pain: Randomized Controlled Trial. *JMIR mHealth and uHealth*, v. 13, p. e56176–e56176, 22 maio 2025.

XU, Simao *et al.* Interventional effect of core stability training on pain and muscle function of youth with chronic non-specific lower back pain: A randomized controlled trial. *Heliyon*, v. 10, n. 12, p. e32818, jun. 2024.

YALFANI, Ali *et al.* The effects of virtual reality training on postural sway and physical function performance on older women with chronic low back pain: A double-blind randomized clinical trial. *Journal of Back and Musculoskeletal Rehabilitation*, v. 37, n. 3, p. 761–770, 10 maio 2024.

YALFANI, Ali; ASGARPOOR, Azadeh. The effect of cognitive functional therapy on the plantar pressure distribution symmetry in patients with chronic non-specific low back pain: A randomized controlled trial. *Journal of Bodywork and Movement Therapies*, v. 40, p. 1454–1459, out. 2024.

YAMADA, Angela Shiratsu. Physiotherapeutic treatment associated with neuroscience of pain education for patients with chronic non-specific low back pain - single-blind randomized pilot clinical trial. *Ağrı - The Journal of The Turkish Society of Algology*, 2023.

YANG, Chao *et al.* Effects of percussive massage therapy on fascia echo intensity and fascia thickness in firefighters with chronic non-specific low back pain: a randomized controlled trial. *BMC Complementary Medicine and Therapies*, v. 24, n. 1, p. 390, 8 nov. 2024.

YANG, Hsueh-Hua *et al.* Laser acupuncture combined with auricular acupressure improves low-back pain and quality of life in nurses: A randomized controlled trial. *Journal of Integrative Medicine*, v. 21, n. 1, p. 26–33, jan. 2023.

YANG, Jinmo; SEO, Dongkwon. The effects of whole body vibration on static balance, spinal curvature, pain, and disability of patients with low back pain. *Journal of Physical Therapy Science*, v. 27, n. 3, p. 805–808, 2015.

YANG, Yang *et al.* A Tai chi and qigong mind-body program for low back pain: A virtually delivered randomized control trial. *North American Spine Society Journal (NASSJ)*, v. 20, p. 100557, dez. 2024.

YAO, Chongjie *et al.* Effects of Wuqinxi in the Patients with Chronic Low Back Pain: A Randomized Controlled Trial. *Evidence-Based Complementary and Alternative Medicine*, v. 2020, n. 1, 18 jan. 2020.

YEH, Bo-Yan *et al.* Efficacy of Electronic Acupuncture Shoes for Chronic Low Back Pain: Double-Blinded Randomized Controlled Trial. *Journal of Medical Internet Research*, v. 22, n. 10, p. e22324, 26 out. 2020.

YELLAND, Michael J. *et al.* Prolotherapy injections, saline injections, and exercises for chronic low-back pain: a randomized trial. *Spine*, v. 29, n. 1, p. 9–16, 1 jan. 2004. Disponível em: <https://pubmed.ncbi.nlm.nih.gov/14699269/>. Acesso em: 8 jun. 2026.

YILDIRIM, Ali *et al.* The effect of instrument-assisted soft tissue mobilization in lumbar disc herniation: A randomized controlled trial. *Journal of Bodywork and Movement Therapies*, v. 40, p. 424–429, out. 2024.

YILMAZ YELVAR, Gul Deniz *et al.* Is physiotherapy integrated virtual walking effective on pain, function, and kinesiophobia in patients with non-specific low-back pain? Randomised controlled trial. *European Spine Journal*, v. 26, n. 2, p. 538–545, 15 fev. 2017.

YIP, Y. B.; TSE, S. H.M. The effectiveness of relaxation acupoint stimulation and acupressure with aromatic lavender essential oil for non-specific low back pain in Hong Kong: A randomised controlled trial. *Complementary Therapies in Medicine*, v. 12, n. 1, p. 28–37, 2004. Disponível em: <https://pubmed.ncbi.nlm.nih.gov/15130569/>. Acesso em: 8 jun. 2026.

YOON, Yong-Soon *et al.* Effects of Personal Low-Frequency Stimulation Device on Myalgia: A Randomized Controlled Trial. *International Journal of Environmental Research and Public Health*, v. 19, n. 2, p. 735, 10 jan. 2022.

YOZBATIRAN, Nuray; YILDIRIM, Yucel; PARLAK, Bilge. Effects of fitness and aquafitness exercises on physical fitness in patients with chronic low back pain. *The Pain Clinic*, v. 16, n. 1, p. 35–42, 2004. Disponível em: <https://www.tandfonline.com/doi/abs/10.1163/156856904322858684>. Acesso em: 8 jun. 2026.

YU, Ji-Eun. Confirmation of the viability of a metaverse yoga class and investigation into the impact on pain, anxiety, and depression associated with low back pain after engaging in virtual yoga sessions. *Journal of Exercise Rehabilitation*, v. 20, n. 1, p. 24–33, 21 fev. 2024.

YU, Seong Hun *et al.* The effect of abdominal drawing-in exercise and myofascial release on pain, flexibility, and balance of elderly females. *Journal of Physical Therapy Science*, v. 28, n. 10, p. 2812–2815, 2016.

ZADRO, Joshua R. Appraisal of Clinical Practice Guideline: Clinical Practice Guideline for hamstring strain injury in athletes. *Journal of Physiotherapy*, v. 69, n. 2, p. 128, abr. 2023.

ZAFAR, Tasneem *et al.* Effects of progessive *vs* . constant protocol whole-body vibration on muscle activation, pain, disability and functional performance in non-specific chronic low back pain patients: a randomized clinical trial. *PeerJ*, v. 12, p. e18390, 24 out. 2024.

ZAFEREO, Jason *et al.* Regional manual therapy and motor control exercise for chronic low back pain: a randomized clinical trial. *Journal of Manual & Manipulative Therapy*, v. 26, n. 4, p. 193–202, 8 ago. 2018.

ZAHRA MASOOD *et al.* Effect of lumbar traction on discogenic low back pain using variable forces. *Journal of the Pakistan Medical Association*, v. 72, n. 3, 3 fev. 2022.

ZAKARI, Usman Usman *et al.* Comparison of the Effects of Positional Release Therapy and Lumbar Stabilization Exercises in the Management of Chronic Mechanical Low Back Pain-Randomized Controlled Trial. *Critical Reviews in Physical and Rehabilitation Medicine*, v. 31, n. 4, p. 321–332, 2019.

ZAMANI, Hamid *et al.* Effects of External Focus and Motor Control Training in Comparison with Motor Control Training Alone on Pain, Thickness of Trunk Muscles and Function of Patients with Recurrent Low Back Pain: A Single Blinded, Randomized Controlled Trial. *Archives of Bone and Joint Surgery*, v. 10, p. 766–774, 2022.

ZARZYCKA, Katarzyna; ZARZYCKI, Maciej; KOLASA, Paweł. Comparative evaluation of rehabilitation systems for patients with chronic lumbar overload syndrome. *Aktualności Neurologiczne*, v. 18, n. 1, p. 5–13, 29 maio 2018.

ZAWORSKI, Kamil; LATOSIEWICZ, Robert. The effectiveness of manual therapy and proprioceptive neuromuscular facilitation compared to kinesiotherapy: a four-arm randomized controlled trial. *European Journal of Physical and Rehabilitation Medicine*, v. 57, n. 2, maio 2021.

ZERBINI, C. *et al.* Efficacy of etoricoxib 60 mg/day and diclofenac 150 mg/day in reduction of pain and disability in patients with chronic low back pain: results of a 4-week, multinational, randomized, double-blind study. *Current medical research and opinion*, v. 21, n. 12, p. 2037–2049, dez. 2005. Disponível em: <https://pubmed.ncbi.nlm.nih.gov/16368055/>. Acesso em: 8 jun. 2026.

ZHANG, Chanjuan *et al.* Alterations in functional connectivity in patients with non-specific chronic low back pain after motor control exercise: a randomized trial. *European Journal of Physical and Rehabilitation Medicine*, v. 60, n. 2, abr. 2024.

ZHENG, Fuming *et al.* Long-term impact of self-compassion training with core stability exercise on patients with nonspecific chronic low back pain: A randomized controlled trial. *Journal of Psychosomatic Research*, v. 181, p. 111678, jun. 2024.

ZHENG, Yi-Li *et al.* The Effects of Whole-Body Vibration Exercise on Anticipatory Delay of Core Muscles in Patients with Nonspecific Low Back Pain. *Pain Research and Management*, v. 2021, p. 1–10, 3 ago. 2021.

ZUCCO, F; ORLANDI, B. Very low frequency and low intensity magnetic fields in a different treatment of disease: Chronic low back pain in senile degenerative arthritis: A double blind study. *Bioelectrochemistry and Bioenergetics*, v. 14, n. 1–3, p. 187–195, 1985.

ZUREK, Grzegorz *et al.* Vibrating Exercise Equipment in Middle-Age and Older Women with Chronic Low Back Pain and Effects on Bioelectrical Activity, Range of Motion and Pain Intensity: A Randomized, Single-Blinded Sham Intervention Study. *Biology*, v. 11, n. 2, p. 268, 8 fev. 2022.
